# Foldamer-mediated manipulation of a pre-amyloid toxin

**DOI:** 10.1038/ncomms11412

**Published:** 2016-04-25

**Authors:** Sunil Kumar, Melissa Birol, Diana E. Schlamadinger, Slawomir P. Wojcik, Elizabeth Rhoades, Andrew D. Miranker

**Affiliations:** 1 Department of Molecular Biophysics and Biochemistry, Yale University, 260 Whitney Avenue, New Haven, Connecticut 06520-8114, USA; 2 Department of Chemical and Environmental Engineering, Yale University, 260 Whitney Avenue, New Haven, Connecticut 06520-8114, USA

## Abstract

Disordered proteins, such as those central to Alzheimer's and Parkinson's, are particularly intractable for structure-targeted therapeutic design. Here we demonstrate the capacity of a synthetic foldamer to capture structure in a disease relevant peptide. Oligoquinoline amides have a defined fold with a solvent-excluded core that is independent of its outwardly projected, derivatizable moieties. Islet amyloid polypeptide (IAPP) is a peptide central to β-cell pathology in type II diabetes. A tetraquinoline is presented that stabilizes a pre-amyloid, α-helical conformation of IAPP. This charged, dianionic compound is readily soluble in aqueous buffer, yet crosses biological membranes without cellular assistance: an unexpected capability that is a consequence of its ability to reversibly fold. The tetraquinoline docks specifically with intracellular IAPP and rescues β-cells from toxicity. Taken together, our work here supports the thesis that stabilizing non-toxic conformers of a plastic protein is a viable strategy for cytotoxic rescue addressable using oligoquinoline amides.

## Abstract (web summary)

Intrinsically disordered proteins that form amyloid fibrils are hard to target with traditional therapeutic approaches. Here, the authors report on an oligoquinoline derivative that binds the human islet amyloid polypeptide, stabilising an alpha-helical structure that reduces its cellular toxicity.

## Graphical abstract

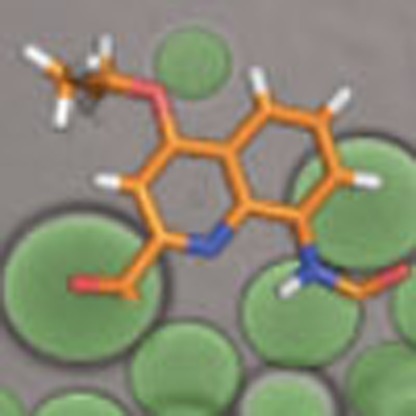

Biopolymers are distinguished from artificial polymers by the presence of a specified sequence of precursors. A discrete sequence affords biopolymers the capacity to fold to a specific structure despite having an astronomically sized conformational landscape. A tiny set of precursors (4 and 20 for RNA and proteins, respectively) create a breadth of folds and functions essential to life. In contrast, synthetic polymers have access to an essentially unlimited array of precursor variants; however, the lack of sequence control and unique conformation results in a breadth of function that is dwarfed by biology.

Synthetic foldamers seek to join the best of these two worlds^1^. New scaffolds with specifiable sequences permit folded and functional structures to be successfully designed. For instance, foldamers based on oligomers of arylamides, β-peptide, α/β-peptide and peptoids have been designed to be an antimicrobial agent^2^, agonist of GLP-1 receptor^3^, an inhibitor of HIV-cell fusion^4^ and an antagonist of vascular endothelial growth factor receptor 2 (ref. 5), respectively. The achievable specificities of these small molecules rival biopolymers. For example, a quinoline-based aromatic foldamer was recently developed using an iterative modification approach to selectively and stereospecifically encapsulate D-fructose^6^.

Dynamic binding modes, such as conformational selection, are often observed in protein:ligand interactions^7^. A recent example is the anti-cancer drug Gleevec^8^. Gleevec binds to structurally identical sites in Src and its homologue, Abl. Nevertheless, there is a >1,000-fold difference in binding affinity that can be accounted for by differences in protein dynamics. It stands to reason that the internal degrees of freedom of ligands should be similarly exploitable. However, ligand dynamics are viewed unfavourably as a result of entropy loss on binding. The non-covalent and reversible stabilization of foldamer structure presents an opportunity to limit entropic loss on protein binding without sacrificing opportunities and potential benefits of dynamic sampling.

A novel tetraquinoline amide foldamer, ADM-116, has been developed to counter conformational transitions in islet amyloid polypeptide (IAPP). IAPP is a 37-residue peptide co-packaged with insulin in pancreatic β-cells. In type II diabetes, aggregation of this peptide into amyloid fibres is observed, and pre-amyloid states of IAPP are toxins resulting in β-cell death^9^. ADM-116 displays activity across solution and cell-based assays, as a result of structure-specific binding to pre-amyloid states. Unique to ADM-116 is its capacity to cross the plasma membrane, without assistance from cellular processes, and antagonize toxicity long after the cellular uptake of IAPP is complete. Passive translocation is remarkable because ADM-116 is dianionic and further, can carry an anionic fluorescent cargo across the plasma membrane: properties otherwise observed only for polycations. These gains of function are dependent on the capacity of the foldamer to not only recognize IAPP but also to transiently sample partially folded states.

## Results

### Time-dependent localization of IAPP

**Figure 1:**
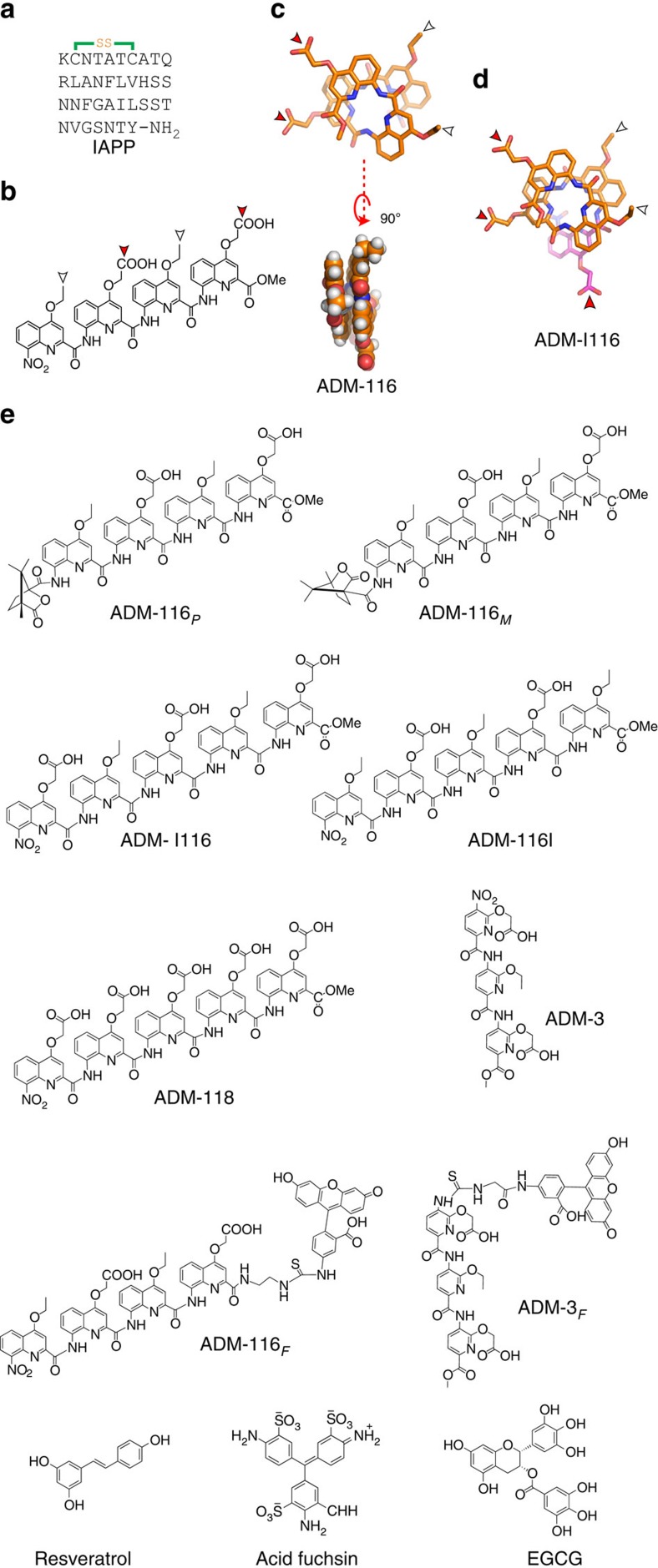
Structures of compounds. (**a**) Primary sequence of human IAPP. Wild-type IAPP is C-terminally amidated and has an intramolecular disulfide bond between residues 2 and 7. (**b**) Chemical structure of ADM-116. (**c**) Three-dimensional stick (top) and sphere (bottom) model of ADM-116. (**d**) Pentaquinoline, ADM-|116, in which ADM-116 is extended one residue from the N terminus. Arrows in **a**,**b** and **d** indicate surface-exposed functional moieties. Structures in **b** and **d** adapted from a longer oligoquinoline^47^. (**e**) Chemical structures of other molecules used in this study.

**Figure 2:**
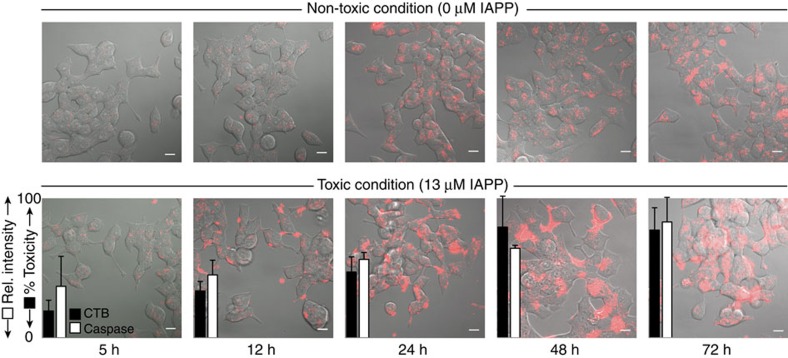
Time-dependent uptake of IAPP and toxicity. At time zero, 100 nM IAPP_A594_ with (toxic) and without (non-toxic) an additional 13 μM of unlabelled IAPP was added to culture media of INS-1 cells. Confocal images were taken at the indicated time points. Three biological replicates were performed. Scale bar, 10 μm. Inset: colourimetric evaluation of toxicity (CellTiter Blue) and apoptosis (using caspase-3) at the toxic condition relative to vehicle-only controls. Each time point is the average of eight on-plate repeats from each of three independently performed replicates (*n*=24). rel., relative.

INS-1 cells were incubated with 100 nM IAPP (Fig. 1a) labelled at its N terminus with Alexa-594 (IAPP_A594_). Co-addition of 0 or 13 μM unlabelled IAPP corresponds to non-toxic and toxic conditions, respectively. After 5 h under non-toxic conditions, IAPP is not significantly internalized (Fig. 2). By 12 h, intracellular IAPP is readily observed with maximum extent of internalization apparent at 24 h (Fig. 2). At all time points under this non-toxic condition, IAPP appears as diffuse puncta, possibly a consequence of the energy-dependent cellular uptake under these conditions^10^. Under toxic conditions, slightly elevated uptake of IAPP is apparent by 5 h. By 12 h, contrasting behaviour can be clearly seen with the toxic condition showing external and internalized puncta and larger assemblies^11^. By 24 h, extracellular IAPP is a small fraction of the total IAPP. At 48 h, large intracellular aggregates appear and continue to increase in intensity by 72 h. This progression suggests that under these conditions, self-assembly is mediated by the intracellular environment, consistent with the work showing the culture media and plasma membrane to be inhibitory to amyloid formation^12^.

Cytotoxicity is mediated by intracellular IAPP. The time dependence of IAPP-mediated toxicity was measured in parallel to imaging. Using caspase-3/7 activity as an indicator of apoptosis, the effects are apparent as early as 5 h with steady increases over the time course of the observation (Fig. 2). The fraction of cells affected by IAPP can be approximated by monitoring total cytosolic reductase activity relative to IAPP-free controls. The trend clearly parallels the apoptosis marker. Importantly, continued increases in apparent apoptotic activity are evident long after the 24 h time point, where little extracellular IAPP is present (Fig. 2). Taken together, this suggests the site of toxicity is intracellular. *In vivo*, it is debated whether IAPP becomes toxic before or after secretion^9^,^13^. Certainly, it is possible that IAPP has multiple mechanisms and locations for inducing toxic gains of function. The cell-penetrating peptide quality of IAPP, however, makes such determinations particularly challenging, and possibly irrelevant^14^. Nevertheless, it is clear that the small-molecule modulation of IAPP requires a compound that can be internalized.

### Targeting membrane-bound IAPP

**Figure 3:**
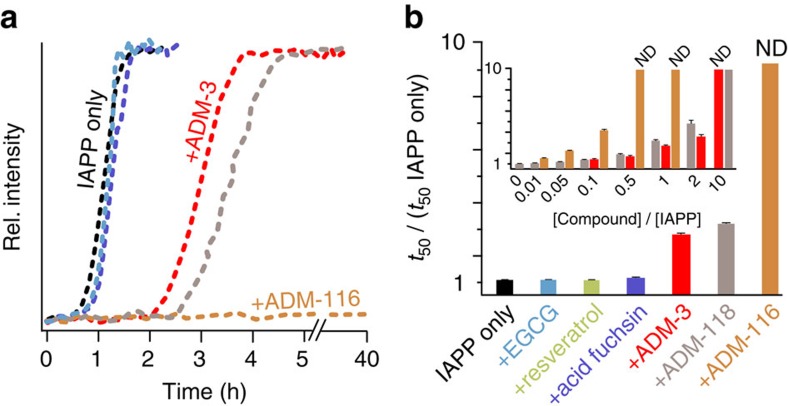
Effect of ligands on the kinetics of IAPP fibre formation. Representative (**a**) and statistics of reaction midpoints (*t*50, **b**) for 10 μM IAPP fibre formation reactions catalysed by unilamellar vesicles (630 μM monomer units, DOPG:DOPC, 1:1, 100 nm). (**b**) Inhibition with the indicated small molecules is performed at 1:1 IAPP:small molecule. Colours are consistent between the panels. Inset: statistics of dose dependence of inhibition of fibre formation by ADM-116, ADM-3 and ADM-118. An absence of kinetics over the time course of observation (for example, >40 h for ADM-116 at 1:1) are indicated as not detected (ND). All experiments were conducted at least in triplicate with errors reported as±1 s.d. rel., relative.

We hypothesize that the compounds that target intracellular IAPP are more likely to be found among membrane active inhibitors of IAPP self-assembly^15^. In solution, the assembly of 10 μM IAPP into fibres occurs with a reaction midpoint, *t*_50_, of 18±1.4 h (Supplementary Fig. 1). An otherwise identical reaction conducted in the presence of large unilamellar vesicles (LUVs; composed of a 1:1 mixture of DOPG:DOPC) yields a *t*_50_ of 1.1±0.1 h (Fig. 3a). These LUVs possess a surface anionicity of 50%. Comparable catalysis is apparent at 7:3, DOPC:DOPG, which is closer to the anionic content of cell membrane phospholipids (Supplementary Fig. 2)^16^,^17^. In previous work, we have successfully used anionicities ⩾50% to generate insights for IAPP that proved applicable to live cells^18^,^19^. In a study most relevant to our work here, we used LUVs identical to those presented here to establish cross-correlation of membrane-bound α-helical conformers of IAPP, inhibition of several solution biophysical-based gains of function and cytotoxic rescue^20^. We further note that detergent induced α-helical oligomers of IAPP recently enabled the discovery of conformation-specific antisera in human diabetics^21^. In this work, we use the LUV-based assay as a reference condition under which small molecules can be assessed for activity on IAPP in the context of a lipid bilayer.

Synthetic foldamers based on quinoline have been shown to hold promise in solution biophysical assays of IAPP^22^. Synthesis and screening (Methods; Supplementary Methods) of numerous variants allowed us to identify one derivative, ADM-116 (Fig. 1b,c), with marked activity in LUV-catalysed fibrillation assays. At 1:1 (IAPP:ADM-116), liposome-catalysed fibrillation is undetectable (a *t*_50_ >40-fold higher than control) with significant inhibition observable even at 1:0.1 (Fig. 3a,b). Amyloid formation is also not observed by electron microscopy (Supplementary Fig. 3) and far-ultraviolet circular dichroism (CD; Fig. 5d). In comparison, our compounds ADM-3 (ref. 20) and OQ5 (referred to in this work as ADM-118) (refs 22, 23) inhibit by factors of 2.7±0.1- and 3.3±0.1-fold, respectively. Other compounds such as epigallocatechin gallate (EGCG)^24^, acid fuchsin^25^ and resveratrol^26^, show no detectable effect on liposome-catalysed amyloid assembly (Fig. 3a,b). Clearly, ADM-116 contains functional moieties, the steric and physicochemical properties of which result in exceptional inhibition of lipid-catalysed amyloid assembly.

**Figure 4:**
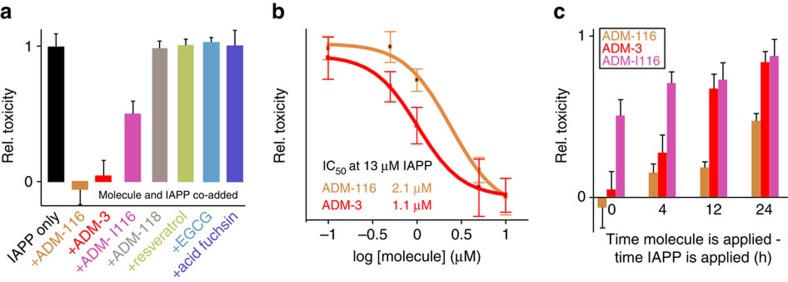
Rescue of INS-1 cells from IAPP-induced toxicity. (**a**) Statistics of the toxic effect of 13 μM IAPP applied at time zero to INS-1 cells and measured 48 h later using CellTiter Blue. Data are shown for IAPP alone and with equimolar ratio of the indicated small molecule co-added at a 1:1 ratio. Data are renormalized to the toxicity induced only by IAPP. Each condition is the average of eight on-plate repeats from each of three independently performed replicates (*n*=24). (**b**) Dose dependence of toxic rescue by ADM-3 and ADM-116. (**c**) Relative toxicity of 13 μM IAPP in which a time delay is introduced between the addition of IAPP and addition of molecule. (**a**–**c**) Error bars are s.d.'s from three sets of experiments conducted on separate occasions. rel., relative.

ADM-116 rescues IAPP-induced toxicity. After 48 h of incubation with 13 μM IAPP, INS-1 viability is decreased 78±8%. Co-addition of ADM-116 at a stoichiometric ratio of 1:1 (IAPP:ligand) wholly restores viability (Fig. 4a,b). The compound ADM-3 (ref. 20), a tripyridylamide, is also active on membrane-bound IAPP and also restores cell viability (Fig. 4a) with a dose dependence comparable to ADM-116 (Fig. 4b). Toxic rescue by ADM-116 and ADM-3 is also reproducible in a non-pancreatic cell line, COS-1 (Supplementary Fig. 4). Our previously published compound, ADM-118, as well as resveratrol^26^, EGCG^24^ and acid fuchsin^25^ were all ineffective in rescuing toxicity under these conditions. Published conditions show that cytotoxic rescue can be achieved following a procedure in which IAPP and small molecule are preincubated for ⩾11 h before adding the complexes to cell culture^24^,^25^. Following this procedure, all of these molecules significantly rescue IAPP-mediated toxicity in INS-1 cells (Supplementary Fig. 5). In contrast, we co-introduce IAPP and small molecule to cell culture. At concentrations investigated in this report, none of the molecules show intrinsic toxicity towards INS-1 cells nor interfered with the colourimetric assay (Supplementary Fig. 6).

ADM-116, and not ADM-3, rescues cells from intracellular IAPP toxicity. In light of the intracellular origins of toxicity indicated above (Fig. 2), cell viability was instead assessed under conditions in which introduction of IAPP was followed by a delay before the addition of small molecule. Remarkably, even after a delay of 24 h, ADM-116 is capable of rescuing IAPP-induced cytotoxicity by 47±4%. In marked contrast, ADM-3 was ineffective in rescuing toxicity when added after 12 h (Fig. 4c). As IAPP is internalized by 24 h of incubation (Fig. 2), this suggests that the mechanism of ADM-116 rescue includes penetration of the plasma membrane.

### Molecular specificity of binding

**Figure 5:**
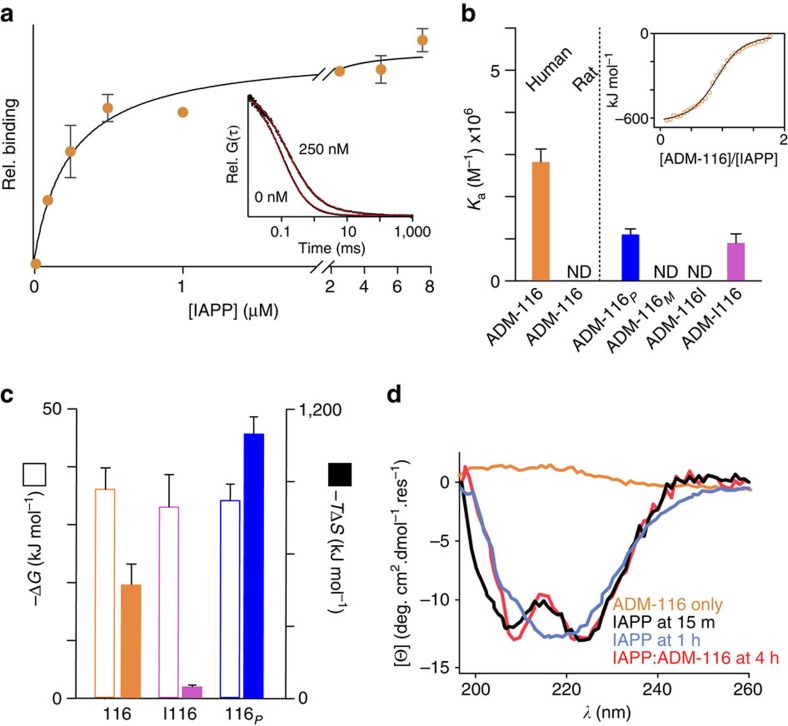
Small molecule:IAPP binding and structural effects. (**a**) Relative binding of ADM-116 to IAPP using 25 nM ADM-116_*F*_, and measuring diffusion by fluorescence correlation spectroscopy as a function of [IAPP]. Inset: representative raw autocorrelation data for ADM-116_*F*_ in the presence of 0 nM and 250 nM IAPP. Fits using a single diffusing species for data at 0 nM IAPP and two diffusing species at 250 nM IAPP are shown in red. The data shown are an average of three experimental repeats. (**b**) Association constants for the indicated small molecule with human IAPP measured by ITC. Note, undetectable binding indicated with ‘ND'. Inset: representative raw data and fit for ADM-116 binding plotted as a function of stoichiometry. (**c**) Free energy and entropy contributions to binding by ITC for the indicated compounds. (**d**) Far-ultraviolet CD spectra of 25 μM IAPP in the presence of LUVs (1.2 mM in monomeric units, DOPG:DOPC, 1:1, 100 nm) expressed as mean residue ellipticity (MRE). Shown are two time points for IAPP alone (blue, black) and with addition of stoichiometric ADM-116 at *t*=0. ADM-116 without IAPP is shown as control (orange). rel., relative; ND, not detected.

The complex formed by ADM-116 and IAPP is small and monodisperse. A fluorescein derivative of ADM-116, ADM-116_*F*_, was prepared by coupling at its C terminus and then monitored by fluorescence correlation spectroscopy. Autocorrelation profiles were readily fit to a single diffusing species with a diffusion time, *τ*D, for ADM-116_*F*_ measures 130±10 μs (Fig. 5a) with no apparent presence of soluble aggregates. These observations remained consistent from 25 to 13 μM, the latter corresponding to concentrations used in cell culture (Supplementary Fig. 7a). Self-association and aggregation are similarly absent at 20 μM ADM-116, as measured by nuclear magnetic resonance (NMR; Supplementary Fig. 7b). On titration of a 25 nM solution of ADM-116_*F*_ with IAPP, a second component becomes evident with a *τ*D=∼400 μs (Fig. 5a). The diffusion time of this slower component was consistent over the course of the titration, indicative of a discrete complex. Fitting the fractional amplitude of bound to unbound ADM-116_*F*_ gives a *K*_d_=240±60 nM. Isothermal titration calorimetry (ITC) using unlabelled IAPP and ADM-116 yields an exothermic profile that fits a one-site binding model with a *K*_a_=2.8±0.3 × 10^6^ M^−1^ (*K*_d_=360±40 nM; Fig. 5b; Supplementary Table 1). Plainly, ADM-116 forms a discrete complex with strong affinity to IAPP.

ADM-116 stabilises the α-helical subdomain of IAPP. The far-ultraviolet CD spectrum of membrane-bound IAPP exhibits two minima near 208 and 222 nm, characteristic of α-helical structure^27^ (Fig. 5d). This profile transitions to that of β-sheet, characteristic of amyloid, within 1 h. In contrast, IAPP remains predominantly α-helical even after 4 h when incubated with ADM-116 (1:0.5, IAPP:ADM-116). α-Helical structure within the membrane-binding subdomain of IAPP has been mapped to segments containing most of the first 22 residues of IAPP^27^,^28^. Within this stretch, the sequence variant of IAPP from rat, rIAPP, varies only at position 18 (H18R). Binding of ADM-116 by rIAPP was not detectable (Fig. 5b). An additional five residues differ between hIAPP and rIAPP over residues 23–29. Amyloid nucleation in full-length hIAPP is mediated, in part, by residues 20–29 (ref. 29). The kinetic profile of amyloid formation by 200 μM of the 10-residue sub-peptide, IAPP_20–29_, gives a *t*_50_ of 6.6±0.3 h (Supplementary Fig. 8), consistent with our earlier work^22^. The presence of 1:1 ADM-116 has little effect on the independent amyloid assembly of IAPP_20–29_. Thus, ADM-116 does not functionally interact with human residues 20-29 nor does it detectably bind to rIAPP. Combined with the observation of membrane-bound α-helical stabilization, this suggests that ADM-116 binds to the α-helical subdomain of IAPP.

### Specificity of intracellular binding

**Figure 6:**
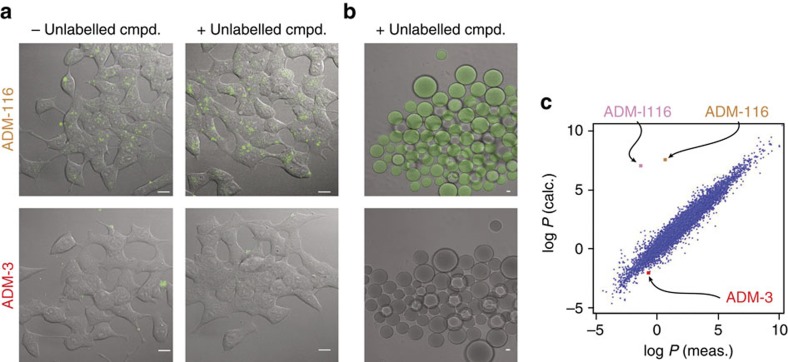
Membrane translocation and partitioning. (**a**) Confocal fluorescence imaging of INS-1 cells incubated for 12 h with 200 nM ADM-116_*F*_ or 200 nM ADM-3_*F*_. Conditions shown are with and without addition of a further 15 μM of unlabelled small molecule. (**b**) GPMVs prepared from INS-1 cells incubated for 24 h with 200 nM ADM-116_*F*_ or ADM-3_*F*_ mixed with 10 μM of unlabelled compound. Scale bars, 5 μm (**a**,**b**). (**c**) Calculated versus measured octanol:water partition coefficients for three molecules used in this work (prepared using MolInspiration, http://www.molinspiration.com). United States approved pharmacopeia shown as blue dots. calc., calculated; cmpd., compound.

To gain mechanistic insight, ADM-116 was applied to INS cells and visualized by confocal microscopy. ADM-116_*F*_ at non-rescue (200 nM) and rescue (200 nM + 15 μM ADM-116) concentrations is able to penetrate the cell membrane (Fig. 6a). In contrast, fluorescein-labelled ADM-3 (ADM-3_*F*_) showed no capacity to populate the cell interior (Fig. 6a). Approximately, 1,800 peptides have been shown to behave as cell-penetrating peptides (CPPs) capable of not only crossing the membranes but facilitating the transport of cargo such as a fluorophore^30^. These peptides tend to be short (10–20 amino acids), strongly cationic (5–10 basic amino acids) and require concentrations >10 μM for cell penetration to occur without cellular assistance^31^. Cationic oligoquinolines have recently been shown to have properties in keeping with most CPPs^32^. For example, an octaquinoline with eight positive charges, but not a +4 tetraquinoline penetrates cells with energy-independent penetration reported at 100 μM. Here to mimic a cell surface devoid of active endocytosis, giant plasma membrane-derived vesicles (GPMVs) were prepared from live INS-1 cells^12^,^33^. ADM-116, and not ADM-3, shows a clear capacity to penetrate (Fig. 6b). This is remarkable as ADM-116 is anionic (−2), devoid of positive charges, and yet capable of crossing the bilayer at 200 nM while also carrying a negatively charged fluorophore. Overall, ADM-116 likely enters cells via both active- and energy-independent pathways. The mechanism for the latter, however, appears to be fundamentally different than virtually all CPPs reported to date.

**Figure 7:**
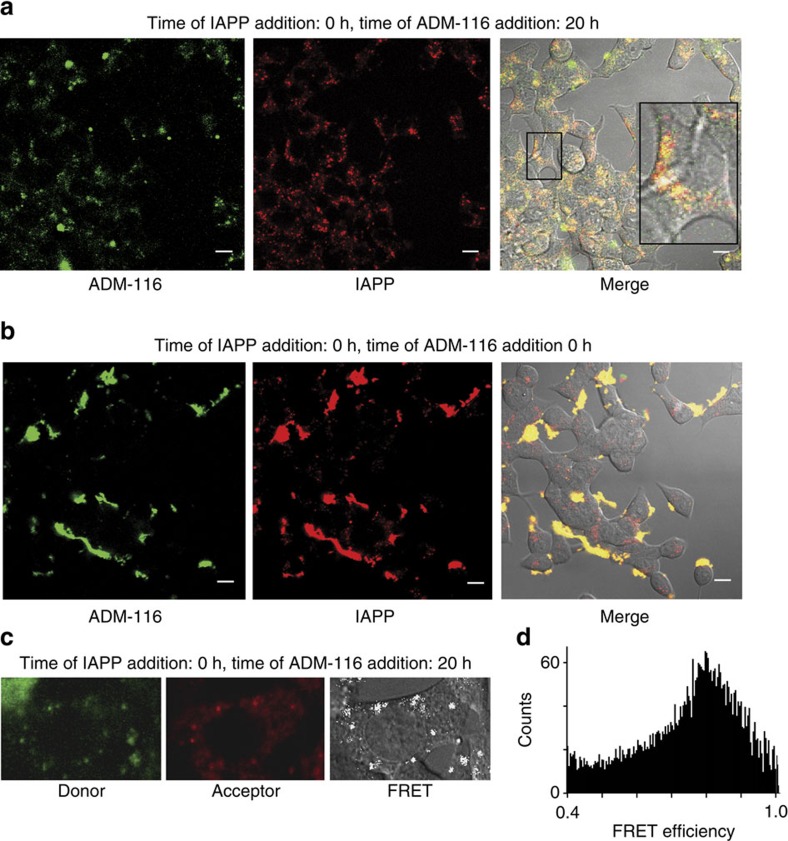
Co-localization of IAPP and ADM-116. (**a**,**b**) INS-1 cells were exposed to a rescued condition doped with fluorescent variants of protein and ligand (13 μM IAPP, 15 μM ADM-116, 100 nM IAPP_A594_ and 200 nM ADM-116_*F*_). In **a**, small molecule and IAPP are co-introduced to the culture media. In **b**, the ligand is added 20 h after protein. Scale bars, 10μm. (**c**) as in **b**, but without the unlabelled components and initial incubation with IAPP was performed for 18 h. Confocal fluorescence images were collected at the acceptor's emission wavelength using donor (left) or acceptor excitation light (middle). FRET was computed from these channels and is shown as white dots on a DIC image of a representative cell. Only areas showing a FRET efficiency of >0.4 are shown as values below this are indistinguishable from background. Background FRET was determined using parallel experiments that included a further 13 μM of unlabelled IAPP (Supplementary Fig. 11). (**d**) FRET histogram depicting the total counts at the indicated FRET efficiencies across ∼50 regions of interest. The data shown have been repeated three times. The FRET efficiency calculated is an average of fifty cells imaged, performed across three independent biological replicates.

Cellular rescue is associated with co-localization of protein and small molecule. INS-1 cells were treated with toxic concentrations of IAPP (100 nM IAPP_A594_ and 13 μM IAPP). After 20 h, rescuing concentrations of ADM-116 (200 nM ADM-116_*F*_ and 15 μM ADM-116) were added. In rescued cells, IAPP co-localizes with ADM-116 and fewer IAPP aggregates are observed (Fig. 7a). An alternative mechanism operates under conditions in which IAPP and ADM-116 are co-introduced. Although IAPP and ADM-116 can separately enter cells (Figs 2 and 6a), when added together, they co-localize on the cell surface (Fig. 7b). A similar observation can be made for ADM-3 (Supplementary Fig. 9). This suggests that the interaction of ADM-116 or ADM-3 with IAPP prohibits the entry of toxic peptide into the cells. In contrast, rescue by delayed addition of ADM-116 appears to disrupt internal toxic aggregate formation.

Intracellular rescue is a consequence of direct interactions between IAPP and ADM-116. Förster resonance energy transfer (FRET) measurements were made in live cells. Here 200 nM ADM-116_*F*_ was applied to INS-1 cells 20 h after 100 nM IAPP_A594_. Energy transfer from ADM-116_*F*_ to IAPP_A594_ is readily apparent and intracellular (Fig. 7c). Importantly, statistical assessment of the FRET reveals a Gaussian distribution strongly consistent with interactions not only being close (∼40 Å) but also discrete and therefore specific (Fig. 7d).

### Structure dynamics and function

ADM-116 is a folded molecule with a hydrophobic core. For Food and Drug Administration-approved drugs, measured and calculated octanol–water partition coefficients are closely comparable (Fig. 6c). The predicted and experimental log *P* for ADM-3 are −0.7 and −2.0, consistent with its practical solubility in buffer (Fig. 6c). For ADM-116, however, there is a seven order of magnitude disparity. The calculated log *P* is 7.6 (comparable to cholesterol), while the measured log *P* is 0.6. This anomaly is a consequence of the folded nature of ADM-116. Overall, ADM-116 is anionic (−2) and water soluble, yet capable of passive cell membrane penetration. This suggests a paradigm for cell penetration in which dynamic conformational change of the small molecule is central to this process.

**Figure 8:**
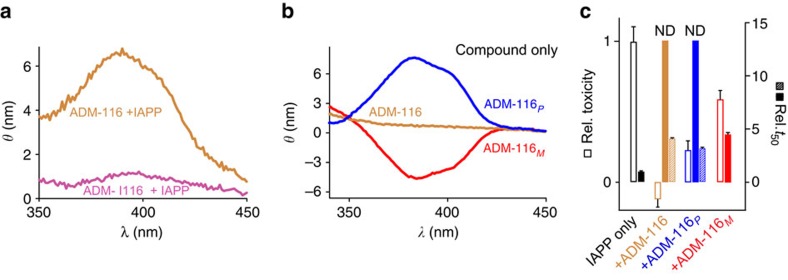
Selection, perturbation and activity of ADM-116 enantiomers. (**a**) Visible CD of 25 μM ADM-116 or ADM-|116 in the presence of 25 μM IAPP. (**b**) Visible CD of ADM-116 and each of two chiral variants designed to introduce bias into the ADM-116 enantiomer distribution. (**c**) Comparison of the fibre formation inhibition (closed) and toxic rescue (open) activities of enantiomer-biased constructs of ADM-116. Experimental conditions match those used in Fig. 3 and Fig. 4, respectively. Fibre formation inhibition also shown for inhibition of reactions conducted at 10:1 IAPP:small molecule (hash).

The distribution of quinoline conformations is affected by ADM-116:IAPP binding. Conformational dynamics permits compact oligoquinolines to sample an equal mixture of right-(*P*) and left-(*M*) mirror-image helices^34^. The CD spectrum of ADM-116 in aqueous buffer contains equal contributions from these two hands resulting in a flat line at 390 nm (Fig. 8b). In marked contrast, the CD spectra of ADM-116 recorded in the presence of stoichiometric IAPP (Fig. 8a) shows positive ellipticity (Fig. 8a). Binding clearly shifts the tetraquinoline equilibrium between right- and left-handed helical states.

IAPP binds preferentially to the (P) conformer of ADM-116. Following an established method^35^, two analogues were synthesized in which the chiral camphanyl groups were coupled with the N terminus of ADM-116 (Fig. 1e). CD spectra confirm the induction of helical bias (Fig. 8b) with ADM-116_*P*_ and ADM-116_*M*_ defined as the derivatives that give positive and negative profiles at 390 nm, respectively. Importantly, the intensity of ADM-116 in the presence of IAPP is within 90% that of ADM-116_*P*_ (Fig. 8a; Supplementary Fig. 10). By ITC, the *K*_a_ of IAPP to ADM-116_*P*_ is 1.1±0.1 × 10^6^ M^−1^; within a factor of three of ADM-116. In contrast, binding was not detectable for ADM-116_*M*_ (Fig. 5b). This pattern is evident in diverse assays. For lipid-catalysed fibrillation reactions, ADM-116_*P*_ is as potent an inhibitor as ADM-116, while ADM-116_*M*_ inhibits fibrillation only weakly (Fig. 8c). In cell toxicity assays, ADM-116_*P*_ shows ∼2.4 times greater inhibition of toxicity than ADM-116_*M*_ (Fig. 8c). Thus, in solution biophysical assays and in toxic rescue, IAPP:ADM-116 interactions are stereospecific.

IAPP binding to ADM-116 includes interactions with the C-terminal end of the ADM-116 helix. Enantiomeric interconversion in oligoquinolines is more restricted with increasing length^35^,^36^. Here two molecules were synthesized in which ADM-116 was lengthened by one subunit either at the N-terminal (ADM-|116) or C-terminal (ADM-116|) end. The lateral surfaces of ADM-|116 and ADM-116| necessarily include the surface of ADM-116 (Fig. 1d,e). If IAPP:ADM-116 binding includes contact with the ends of the quinoline helix, then binding to one of the two variants should be more strongly affected. The *K*_a_ of IAPP:ADM-|116 is similar to ADM-116 (*K*_a_=0.9±0.2 × 10^6^ M^−1^). This binding induces a random coil to α-helix transition comparable to ADM-116 (Supplementary Fig. 10b). Note also that the binder, ADM-116_*P*_, is also an N-terminal derivative. In marked contrast, no binding is detected for ADM-116|. This indicates that IAPP interacts with the C-terminal end of ADM-116.

Intracellular toxic rescue by ADM-116 is dependent on oligoquinoline length. In toxicity studies, the co-addition of ADM-|116 and IAPP results in diminished toxicity relative to IAPP alone (Fig. 4a). However, if ADM-|116 is added to INS-1 cells after IAPP is internalized, rescue is no longer observed (Fig. 4c). In other words, the addition of a fifth quinoline to ADM-116 results in a molecule that retains activities comparable to ADM-3, that is, a compound that is an effective antagonist provided IAPP and small-molecule encounter each other in the extracellular environment.

Differences in time-dependant rescue by ADM-|116 and ADM-116 can be mapped to the thermodynamic stabilities of the molecules. We observe the ΔΔ*G* of IAPP binding to ADM-**|**116 versus ADM-116 to be ∼3 kJ mol^−1^ (Fig. 5c; Supplementary Table 1). Strikingly, changes to entropic contributions (Δ*T*Δ*S*) are ∼400 kJ mol^−1^ (Fig. 5c). The large difference in entropic contributions resembles a hydrophobic effect similar to observations for globular protein folding. Structural assessment of the IAPP:ADM-**|**116 complex by CD shows weak ellipticity at 390 nm after only ∼2.5 h compared with IAPP:ADM-116. This level of ellipticity persists even after days of incubation (Fig. 8a), indicating that equilibrium has been reached and therefore a reduction in the capacity to shift the enantiomer equilibrium. Note also that as a complex, IAPP:ADM-116 loses cell-penetrating function (Figs 6a and 7b). Taken together, these observations suggest that ADM-116 exists predominantly in its folded state, but is able to sample partially folded states, a subset of which, are relevant to its membrane crossing activities.

## Discussion

In this work, we have shown oligoquinoline-mediated perturbation of an intracellular pre-amyloid toxin resulting from stabilization of a weakly sampled conformation. IAPP is not only a target relevant to the pathology of diabetes but also an exemplar of intrinsically disordered proteins (IDPs) and peptide subdomains of larger systems. The widespread importance of IDPs to areas spanning cell signalling and pathological self-assembly make them a key target for basic and applied biomedical research^37^,^38^. IDPs do not have stable, well-defined structures. Rather, their dynamic nature enables promiscuous yet specific binding to other cellular proteins. The importance of effecting change to an IDP structural ensemble is exemplified by a recent report on 4E-BP2, a functional repressor of translation initiation^39^. In this protein, the conformational ensemble is manipulated into a partially ordered state by phosphorylation. By analogy, the function of any IDP might be controlled using an appropriately derivatized, cell-penetrating tetraquinoline. Once inside the cell, the molecule would regulate the IDP by manipulating its structural transitions.

Oligoquinolines represent a design paradigm that specifically capitalizes on the plastic nature of IDPs. Rather than design a small molecule to complement the surface of a known protein structure, a compact and folded small-molecule structure is used to induce and/or stabilize a subset of peptide conformations. State-of-the-art docking approaches show that it is possible to meaningfully identify fragment moieties suitable for specific docking with computed or measured IDP substates. Recent approaches have targeted α-synuclein from Parkinson's^40^, Aβ from Alzheimer's^41^ and IAPP^42^. Such approaches are complementary to the presented work as the R-groups of oligoquinolines can be modified to include such identified moieties.

Naturally occurring folded proteins form specific interactions with biotic and abiotic targets in diverse contexts. Each fold represents a topology with tolerance to alterations in its solvent-exposed residues. The immunoglobulin fold alone has a theoretical surface diversity of 10^12^. The separation, albeit imperfect, of side-chain components relevant to folding from those responsible for molecular recognition contributes to this capacity. The oligoquinoline scaffold adopts a singular fold with as few as four subunits with complete separation of folding and molecular recognition determinants. The significance is that this is a class of compounds that as a result of an exploitable combination of structure and dynamics, can penetrate cellular membranes, transport cargo, and finally localize and perturb intracellular targets.

## Methods

### Materials

Thioflavin T (ThT) was purchased from Acros Organics (Fair Lawn, NJ, USA), lipids (dioleoylphosphatidylglycerol (DOPG) and dioleoylphosphatidylcholine (DOPC)) from Avanti Polar Lipids, Inc. (Alabaster, AL, USA), 96-well plates (black, w/flat bottom) from Greiner Bio-One (Monroe, NC, USA), silica plates (w/UV254, aluminium backed, 200 μm) and silica gel (standard grade, particle size=40–63 μm, 230 × 400 mesh) from Sorbent Technologies (Atlanta, GA, USA), dry solvents from Sigma Aldrich (St Louis, MI, USA) or VWR (Bridgeport, NJ, USA), 2,6-dichloro-3-nitropyridine, alkyl iodides, triethylamine (dry), 2-chloro-1-methylpyridinium iodide, *tert*-butyl bromoacetate, trifluoroacetic acid (TFA) and triethylsilane from Sigma Aldrich, and IAPP from Genscript (Piscataway, NJ, USA) and Elim Biopharmaceuticals (Hayward, CA, USA). IAPP was repurified and handled in-house as follows: IAPP (∼2 mg) was solubilized in 7 M guanidinium hydrochloride. The solution was filtered (0.2 μm) and transferred to C-18 spin column, washed twice with water (400 μl each), followed by 10% acetonitrile in water and 0.1% formic acid (v/v), and then eluted into 200 μl of 50% acetonitrile in water and 0.1% formic acid (v/v). The concentration of IAPP was calculated at 280 nm (*ɛ*=1,400 M^−1^ cm^−1^). The IAPP solution was divided into several aliquots (20–50 μl, 1–2 mM), lyophilized and stored as a white solid at −80 °C. A fresh stock solution of IAPP was prepared in water from lyophilized aliquots for each experiment. Alexa-594-labelled IAPP was prepared as described previously^10^.

### Unilameller vesicles

Unless otherwise stated, LUVs used in this work were prepared from a 1:1 mixture of DOPG/DOPC. The lyophilized mixture was hydrated in 100 mM KCl, 50 mM sodium phosphate and pH 7.4 for 20 min. A 20mg ml^−1^ solution of lipid in buffer was passed 21 times through a polycarbonate membrane (pore diameter=100 nm). The phospholipid content of the final material was measured using a total phosphorous assay^43^.

### Kinetic assay

Amyloid reactions were conducted in buffer containing liposome (630 μM lipid) and 20 μM ThT in a black 96-well plate. This was followed by the addition of small molecule dissolved in dimethylsulphoxide (DMSO; final DMSO concentration=0.5%, v/v). Fibre formation was initiated by the addition of IAPP stock solution. The final volume in each well was 200 μl. Kinetics of fibrillation was monitored by ThT fluorescence (excitation 450 nm and emission 485 nm), using a FluoDia T70 fluorescence plate reader (Photon Technology International, Edison, NJ, USA). The data were blank subtracted and renormalized to the maximum intensity of reactions containing only IAPP. Each kinetic trace was fit to a sigmoidal form:


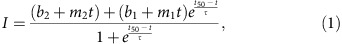


where *I* is the fluorescence intensity, *t* is time, and *b*, *m* and *τ* are dependent fitting variables. All samples were run at least in triplicate and error bars shown in the text represent ±1 s.d.

### Transmission electron microscopy

IAPP (10 μM) was incubated in buffer (100 mM KCl, 50 mM sodium phosphate, pH 7.4) with 630 μM LUVs in the presence and absence of 1:1 IAPP:small molecule. Aliquots were incubated for 60 s on glow-discharged (25 mA and 30 s) carbon-coated 300-mesh copper grids. After drying, grids were negatively stained for 60 s with uranyl acetate (2%, w/v). Micrographs were taken on a Phillips Tecnai 12 transmission electron microscope (Hillsboro, Oregon, USA) at 120 kV accelerating voltages. All conclusions drawn from images in this work include at least one repeat in which the sample identity was withheld from the investigator preparing and analysing images.

### Isothermal calorimetry titration

ITC experiments were conducted in a NANO-ITC (TA instruments, New Castle, DE, USA). Solutions of small molecules (100 μM in 1 mM KCl, 20 mM Tris, pH 7.4) was serially titrated (50 μl injections via rotary syringe) into an isothermal sample cell containing 10 μM IAPP (250 μl in 1 mM KCl, 20 mM Tris, pH 7.4) with a stirring speed of 300 r.p.m. Ten-second injections were spread 240 s apart. The heat associated with each injection (oligoquinoline:IAPP) was extracted by integrating area under each curve using NanoAnalyze software (New Castle, DE, USA). The heat of small molecule binding to IAPP was corrected by subtracting heat of oligoquinoline injections into buffer. The final corrected heats were plotted as a function of molar ratio (oligoquinoline:IAPP) and fitted with a one binding site model.

### Circular dichroism

CD spectra were collected on AVIV MODEL 215 (AVIV Instruments, Inc., Lakewood, NJ, USA) for 25 μM IAPP mixed with 1200 μM LUVs in 100 mM KCl and 50 mM sodium phosphate at pH 7.4. The data were collected from 200 to 260 nm at 0.5-nm intervals with 10 s averaging time and an average of four repeats. The CD spectra in the presence of oligoquinolines were recorded as above except collecting from 200 to 500 nm at a stoichiometric ratio of 1:1 (oligoquinoline:IAPP).

### Synthesis of ADM-116

To a solution of compound **9** (88 mg, 0.1 mmol, Supplementary Fig. 60) in ethylacetate (10 ml), Pd/C (15 mg) was added and the reaction mixture was started with constant stirring in the atmosphere of H_2_ (g, 1 atm.) at room temperature. The progress of the reaction was monitored using thin-layer chromatography. The disappearance of the starting material confirms the completion of the reaction (10 h). The reaction mixture was filtered and the filtrate was dried over rotovap to afford the desired product as a yellow solid, which is used in next step without further characterization.

To a solution of the reduced analogue of compound **9** (51 mg, 0.06 mmol, Supplementary Fig. 60) in dichloromethane (10 ml), triethylamine (20 mg, 0.2 mmol, anhydrous) and 2-chloromethyl-1-methyl pyridinium iodide (15 mg, 0.06 mmol) were added and the reaction was refluxed for 20 min at 50 ^°^C under inert atmosphere. To this solution, compound **3** (17 mg, 0.05 mmol, Supplementary Fig. 59) was added and reaction started with constant stirring at 50 °C under inert atmosphere. The reaction mixture was stirred for 12 h after which the volatiles were removed on rotovap. Flash chromatography (0–35% ethylacetate in hexane, v/v) yielded the desired product (compound **10**) as a yellow solid (54 mg, 83%).

To a solution of compound **10** (44 mg, 0.04 mmol) in dichloromethane (2 ml), triethylsilane (0.1 ml, excess) and trifluoroacetic acid (0.5 ml, excess) were added and the reaction solution was stirred at room temperature for 4 h. The reaction mixture was dried and washed with cold diethyl ether (4 × 3 ml) that resulted in a light brown solid as compound **ADM**-**116** (35 mg, 89%).

### Synthesis of ADM-116_*F*_

To a round bottom flask *tert*-butyl ADM-116 (30 mg, 0.027 mmol; Supplementary Fig. 63) was added, followed by the addition of ethylene diamine (2 ml, excess) and 4-dimethylaminopyridine (catalytic amount). The reaction mixture was stirred overnight at 50 °C under inert atmosphere. The reaction mixture was then poured into water (10 ml) that resulted in a white precipitate. The precipitate was dissolved in ethylacetate (10 ml) and washed with 0.1 N HCl (3 × 10 ml) and then dried over Na_2_SO_4_. The compound was then dissolved in dimethylformamide (3 ml, anhydrous), followed by the addition of 4-dimethylaminopyridine (0.25 ml, excess) and fluorescein-5-isothiocyanate (12 mg, 0.03 mmol). The reaction ran overnight in dark with constant stirring under inert atmosphere. The solvents were evaporated on rotovap. The orange solid was dissolved in dichloromethane (2 ml), followed by the addition of triethylsilane (0.1 ml, excess) and trifluoroacetic acid (0.4 ml, excess). The solution was stirred in dark for 4 h and then dried on rotovap. The product was washed with cold diethyl ether (3 × 3 ml). The reaction mixture was loaded on a semipreparation reverse-phase high-performance liquid chromatography (Varian ProStar with VYDAC reverse-phase C-18 column) to purify the product. The desired product (**ADM-116**_***F***_) was obtained as an orange solid (3.5 mg, 25%, overall yield for three steps).

### Synthesis of ADM-3

Synthesis and characterization of ADM-3 are reported elsewhere^20^.

### Synthesis of ADM-3_*F*_

To a solution of *tert*-butyl ADM-3-NCS (20 mg, 0.027 mmol, Supplementary Fig. 64) in pyridine (5 ml, anhydrous), *N*, *N*-diisopropylethylamine (6 μl, 0.06 mmol) was added and the solution was stirred for 10 min. To this solution, 5-(aminoacetamido) fluorescein (22 mg, 0.054 mmol) was added and the reaction was started in dark with continuous stirring under inert atmosphere. The reaction solution was stirred overnight in dark. The product was purified using column chromatography (0–20% methanol in dichloromethane with 0.5 % triethylamine, v/v) as an orange solid (23 mg, 76%).

To a solution of *tert*-butyl ADM-3_*F*_ (20 mg, 0.017 mmol, Supplementary Fig. 64) in dichloromethane (4 ml), triethylsilane (0.1 ml, excess) was added, followed by the addition of trifluoroacetic acid (0.4 ml, excess) and the reaction solution was stirred in dark at room temperature for 4 h. The solution was then dried and the orange solid was washed with cold diethyl ether (3 × 5 ml), which afforded the desired product (**ADM-3**_***F***_) as an orange solid (13 mg, 72%).

### Small-molecule characterization

Final steps of small-molecule purification was conducted by high-performance liquid chromatography using a Varian ProStar with VYDAC reverse-phase columns (4.6 × 100 mm, 1 ml min^−1^, analytical; 10 × 100 mm, 3 ml min^−1^, semi-preparation; Supplementary Fig. 12–58; Supplementary Methods). The mobile phase was composed of A: 5% ACN; 95% H_2_O; and 0.1% TFA(v/v), and B: 95% ACN; 5% H_2_O; and 0.08% TFA (v/v). The solution NMR spectra of small molecules were recorded on 400, 500 and 600 MHz Agilent spectrometers. The deuterated solvents used for O-*tert*-butyl ester protected and deprotected (O–COOH) oligoquinolines were CDCl_3_ and (CD_3_)_2_SO, respectively. Splitting patterns that were difficult to interpret are indicated as multiplet (m) or broad (b). Mass spectra were obtained using either MALDI-TOF Voyager DE Pro (Yale University, CBIC centre) or University of Illinois Mass Spectrometry Facility. High-resolution electrospray ionization mass spectra were obtained using the Waters Synapt G2-Si ESI MS mass spectrometer (Milford, MA, USA).

### Fluorescence correlation spectroscopy

Fluorescence correlation spectroscopy measurements were made on a laboratory-built instrument based around an inverted microscope using an Olympus IX71 microscope (Olmpus, Tokyo, Japan), as described previously^44^. In brief, a continuous-emission 488-nm diode-pumped solid-state 50-mW laser was set to 5–20-mW output power and further adjusted with neutral density filters to 18 μW of power just before entering the microscope. Fluorescence was collected through the objective and separated from the excitation laser using a Z488rdc long-pass dichroic and an HQ600/200m band-pass filter (Chroma, Bellows Falls, VT, USA). Fluorescence was focused onto the aperture of a 50-μm optical fibre coupled with an avalanche photodiode (Perkin Elmer, Waltham, MA, USA). A digital correlator (Flex03LQ-12; Correlator.com, Bridgewater, NJ, USA) was used to generate autocorrelation curves.

Measurements were made in eight-well chambered coverglasses (Nunc, Rochester, NY, USA) that were plasma treated followed by precoating with polylysine-conjugated polyethylene glycol (PEG-PLL) to prevent ADM-116 and/or IAPP from adsorbing to chamber surfaces. Low-density PEG coating was performed by preparing a 100-mg ml^−1^ solution of PEG (MW=2 kDa, NANOCS, Boston, MA, USA) in poly-L-lysine hydrobromide (Sigma Aldrich). Reaction was performed for 6 h in dark at room temperature, followed by overnight dialysis. Chambers were incubated overnight with PEG-PLL solution, rinsed thoroughly with Millipore water and stored in water before use. All samples were incubated in buffer (20 mM Tris, pH 7.4, 10 mM NaCl) for 1 h before taking measurements. Titration was performed keeping constant concentrations of ADM-116_*F*_ (25 nM) and adding increasing concentrations of IAPP. The autocorrelation curves were collected at regular intervals (10 min), and each autocorrelation curve was collected over 10 s and repeated 30 times.

Autocorrelation curves were fitted using Matlab (The MathWorks, Nattick, MA, USA). Global analyses were performed on IgorPro (Wavemetrics, Portland, OR, USA). For ADM-116, the model for a single diffusing species undergoing three-dimensional Brownian diffusion with a triplet state is given by equation (2) (ref. 45).


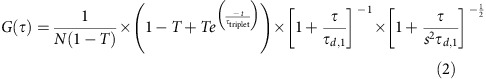


Here *N* is the number of ADM-116_*F*_ molecules in the detection volume, *T* is the fraction of molecules in the triplet state and *τ*_triplet_ is the triplet-state relaxation time. The characteristic translational diffusion time of a diffusing particle is given by *τ*_*d*,1_.

In the presence of IAPP, the model for two-component analysis is given by equation (3) (ref. 45).


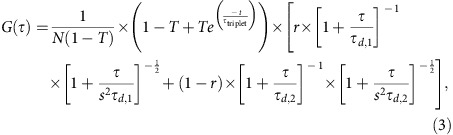


where *r* is the fraction of the fast-diffusing component with a diffusion time τ_*d*,1_, whereas τ_*d*,2_ is the diffusion time of the slow component. The structure factor, *s*, was determined as a free parameter for solutions of free Alexa Fluor 488 hydrazide dye and then fixed to the experimentally determined value of 0.17 for all subsequent fittings. For experiments in the presence of IAPP, global analysis was performed by fixing the predetermined values for the diffusion coefficient, triplet diffusion time and amplitude for ADM-116. The triplet state of IAPP-bound and -unbound ADM-116 was considered to be the same.

### Confocal microscopy

Images were obtained in eight-well NUNC chambers (Thermo Scientific, Rochester, NY, USA) seeded with 20,000–25,000 cells per well. After culturing for 48 h, the medium was replaced with the medium containing constituents according to the experiment performed. For time-dependent localization experiments of IAPP, the medium contained 100 nM IAPP_A594_, 13 μM unlabelled peptide and incubated for the specified time points. For experiments in the presence of ADM-116_*F*_ and ADM-3_*F*_, additional fluorescein-labelled and -unlabelled small molecules, 200 and 13 μM, respectively, was introduced in the medium. For delayed addition experiment with small molecules, the medium was removed for the second time, replacing with medium containing the small molecule. Images were acquired after 48-h total incubation time. Imaging was carried out at the Yale Department of Molecular, Cellular and Developmental Biology imaging facility on a Zeiss LSM 510 confocal microscope, using a × 63 Plan-Apo/1.4-NA oil-immersion objective with DIC capability (Carl Zeiss, Oberkochen, Germany). For all experiments reporting on the uptake of labelled IAPP, the gain setting for the red channel was kept constant from sample to sample. Image acquisition and processing were achieved using Zeiss Efficient Navigation and Image J software.

### Imaging FRET

The INS-1 growth media was then replaced with media containing 100 nM IAPP_A594_ and incubated for 18 h. Media was then replaced a second time with media containing 200 nM ADM-116_*F*_. Images were taken after 5-h incubation. Background FRET was determined using parallel experiments where 100 nM IAPP_A594_ was initially incubated for 18 h in cells in the presence of a further 13 μM of unlabelled IAPP. Media was then replaced with media containing 200 nM ADM-116_*F*_. Imaging was carried out at the Yale Department of Molecular, Cellular and Developmental Biology imaging facility on a Zeiss LSM 510 confocal microscope, using a × 100 Plan-Apo/1.4-NA oil-immersion objective with DIC capability (Carl Zeiss). For ADM-116_*F*_, fluorescein was excited with a 488-nm Argon2 laser and detected through a 505–550-nm emission filter. IAPP_A594_ was excited with a 561-nm Argon2 laser and detected through a 590–630-nm emission filter. For all experiments, the pinhole was kept constant to the *Z*-slick thickness of each filter channel. Single-cell images were obtained for donor alone, acceptor alone and donor–acceptor fusion channels. Image acquisition and processing were achieved using Zeiss Efficient Navigation and Image J software^46^. The Image J plugin, RiFRET^44^, was used to calculate and remove the bleed through for each channel and to calculate a pixel-based FRET efficiency. The FRET distance was then calculated using:


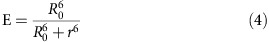


where *E* is the calculated efficiency of FRET energy transfer, *R*_0_ is the Förster distance (60 Å for fluorescein–Alexa-594 pair) and *r* is the distance between the donor and the acceptor.

### Cell culture

Rat insulinoma INS-1 cells (832/13, Dr Gary W. Cline, Department of Internal Medicine, Yale University) were cultured at 37 °C and 5% CO_2_ in phenol red-free RPMI 1640 media supplemented with 10% fetal bovine serum, 1% penicillin/streptomycin (Life Technologies, Carlsbad, CA, USA) and 2% INS-1 stock solution (500 mM HEPES, 100 mM L-glutamine, 100 mM sodium pyruvate and 2.5 mM β-mercaptoethanol). Cells were passaged on reaching ∼95% confluence (0.25% Trypsin-EDTA, Life Technologies), propagated and/or used in experiments. Cells used in experiments were pelleted and resuspended in fresh media with no Trypsin-EDTA. COS-1 cells (from immortalized African green monkey kidney; CRL-1650; American Type Culture Collection) were cultured at 37 °C in 5% CO_2_ in DMEM (Life Technologies) supplemented with 10% fetal bovine serum and 1% penicillin/streptomycin (Life Technologies). Once the cells reached ∼95% confluence, they were passaged (using 0.25% Trypsin-EDTA; Life Technologies) into fractions and propagated or used in experiments.

### Giant plasma membrane vesicle isolation

GPMVs were isolated from INS-1 cells as previously described^12^,^34^. In brief, cells were plated in 35-mm dishes and cultured for 48 h. Cells were washed with a 10 mM HEPES, 150 mM NaCl, 2 mM CaCl_2_ (pH=7.4) twice and were then exposed to 10 mM *N*-ethyl maleimide (Sigma Aldrich) for 2 h. Collected samples were then passed over a gravity-flow column (Bio-Rad) containing size-exclusion Sephacryl matrix of pore size 400-HR (Sigma Aldrich), allowing the purification of GPMVs from residual cell debris.

### GPMV imaging

Images were obtained in eight-well NUNC chambers (Thermo Scientific) including 250 μl of GMPV stock solution. For experiments in the presence of ADM-116_*F*_ or ADM-3_*F*_, 200 nM of small molecule was incubated in the GMPV solution for 24 h at room temperature. Imaging was carried out at the Yale Department of Molecular, Cellular and Developmental Biology imaging facility on a Zeiss LSM 510 confocal microscope, using a × 63 Plan-Apo/1.4-NA oil-immersion objective with DIC capability (Carl Zeiss). For all experiments, the gain setting for the green channel was kept constant from sample to sample. Image acquisition and processing were achieved using Zeiss Efficient Navigation and Image J software^46^.

### Cell viability

Cell viability was measured colourimetrically using the CellTiter Blue (CTB, Promega, Madison, WI, USA) fluorescence-based assay. Cells were plated at a density 5,000 cells per well in 96-well plates (BD Biosciences, San Diego, CA). Peptide was directly introduced to each well after 48 h of culture and then further incubated for an additional 48 h. For time-dependent experiments, cells were incubated with peptide for the specified time points. After the incubation period, 20-μl CellTiter Blue reagent was added to each well and incubated at 37 °C and 5% CO_2_ for 2.5–3.5 h. Fluorescence of the resorufin product was measured on a FluoDia T70 fluorescence plate reader (Photon Technology International, Birmingham, NJ, USA). All wells included the same amount of water to account for different concentrations of peptide added to sample wells. Wells that included water vehicle but not peptide served as the negative control (0% toxic), and wells containing 10% DMSO were the positive control (100% toxic). Per cent toxicity was calculated using the following equation:


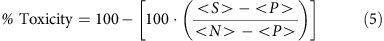


Each independent variable is the average of eight plate replicates from the negative control (<*N>*), positive control (<*P*>) and samples (<*S*>). Results presented for viability experiments are an average of three such experiments conducted independently on different days. Error bars represent the s.e.m.

Apoptosis was measured colourimetrically by detecting caspase-3/7 (Caspase-Glo 3/7 Assay, Promega). Cells were plated at a density 5,000 cells per well in 96-well plates (BD Biosciences). Peptide was directly introduced to each well after 48 h of culture and then further incubated for the times specified in the main text. After the incubation period, 20-μl Caspase-Glo 3/7 reagent (containing a mixture of caspase-3/7 DEVD-aminoluciferin substrate and a proprietary thermostable luciferase in a reagent optimized for caspase-3/7 activity) was added to each well, and incubated at 37 °C and 5% CO_2_ for 2 h. Fluorescence of the free aminoluciferin product was measured on a FluoDia T70 fluorescence plate reader (Photon Technology International). For protein concentration dependence measurements, carrier buffer was added as required to ensure identical volumes of protein were added to each well.

## Additional information

**How to cite this article:** Kumar, S. *et al*. Foldamer-mediated manipulation of a pre-amyloid toxin. *Nat. Commun.* 7:11412 doi: 10.1038/ncomms11412 (2016).

## Supplementary Material

Supplementary Information Supplementary Figures 1-65, Supplementary Tables 1-2, Supplementary Methods and Supplementary References.
