# Supplementary material for: Foldamer-mediated manipulation of a pre-amyloid toxin
Source: Nat Commun. 2016 Apr 25;7:11412. doi: 10.1038/ncomms11412 (PMC4848510; doi:10.1038/ncomms11412)
Supplement: Supplementary Information — Supplementary Figures 1-65, Supplementary Tables 1-2, Supplementary Methods and Supplementary References. [file ncomms11412-s1.pdf]

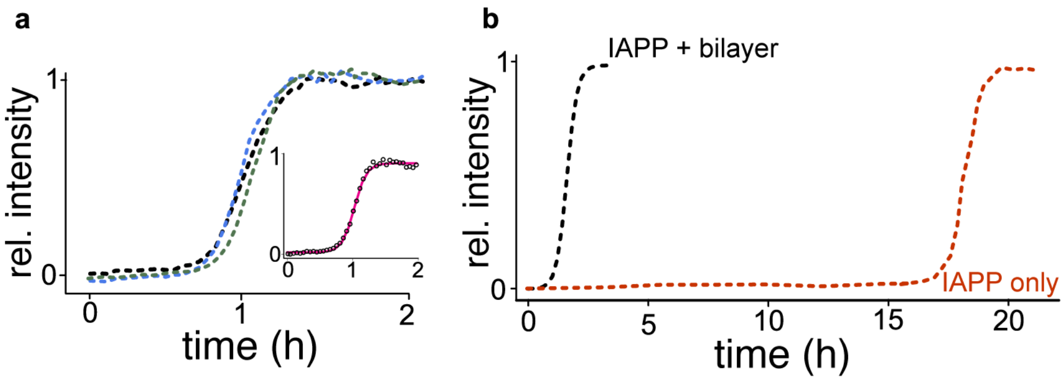

Supplementary Figure 1: Kinetic profiles of IAPP self-assembly. a, Three representative kinetic profiles for a standard reaction of 10 µM IAPP catalysed by the presence of 630 µM LUVs (DOPG:DOPC, 1:1, 100 nm) **(inset)** A representative sigmoid fit (magenta) used to extract reaction midpoints, t_{50}. **b,** Representative comparison of the kinetic profiles of (10 µM) IAPP fibrillation in the absence (black) and presence (red) of 630 µM LUVs. All experiments were conducted at least in triplicate with errors in the main text reported as ± one standard deviation.

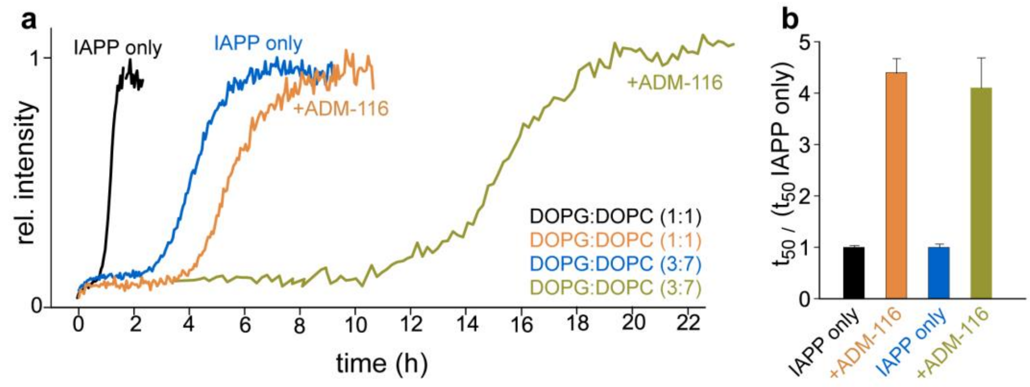

Supplementary Figure 2: Effect of lipid headgroup anionic content on the catalysis of IAPP self-assembly. Representative (**a**) and statistics (**b**) of reaction midpoints, t_{50}, for 10 μM IAPP fibre formation reactions catalysed by extruded unilamellar vesicles (100 nm, 630 μM monomer units, with DOPG:DOPC at the indicated ratio. Also shown are repeated reactions preformed in the presence of 1 μM ADM-116. All experiments were conducted in triplicate with errors reported as ± one standard deviation.

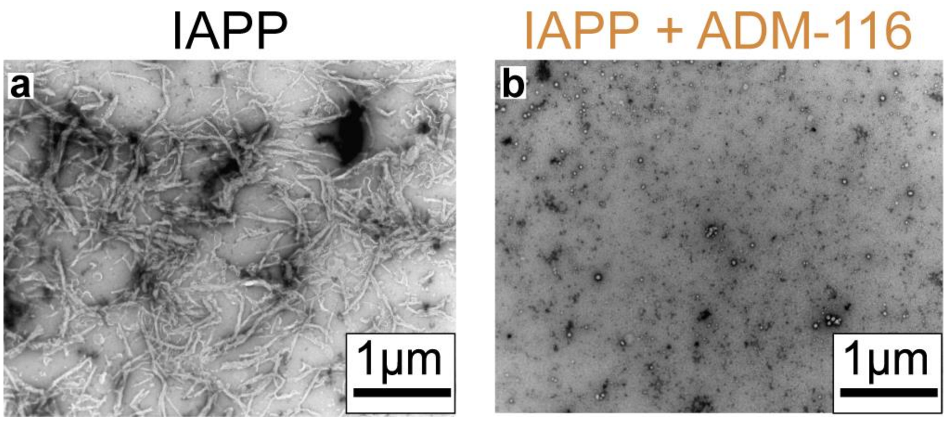

Supplementary Figure 3: Imaging of ADM-116 inhibited assembly. Negative stain TEM images of liposome catalysed IAPP (10 μM) self-assembly in the absence, **a,** and presence, **b,** of 10 μM ADM-116.

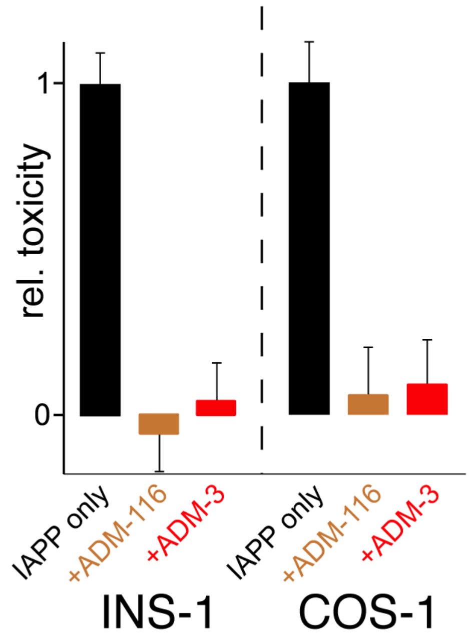

Supplementary Figure 4: Comparison of INS-1 and COS-1 rescue by ADM-116 and ADM-3. Statistics of toxic effect of IAPP applied to INS-1 cells or COS-1 cells and measured 48 h later using CTB. Data is shown for IAPP alone and with equimolar ratio of the indicated small molecule co-added at a 1:1 ratio. Data is renormalised to the toxicity induced only by IAPP. Error bars are standard deviations from three sets of experiments conducted on separate occasions with eight technical repeats.

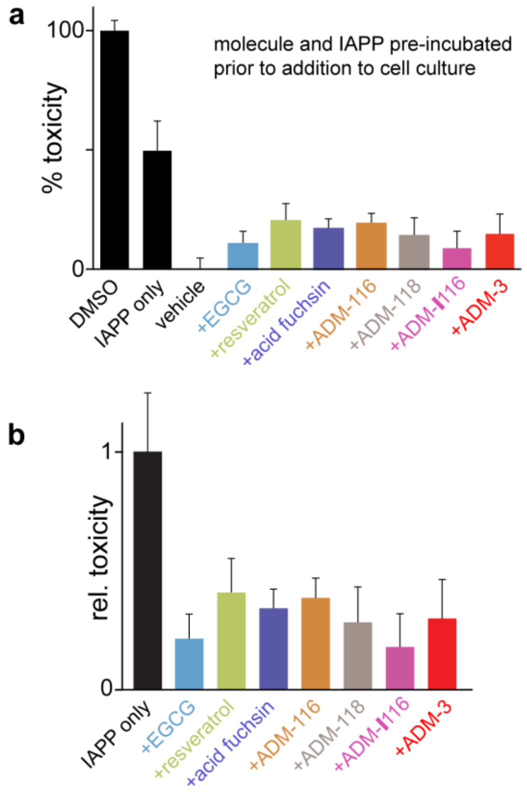

Supplementary Figure 5: Rescue of INS-1 cells from IAPP induced toxicity. Statistics of toxic effect of 13 µM IAPP applied at time-zero to INS-1 cells and measured 48 h later using CTB. Data is shown for IAPP alone and with equimolar mixtures of the indicated small molecule. Small molecule:IAPP complexes were permitted to form over a period of 14 h prior to adding to cells. In **(a)** data is presented as % toxicity where the lower baseline is the CTB response measured by addition of IAPP-free buffer (vehicle). For the upper baseline, DMSO at 10% is used as it is wholly toxic to INS-1 cells. CTB response at this condition sets the value of 100% toxicity. Note that in other publications, analogous data is sometimes expressed as viability (100% - %toxicity). In **(b)**, the data in **(a)** is shown renormalised to the toxicity induced only by IAPP. Error bars are standard deviations from three sets of experiments conducted on separate occasions with eight technical repeats per set (n=24).

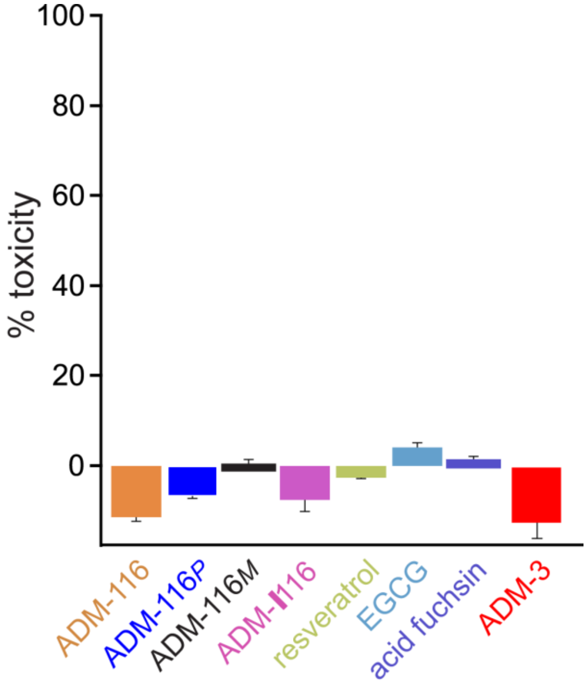

Supplementary Figure 6: Assessment of effect of small molecules on cell viability. INS-1 cells were incubated with 13 µM of each of the indicated small molecules in a manner matched to that used for work shown in the main text (Fig. 4a). Viability was assayed after 48 h using CTB and compared to carrier-only controls. Error bars represent the standard deviation in the mean of four replicates.

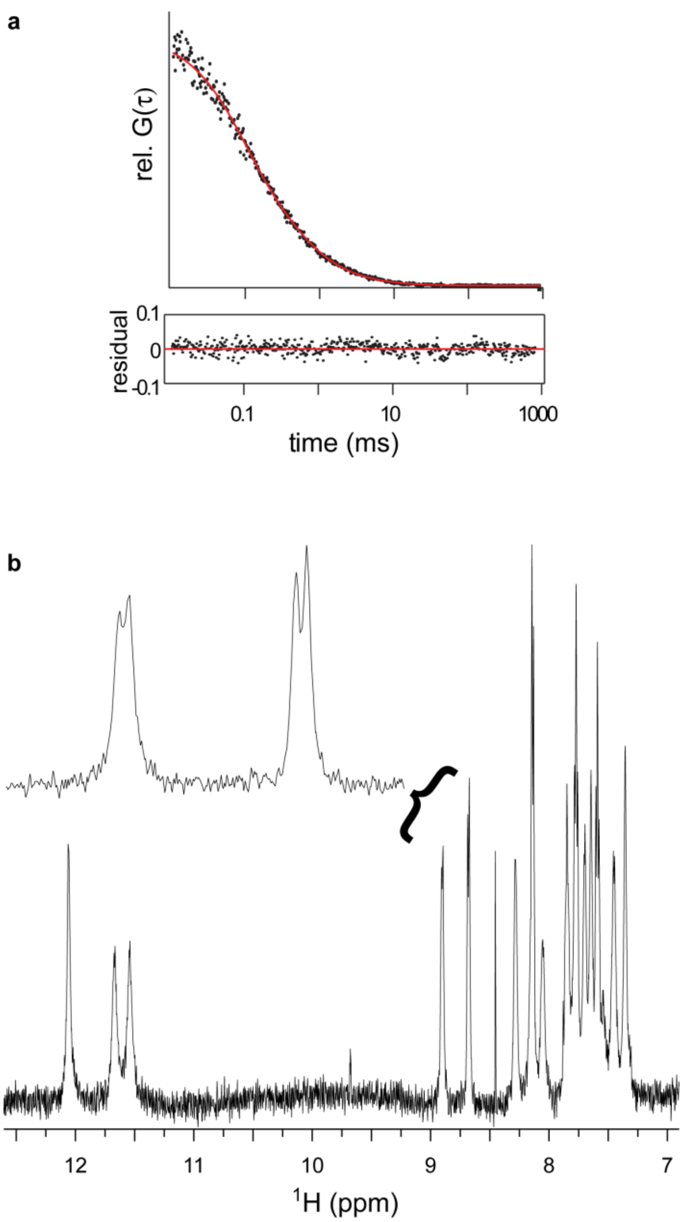

Supplementary Figure 7: Solubility of ADM-116 in aqueous buffer. **a,** Diffusion of 13 μM ADM-116 with 25 nM ADM-116$_F$ measured by FCS in 20 mM Tris, pH 7.4, 10 mM NaCl. A fit to a function that includes only a single diffusible species (τ_D =100 ± 20 μs) is shown (red, see Methods). Residuals of the fit are shown below the main data. **b,** ^{1}H NMR spectrum of the aromatic and amide region of ADM-116. Conditions were 20 μM ADM-116 in 50 mM phosphate buffer, pH 7.4, 100 mM KCl. NMR spectrum was recorded on an Agilent DD2 NMR spectrometer at 600 MHz.

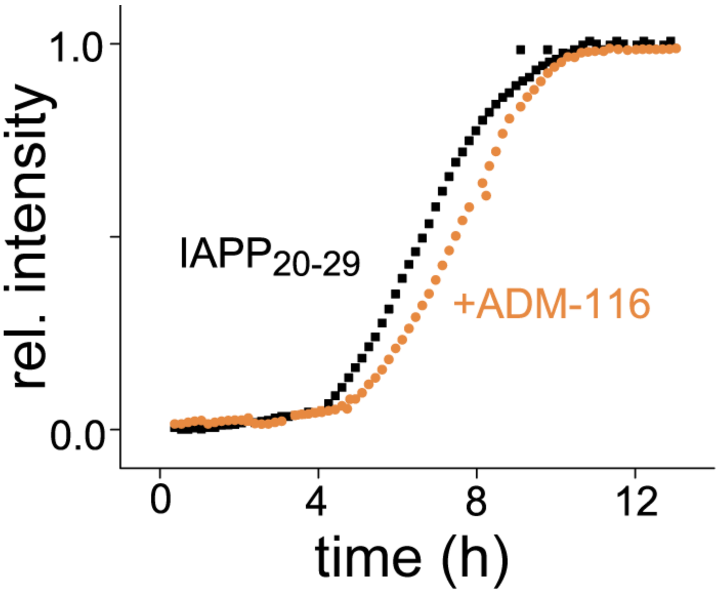

Supplementary Figure 8: Effect of ADM-116 on the assembly kinetics of IAPP$_{20\text{-}29}$. Representative kinetic profile of amyloid assembly by 200 µM of the 10-residue peptide, IAPP$_{20\text{-}29}$. Reactions are shown in the absence and presence of 200 µM ADM-116 at equimolar ratio.

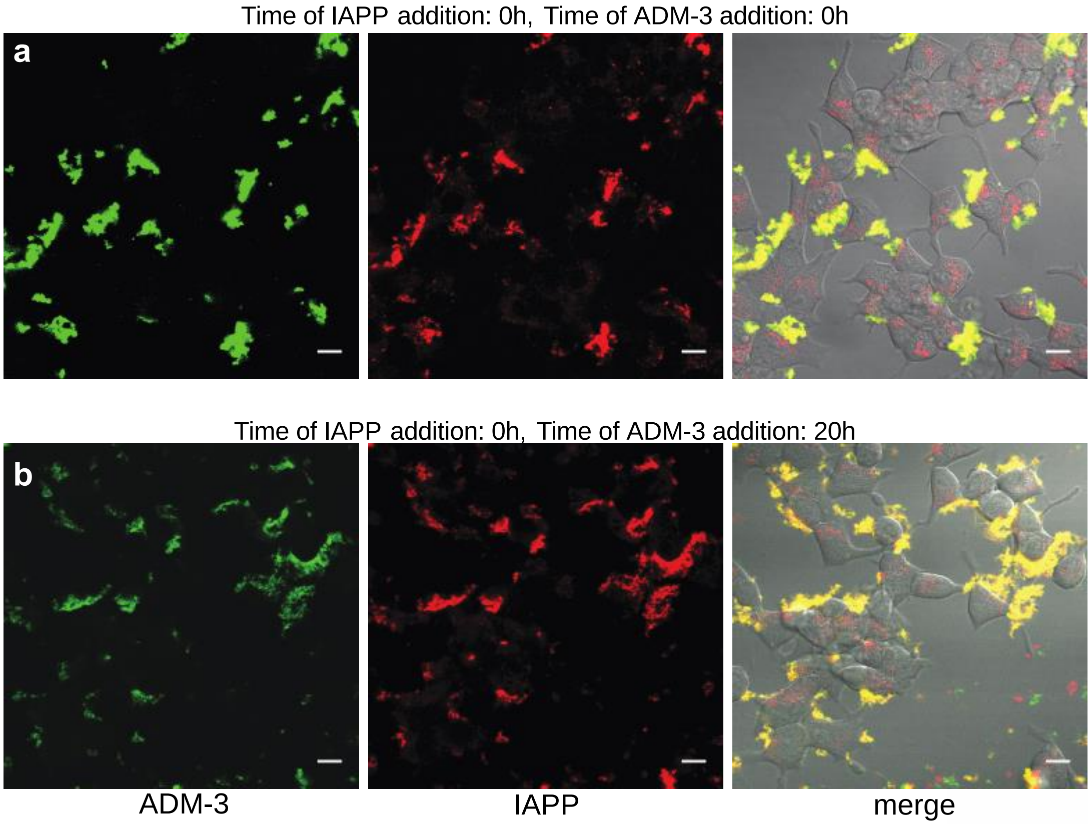

Supplementary Figure 9: Colocalisation of IAPP and ADM-3. INS-1 cells were exposed to a rescued condition (13 μM IAPP and 15 μM ADM-3) doped with 200 nM ADM-3$_F$ and 100 nM IAPP$_{A594}$). **a,** IAPP and small molecule were added at the same time to culture media. **b,** ADM-3 was added 20 h after addition of IAPP.

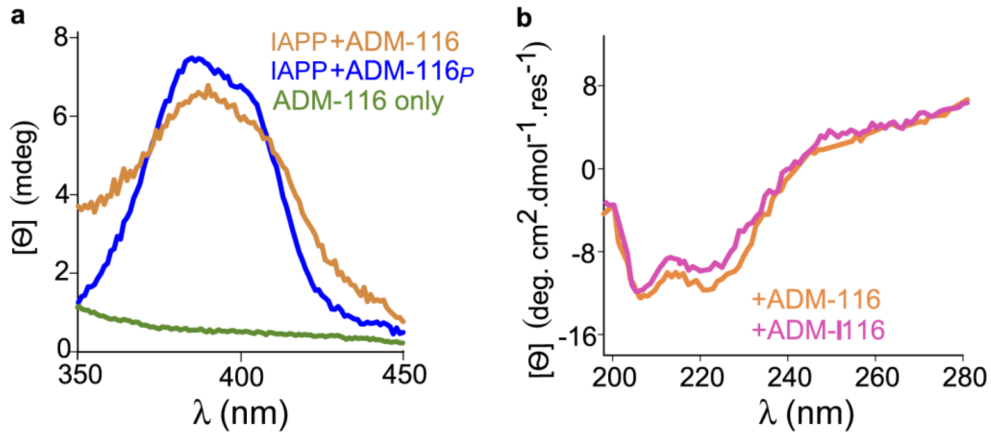

Supplementary Figure 10: ADM-116 induced conformational changes. a, Visible CD spectra of 25 μM ADM-116 or ADM-116*P* in the presence of liposome (630 μM in monomer units, DOPG:DOPC, 1:1, 100 nm) and 25 μM IAPP. **b,** Far-UV CD spectra of 25 μM IAPP in the presence of ADM-116 or ADM-**I**116 at a ratio of 1:0.5 (IAPP:small molecule).

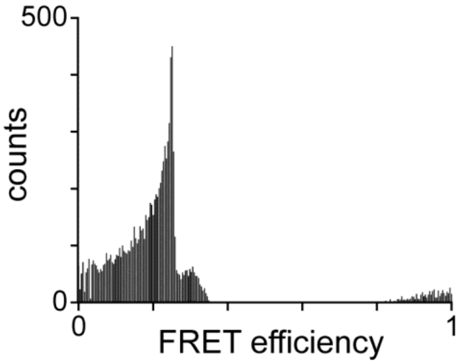

Supplementary Figure 11: Determination of background FRET in confocal imaging. Confocal imaging and processing for FRET was conducted in a manner matched to that used in the main text (Fig. 7**c,d**). Preparation of the cells was also matched except that the 100 nM $IAPP_{594}$ was augmented with 13 μM unlabelled IAPP to dilute out any possibility of structure-specific FRET taking place.

Supplementary Figures 12-58 (NMR spectra, MALDI-TOF, ESI, and HPLC traces)

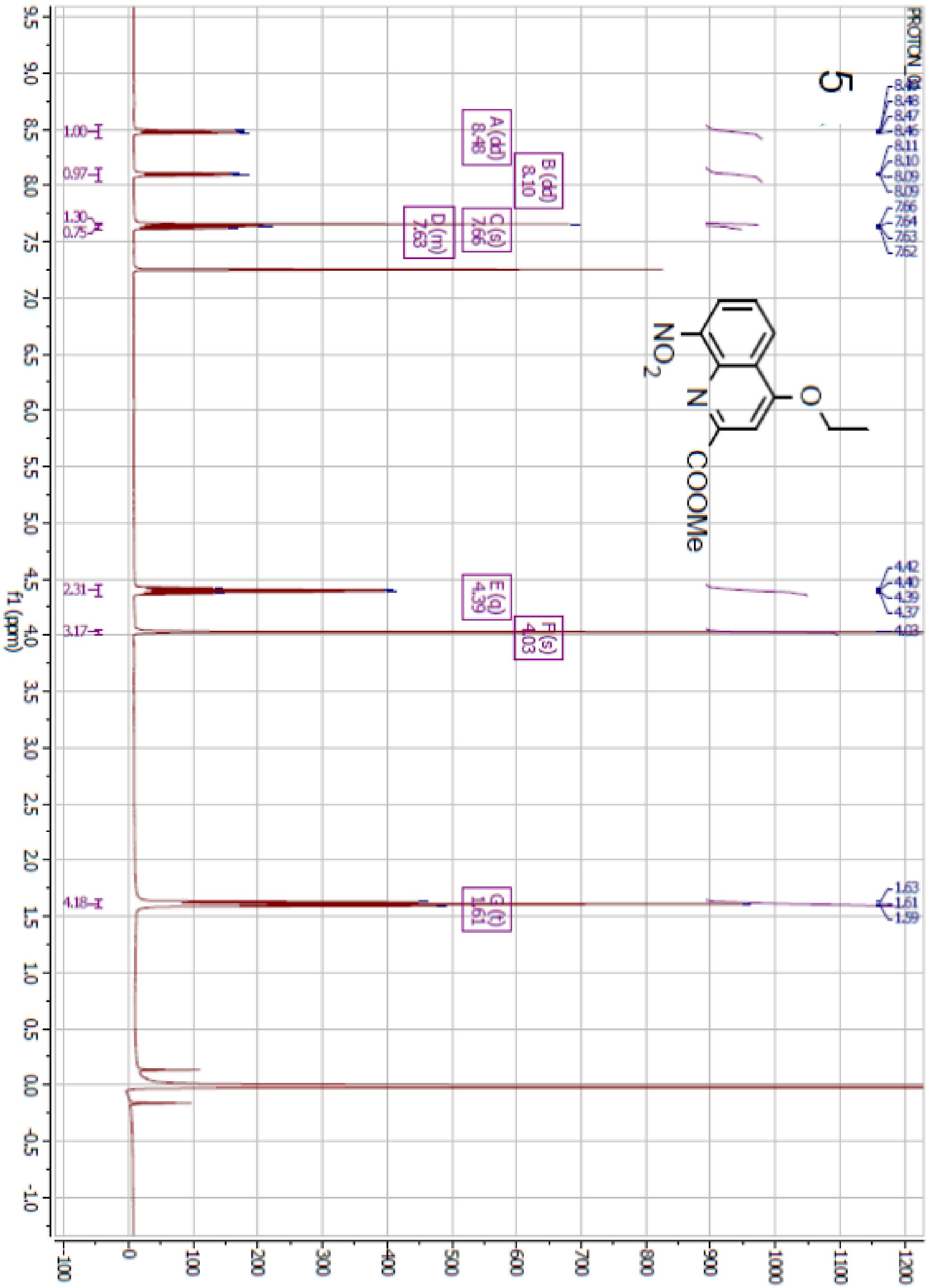

Supplementary Figure 12. ^{1}H NMR of 5.

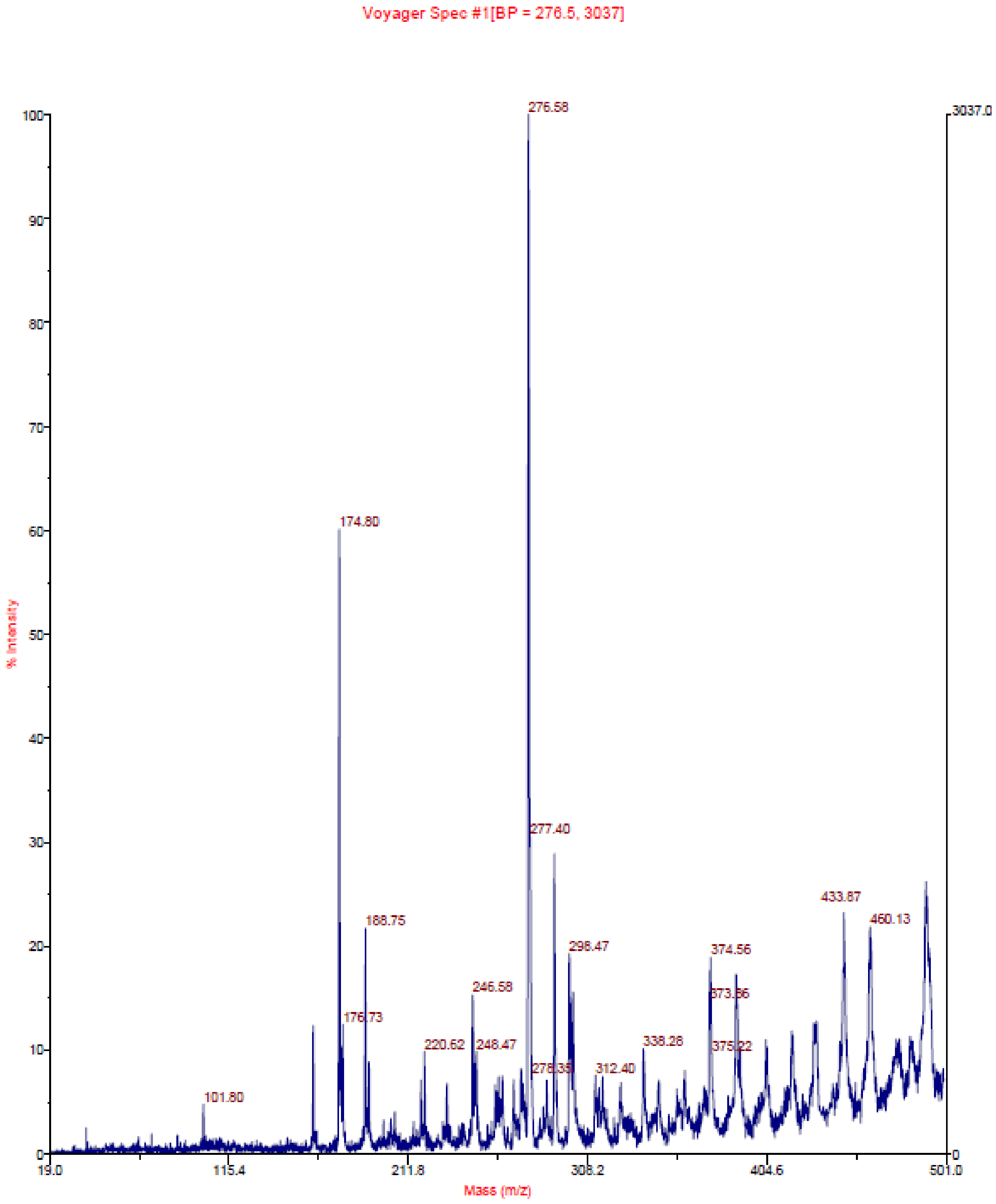

Supplementary Figure 13. MALDI-TOF of 5.

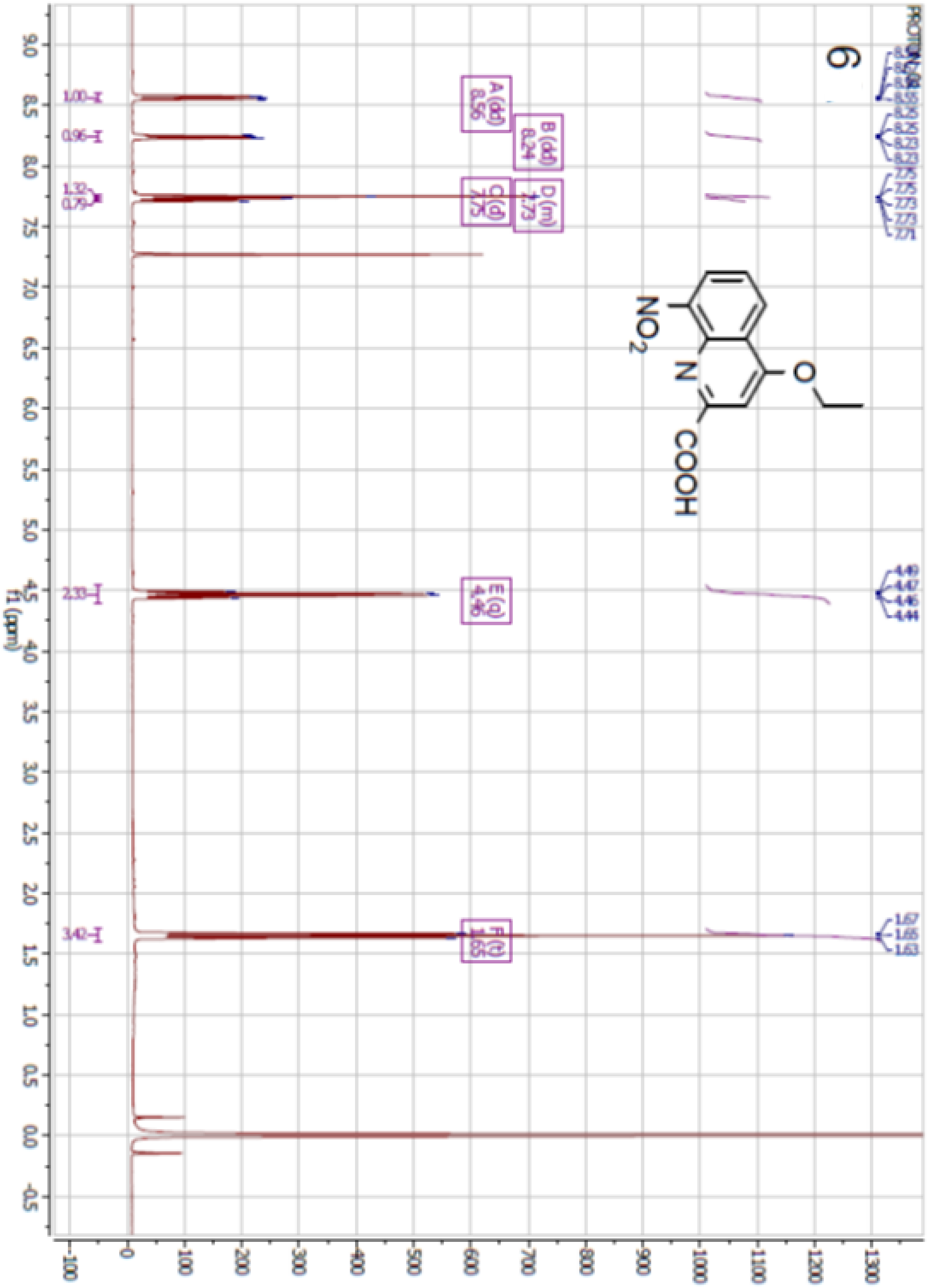

Supplementary Figure 14. ^{1}H NMR of 6.

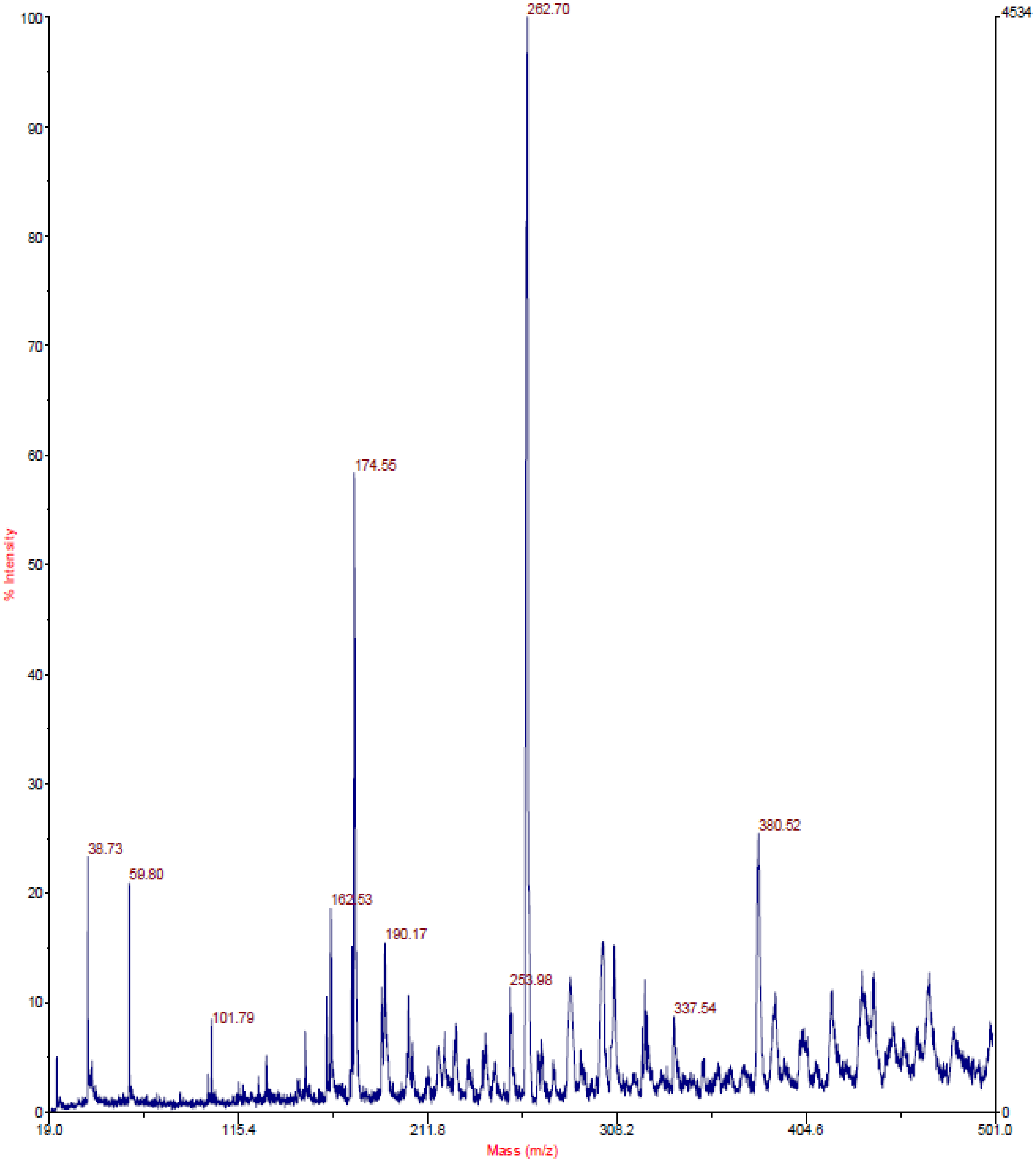

Supplementary Figure 15. MALDI-TOF of 6.

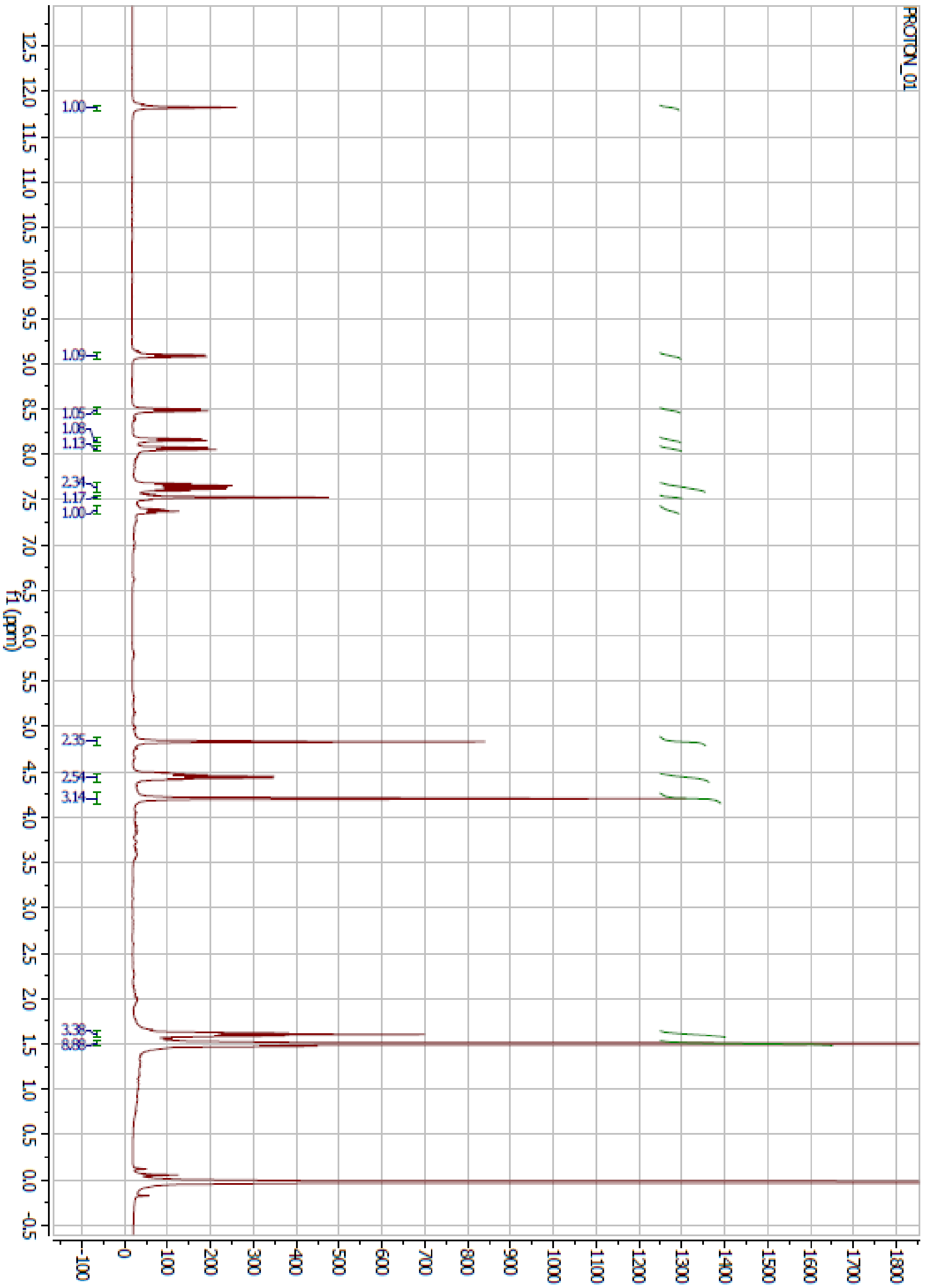

Supplementary Figure 16. ^{1}H-NMR of 8.

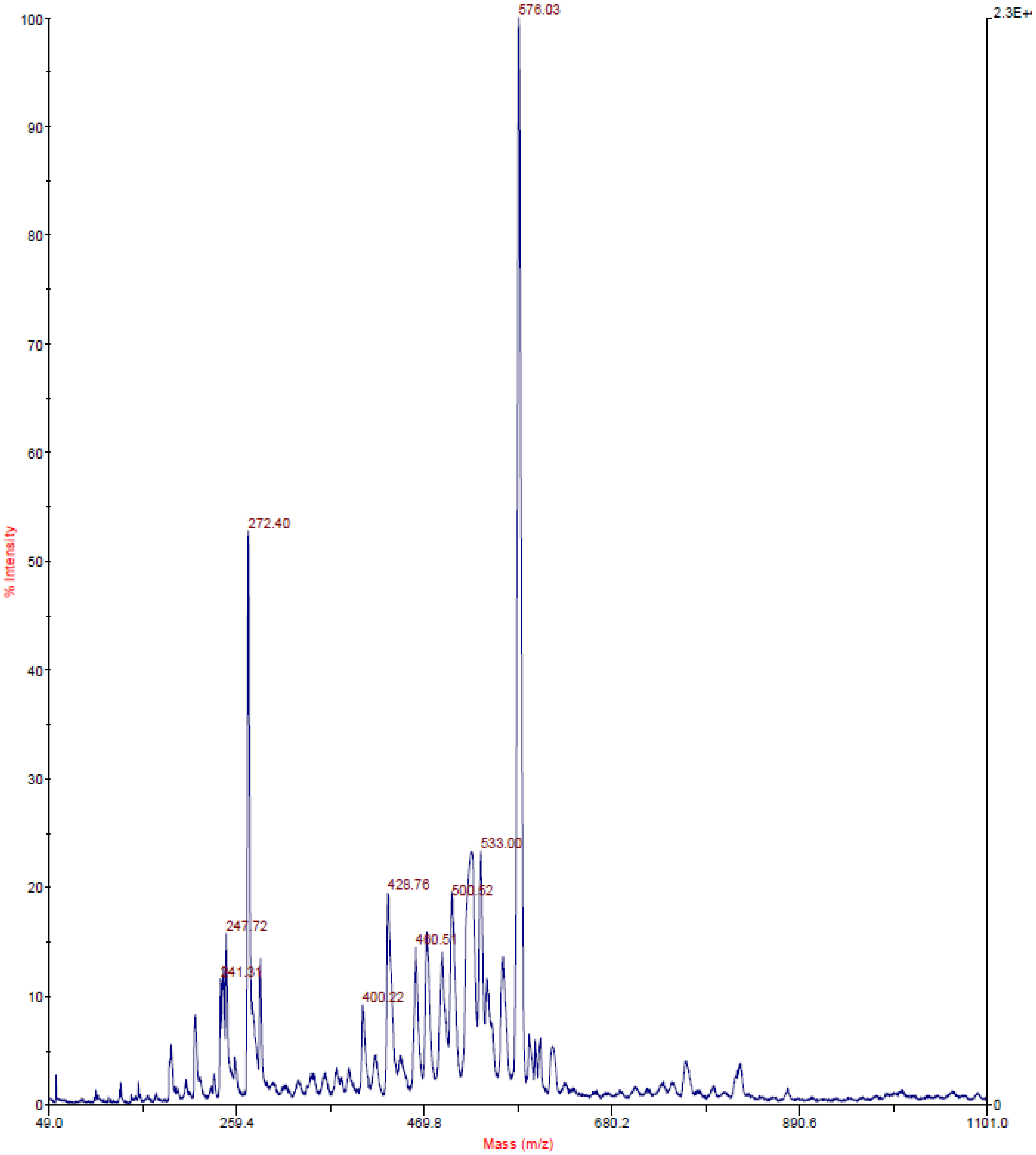

Supplementary Figure 17. MALDI-TOF of 8.

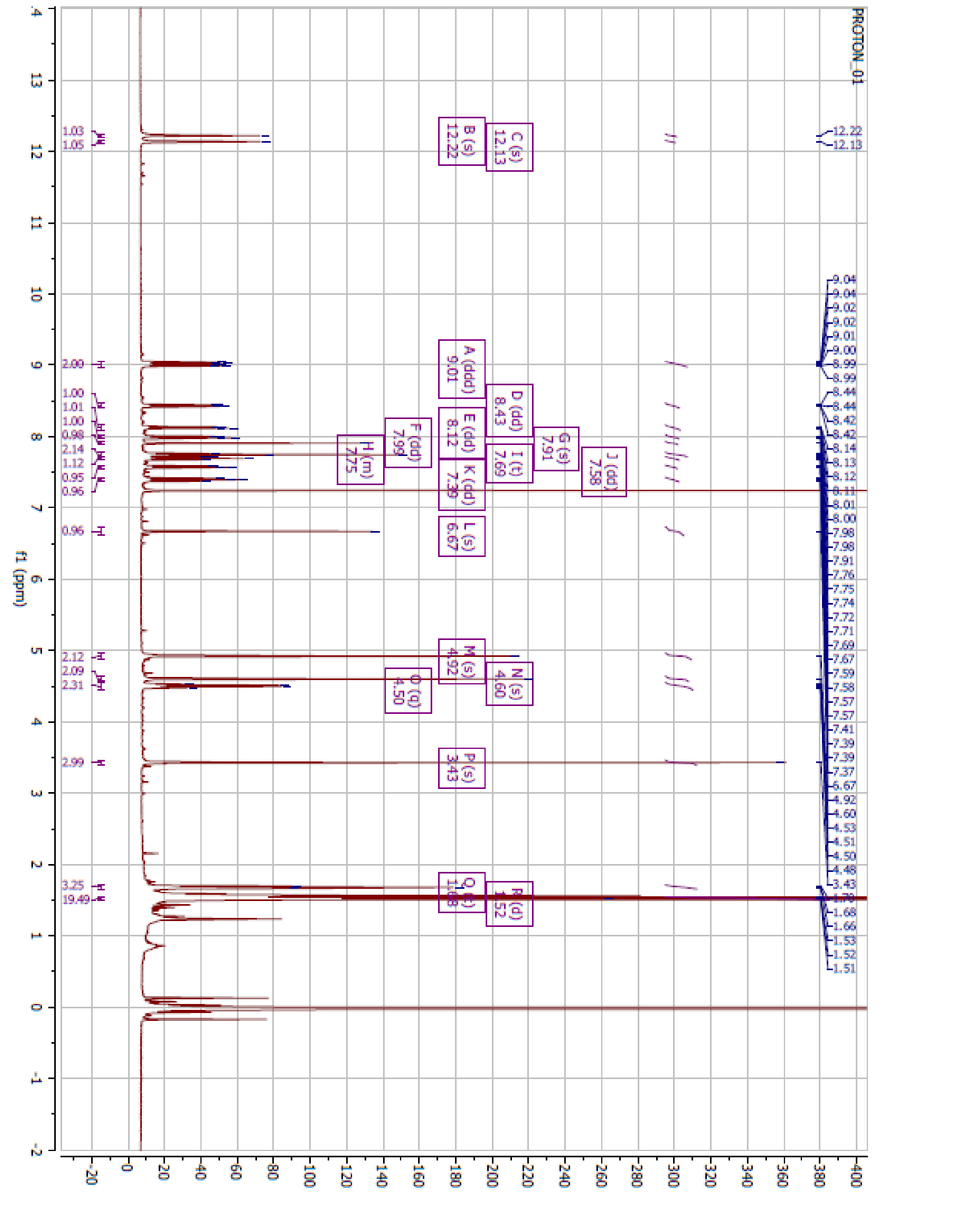

Supplementary Figure 18. ^{1}H-NMR of 9.

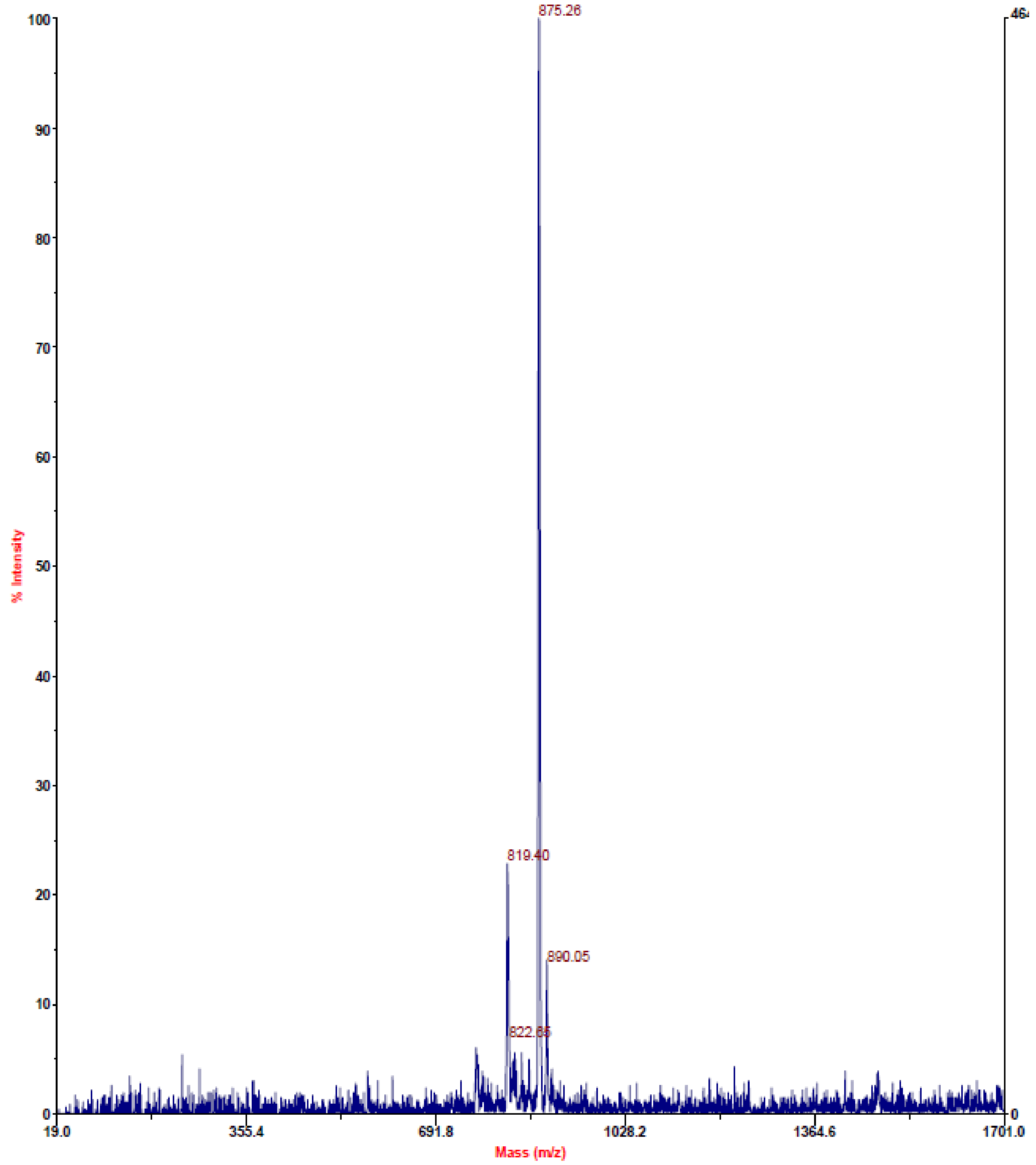

Supplementary Figure 19. MALDI-TOF of 9.

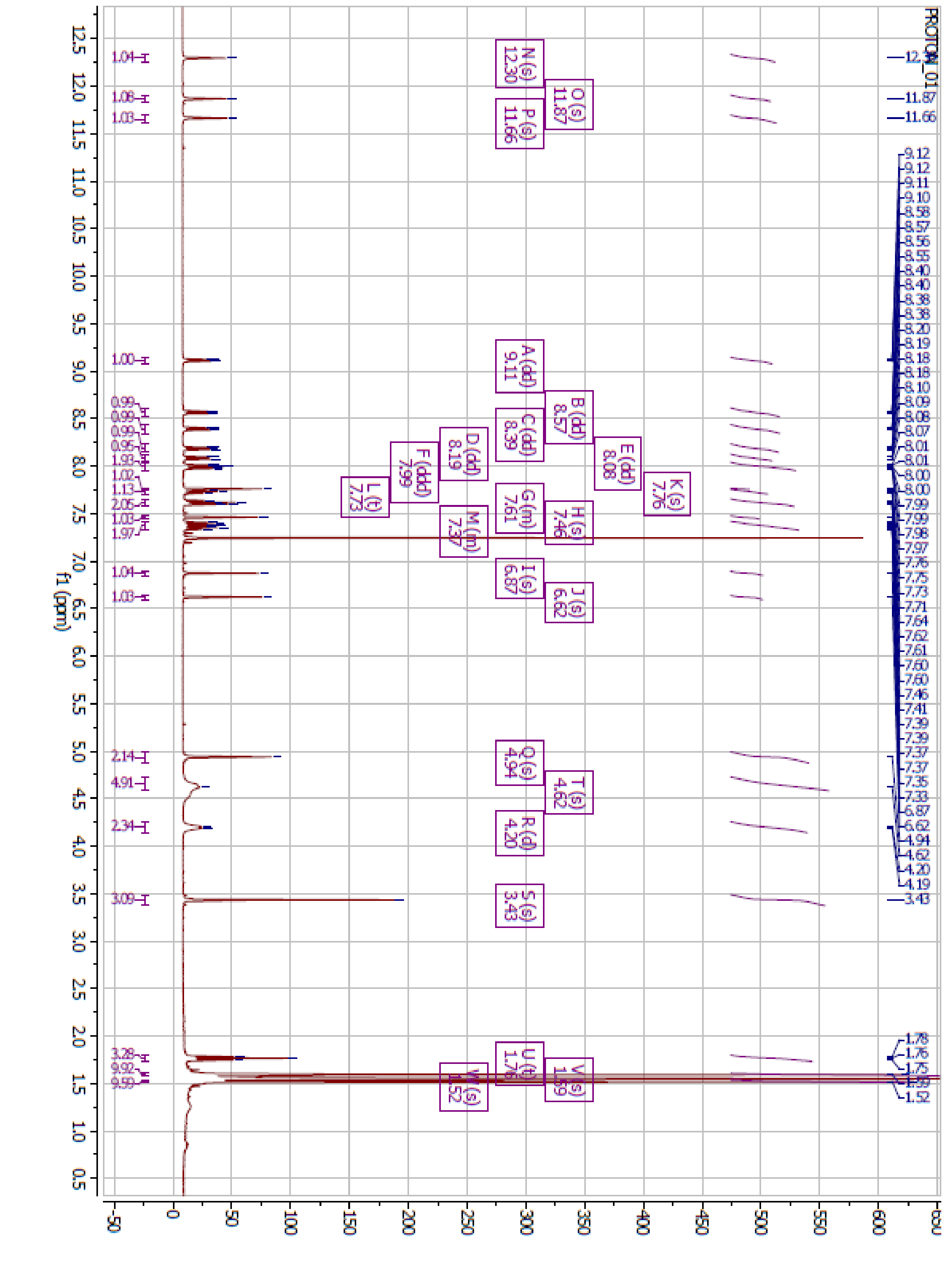

Supplementary Figure 20. ^{1}H-NMR of 10.

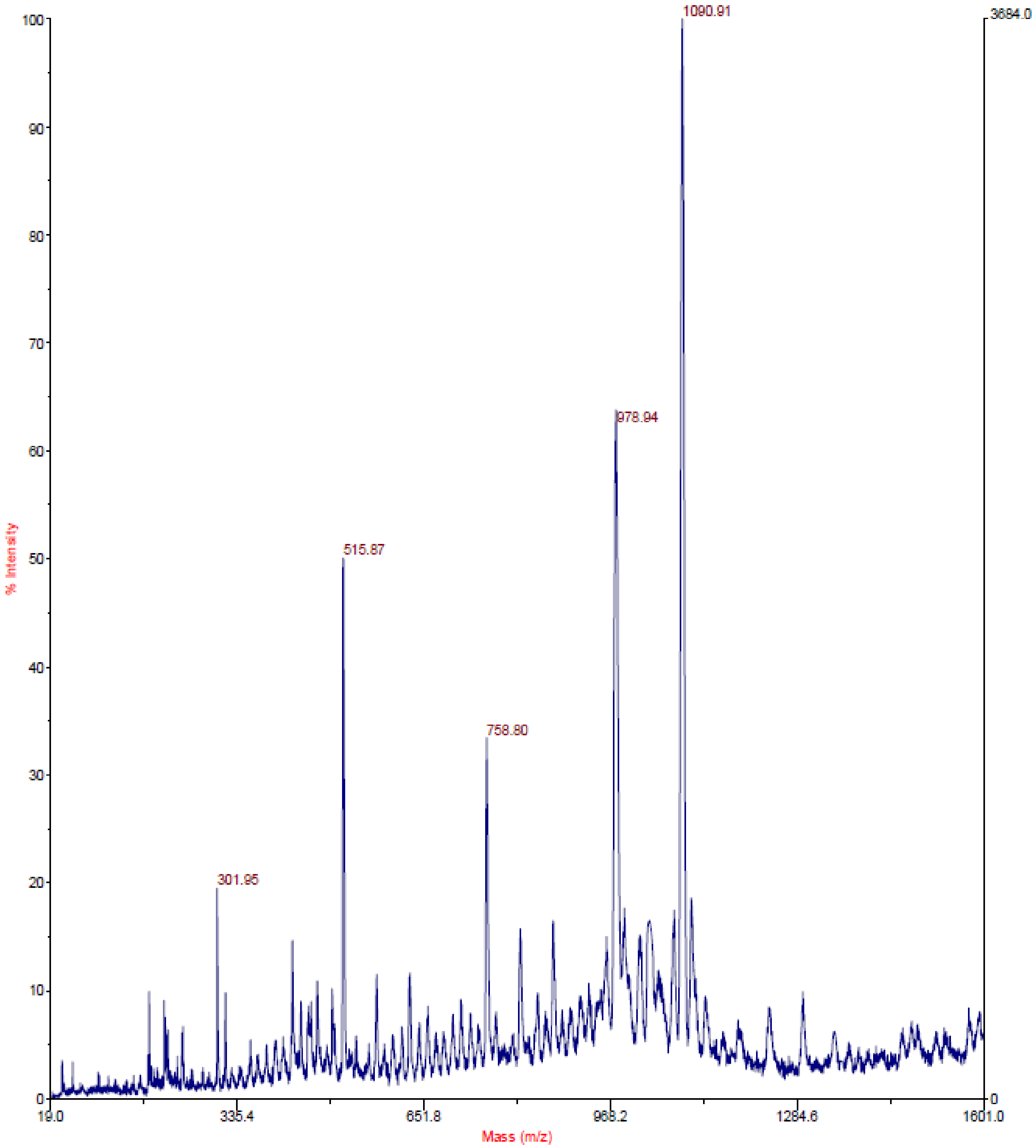

Supplementary Figure 21. MALDI-TOF of 10.

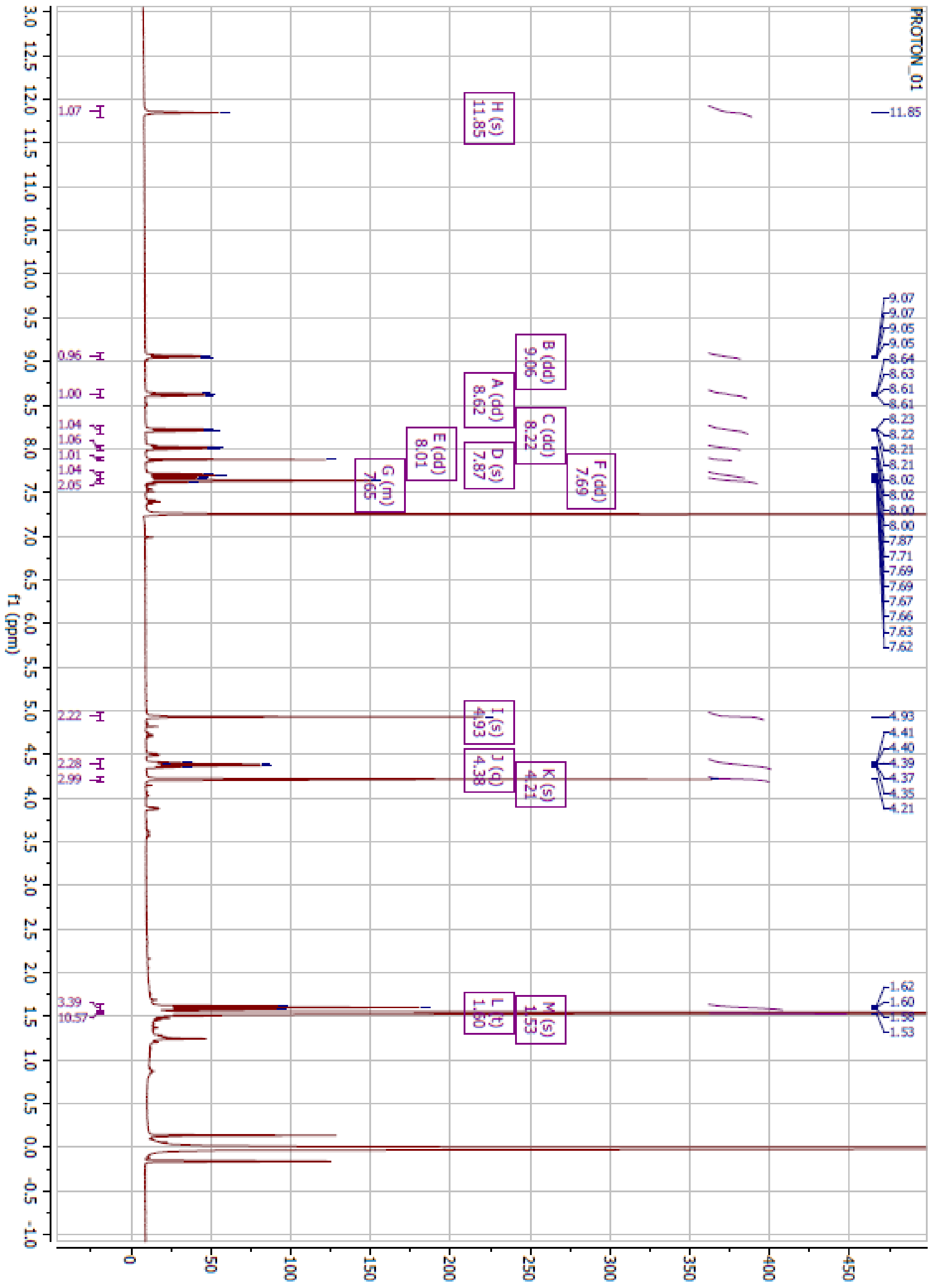

Supplementary Figure 22. ^{1}H-NMR of 11.

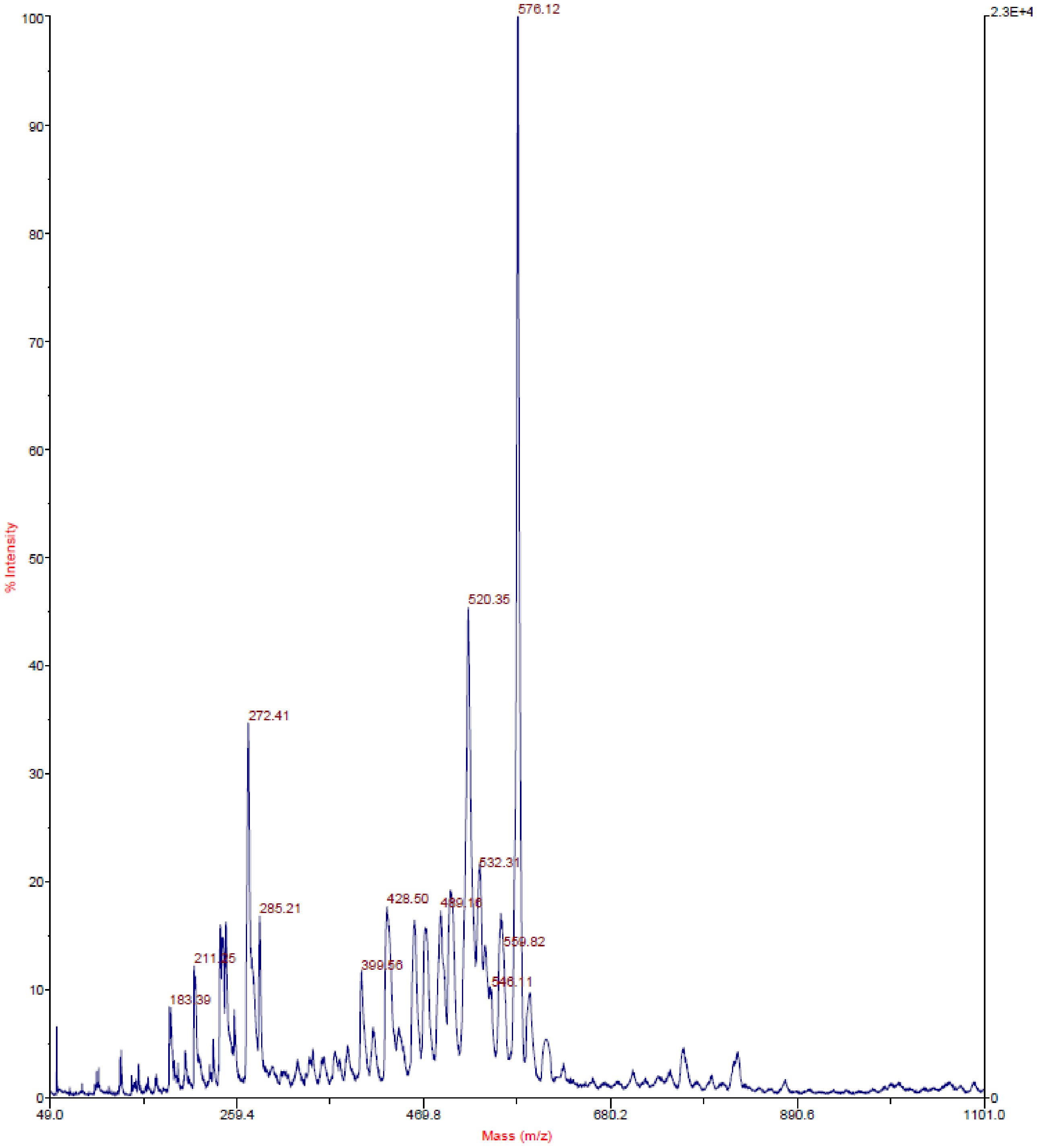

Supplementary Figure 23. MALDI-TOF of 11.

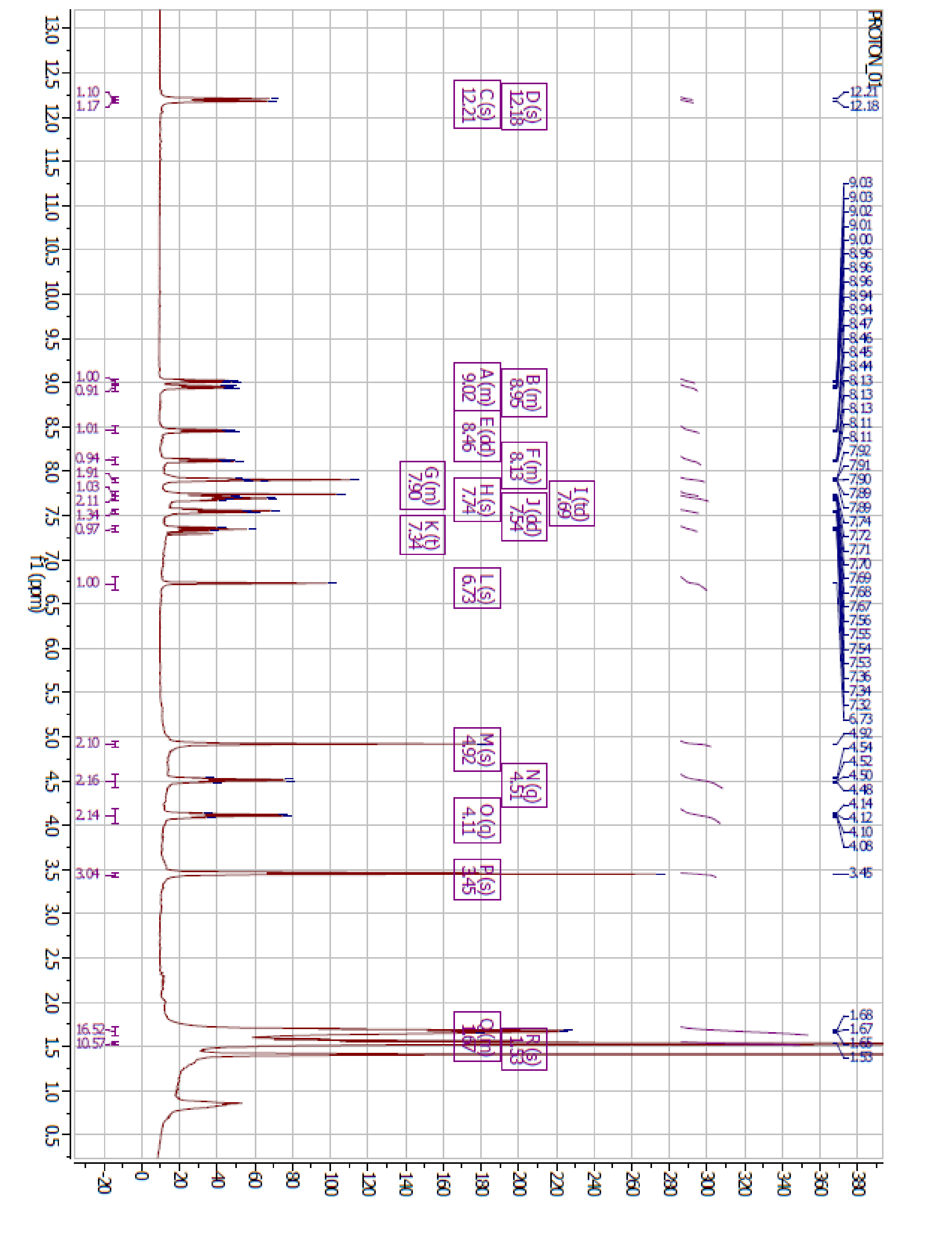

Supplementary Figure 24. ^{1}H-NMR of 12.

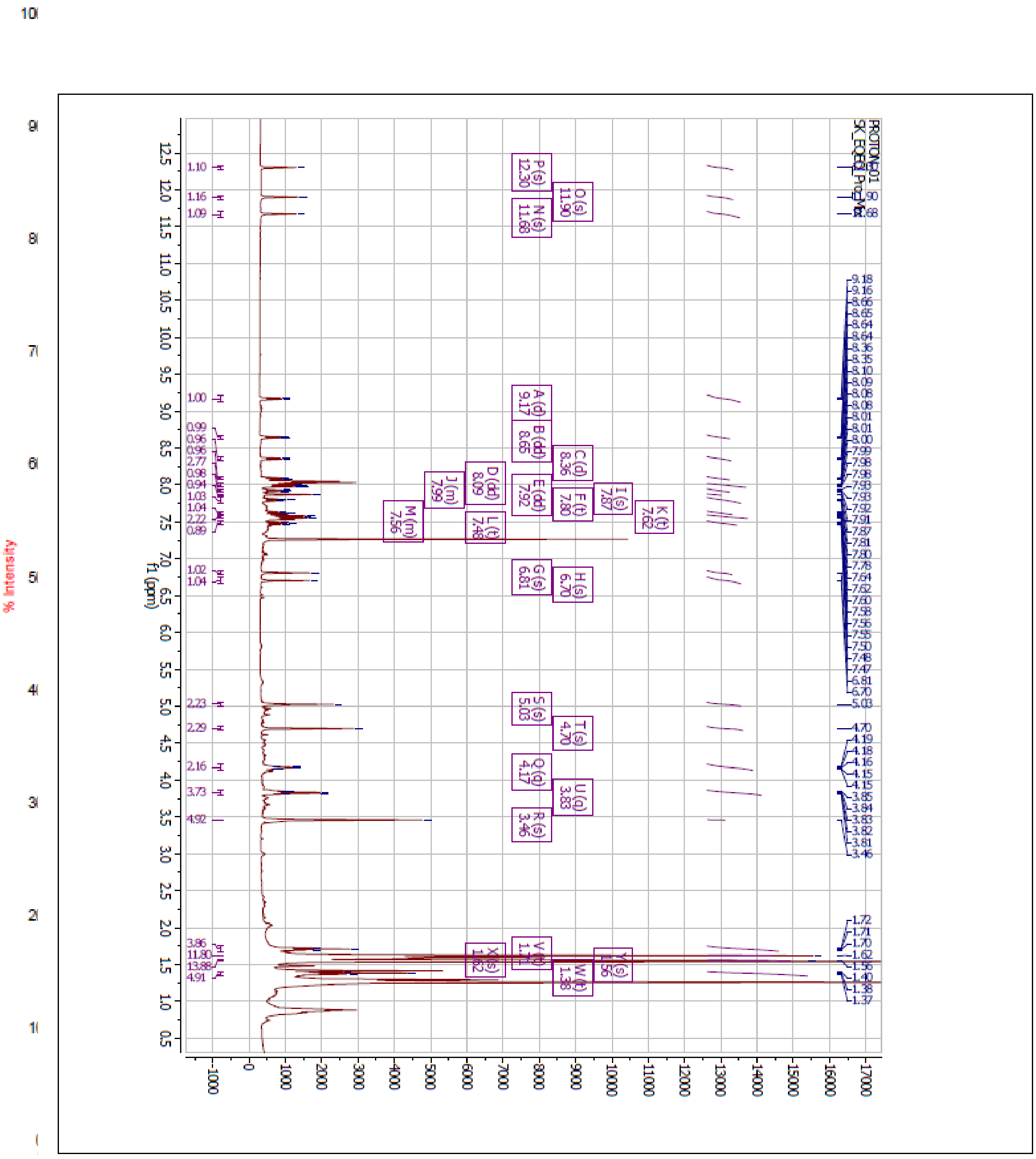

Supplementary Figure 25. MALDI-TOF of 12.

Supplementary Figure 26. ^{1}H-NMR of 13.

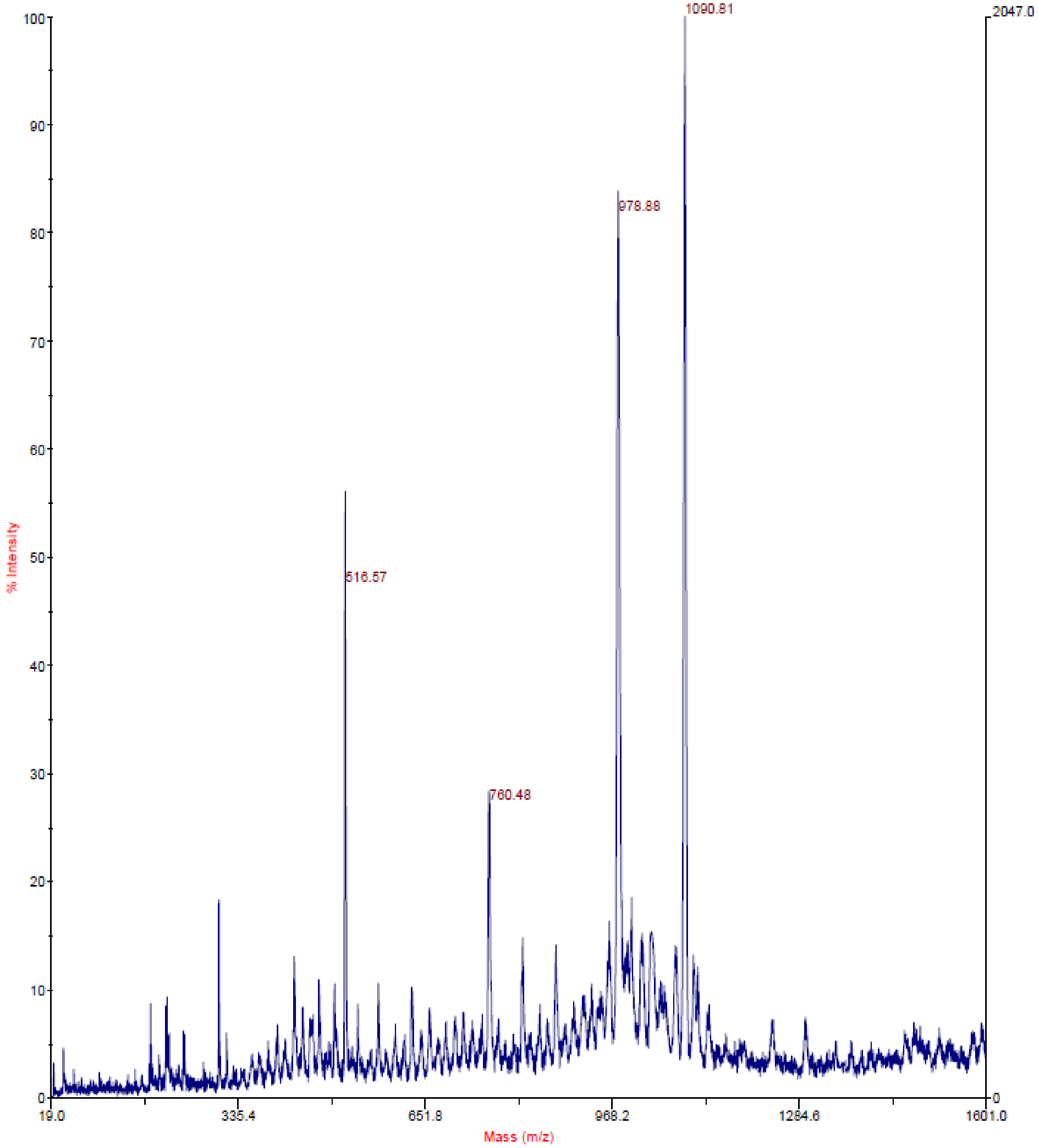

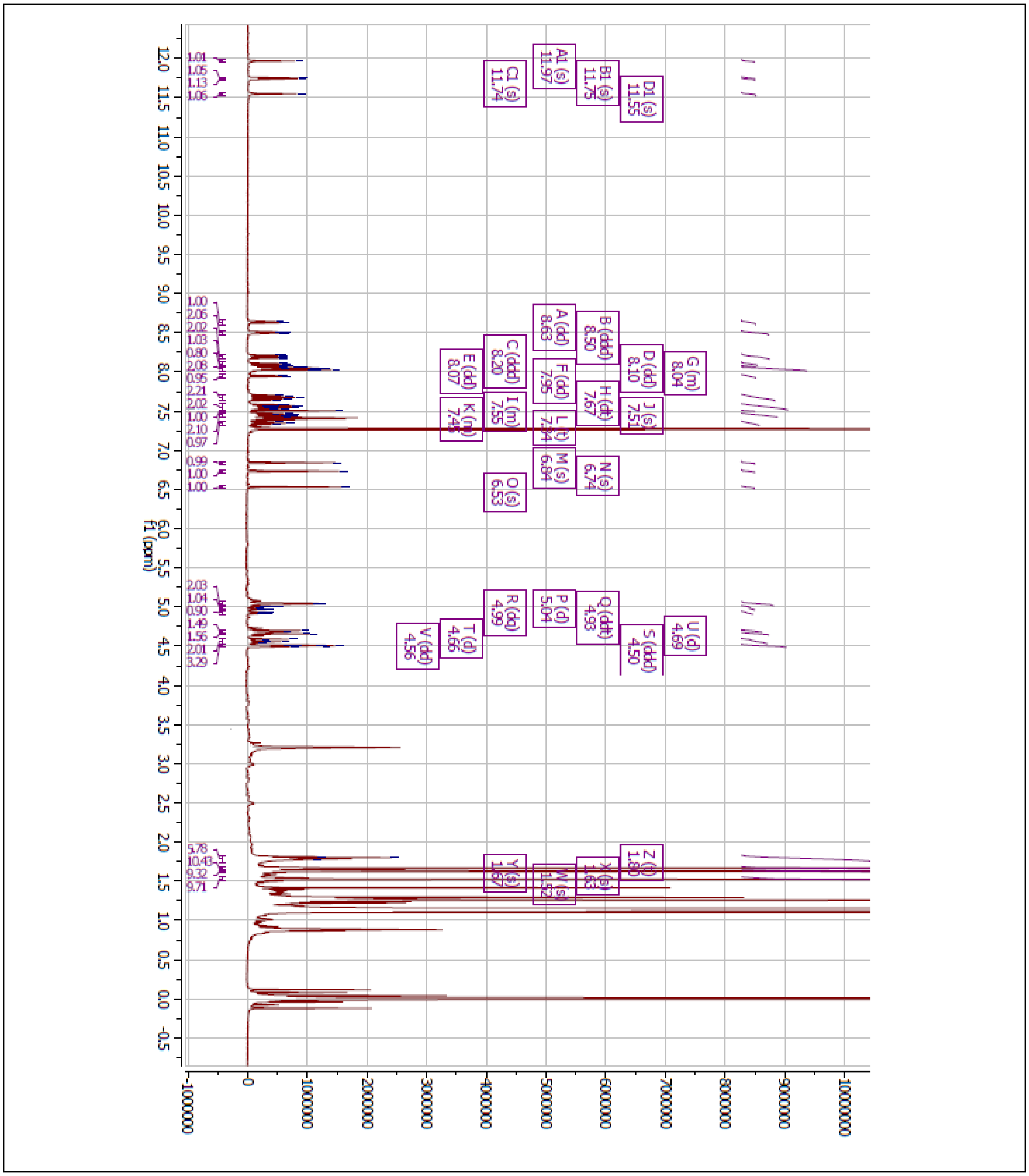

Supplementary Figure 27. MALDI-TOF of 13.

Supplementary Figure 28. ^{1}H-NMR of tert-butyl of ADM-116l.

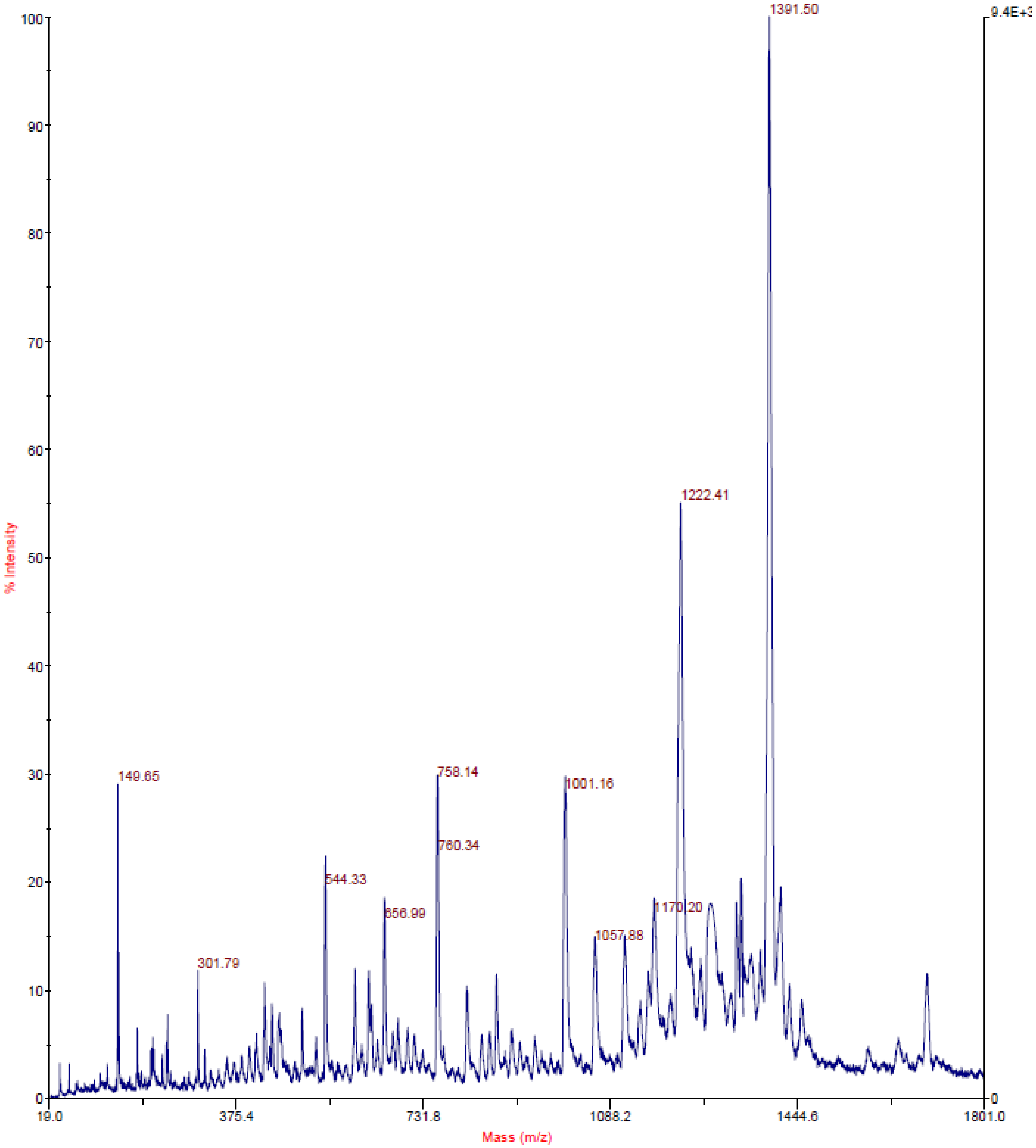

Supplementary Figure 29. MALDI-TOF of Tert-butyl of ADM-116l.

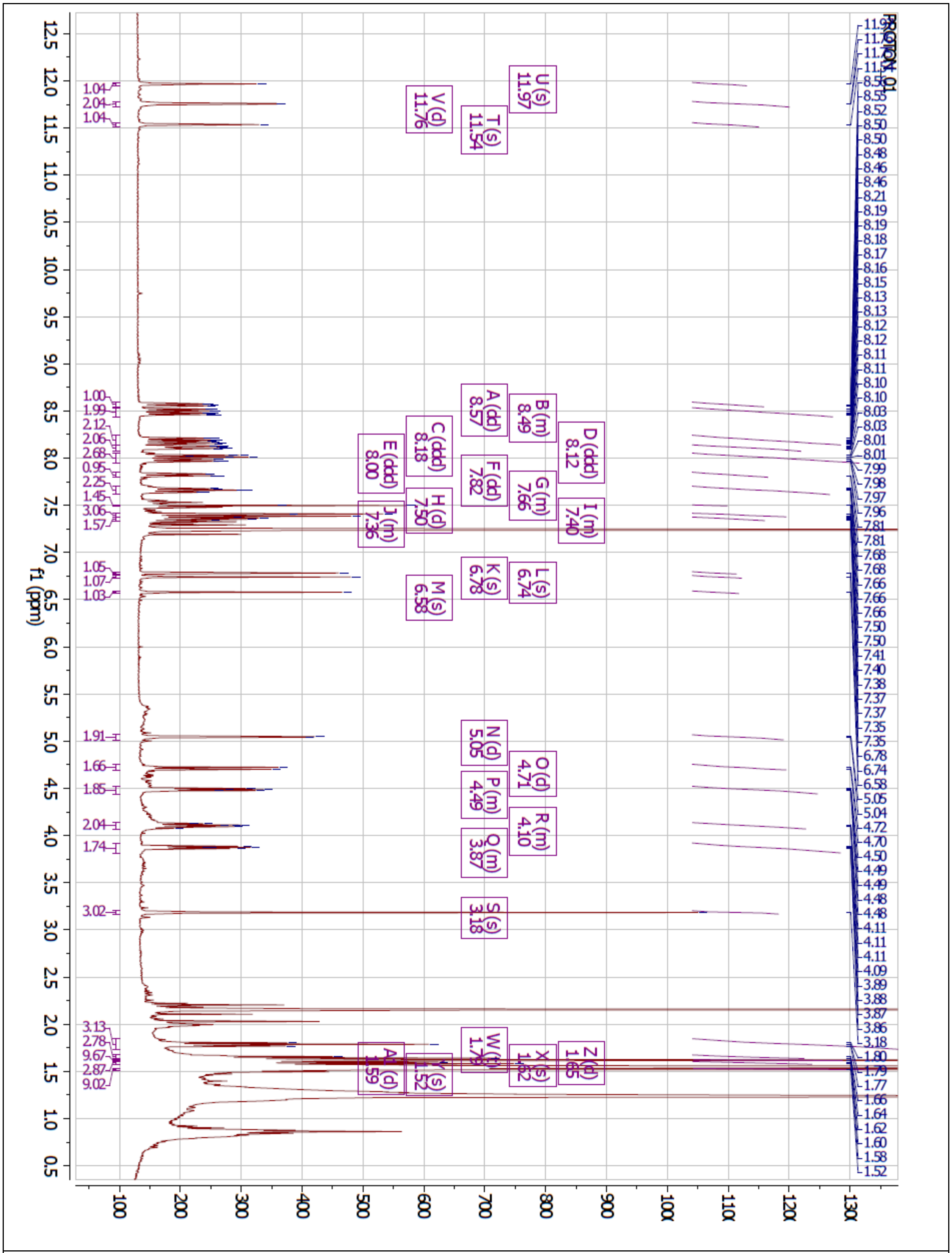

Supplementary Figure 30. ^{1}H-NMR of *tert*-butyl of ADM-|116.

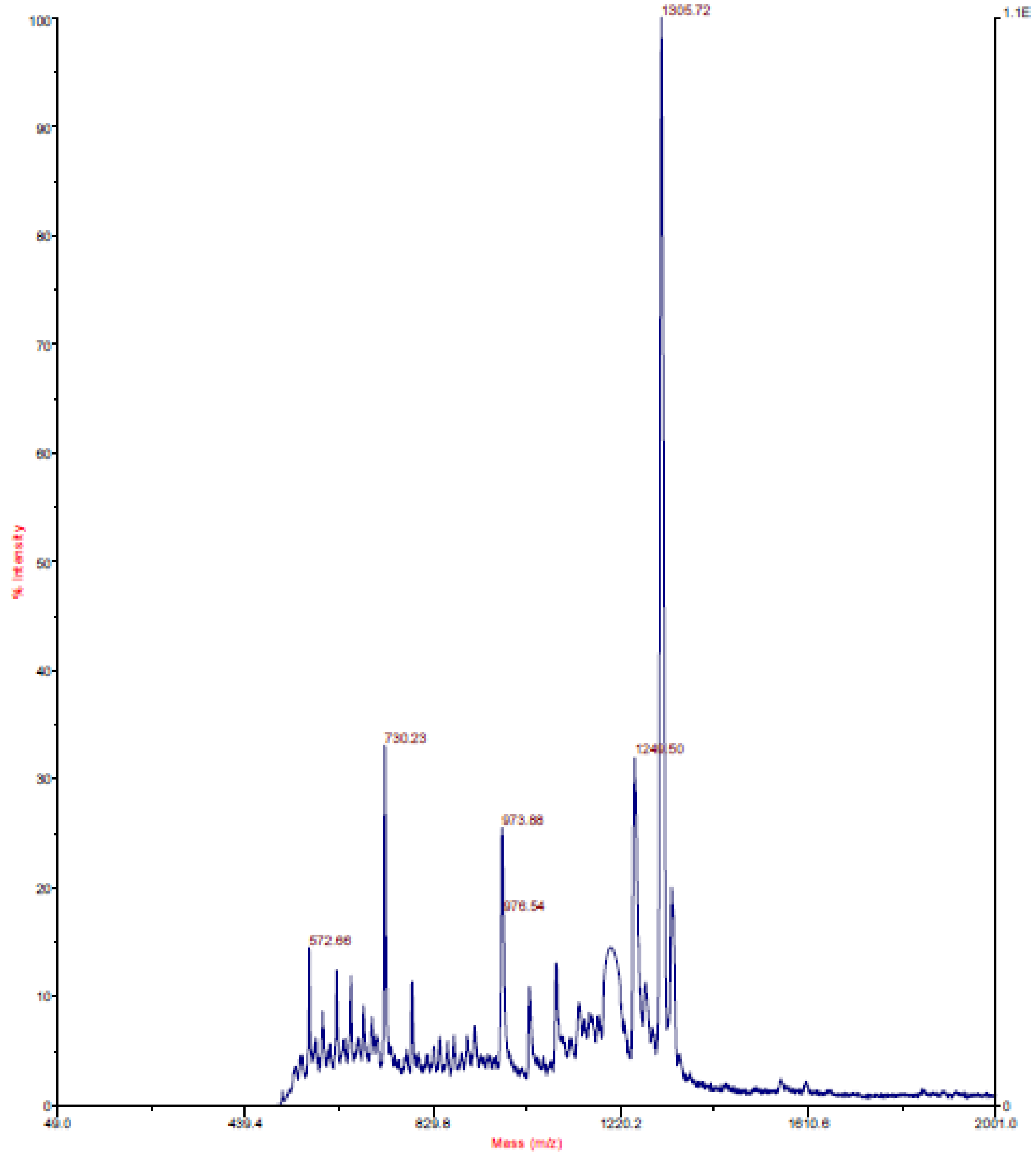

Supplementary Figure 31. MALDI-TOF of *tert*-butyl of ADM-Ⅰ116.

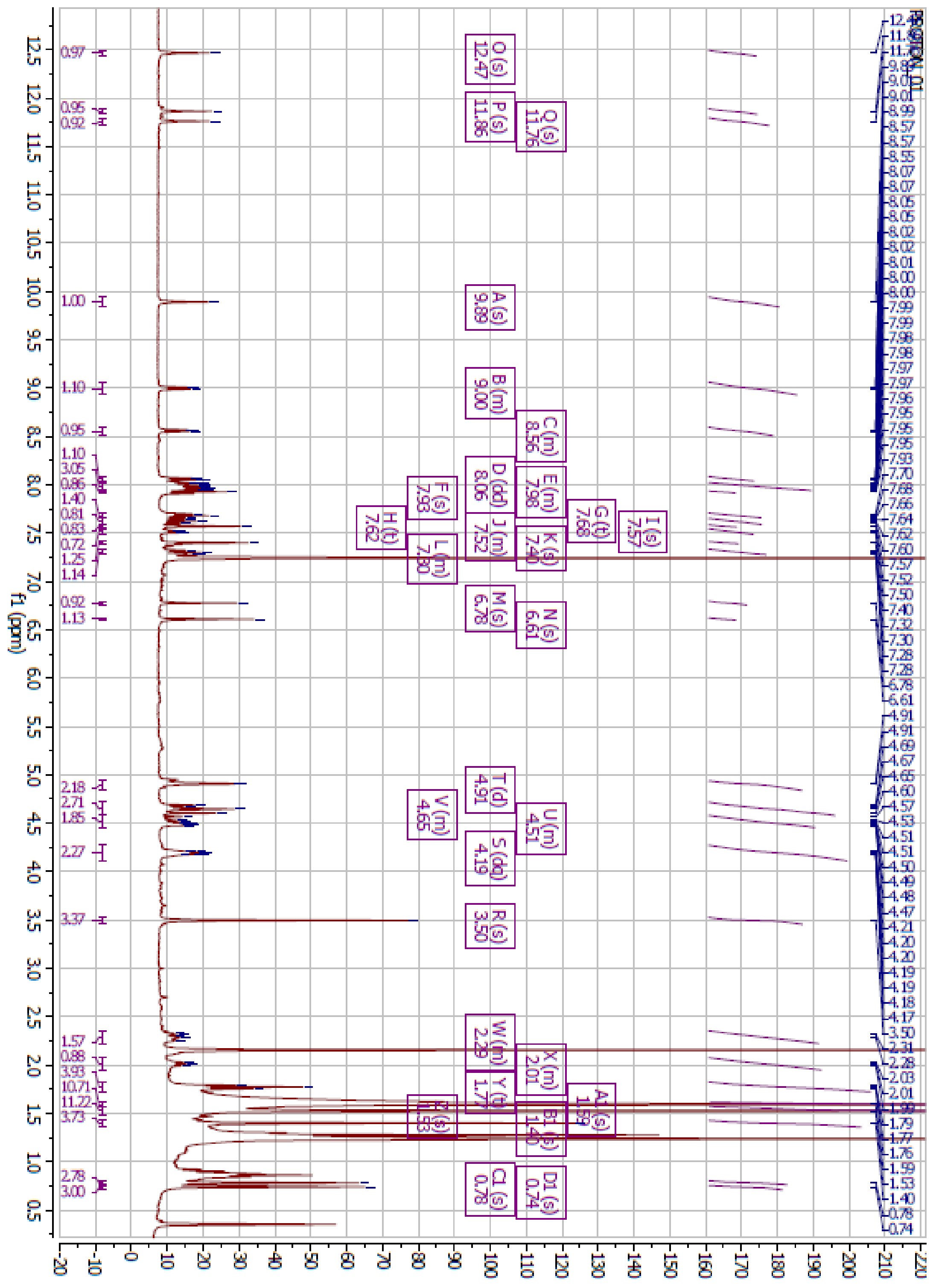

Supplementary Figure 32. ^{1}H-NMR of *tert*-butyl of ADM-116$_{P}$.

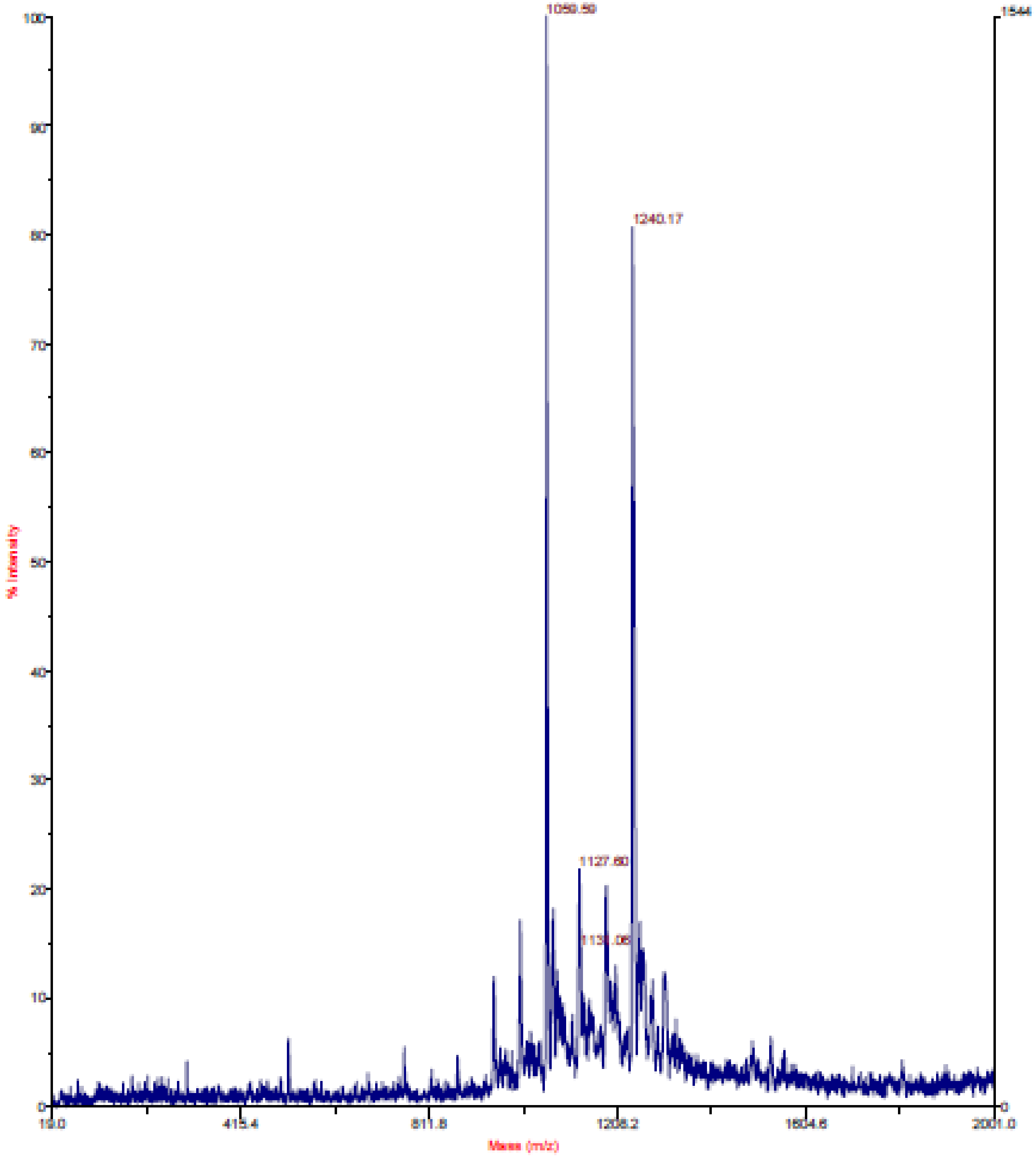

Supplementary Figure 33. MALDI-TOF of *tert*-butyl of ADM-116$_{P}$.

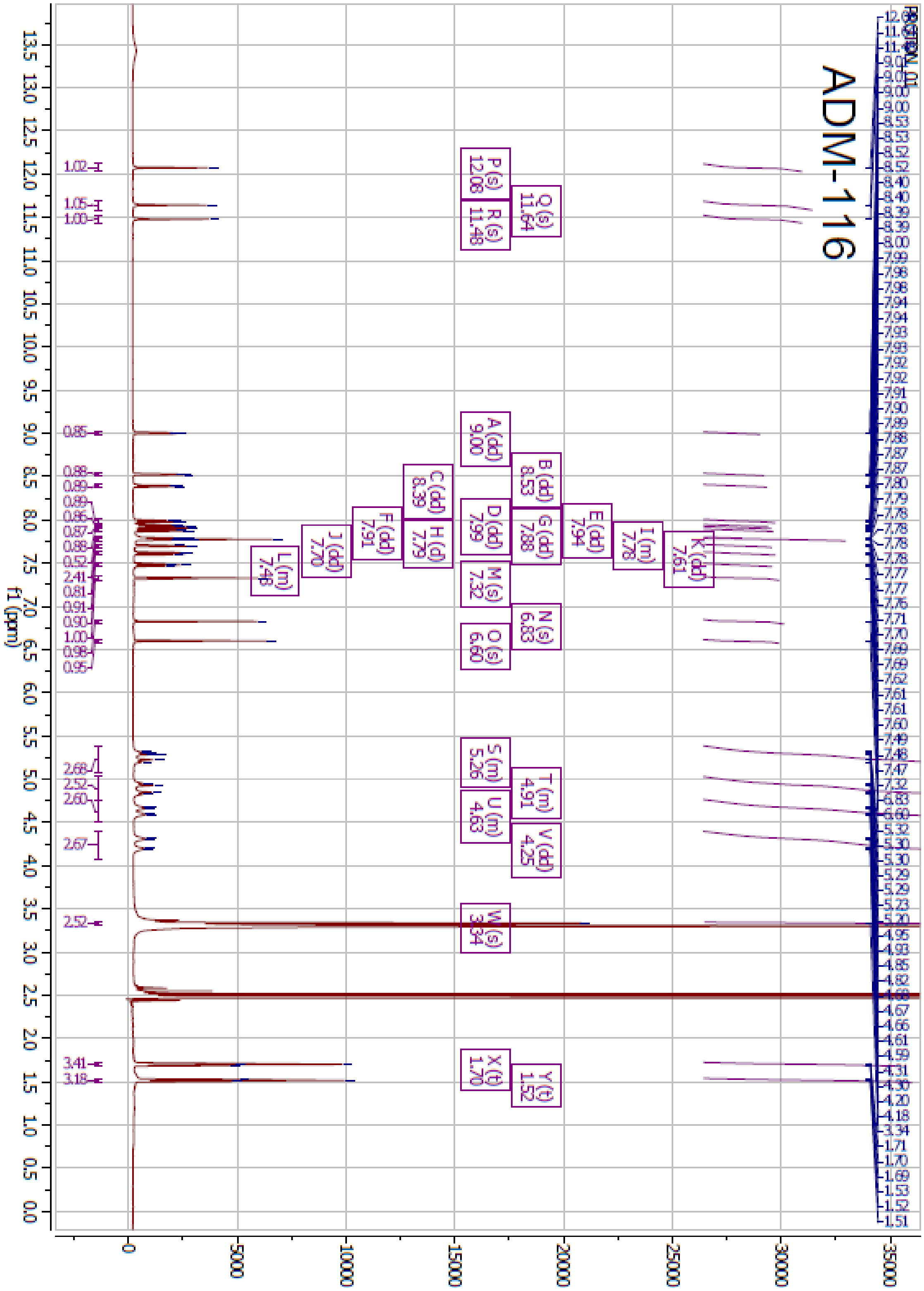

Supplementary Figure 34. ^{1}H-NMR of ADM-116.

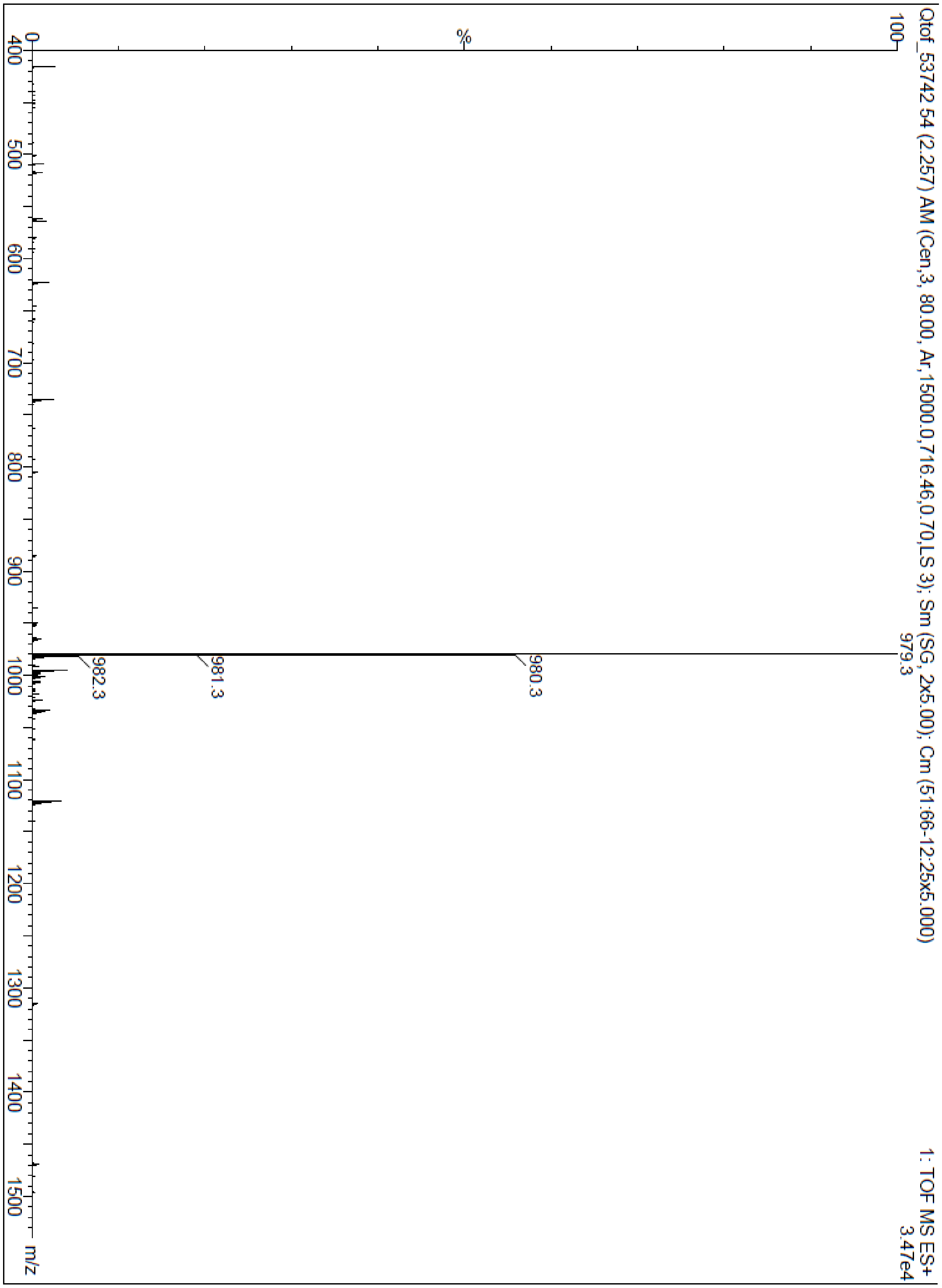

Supplementary Figure 35. ESI-MS of ADM-116.

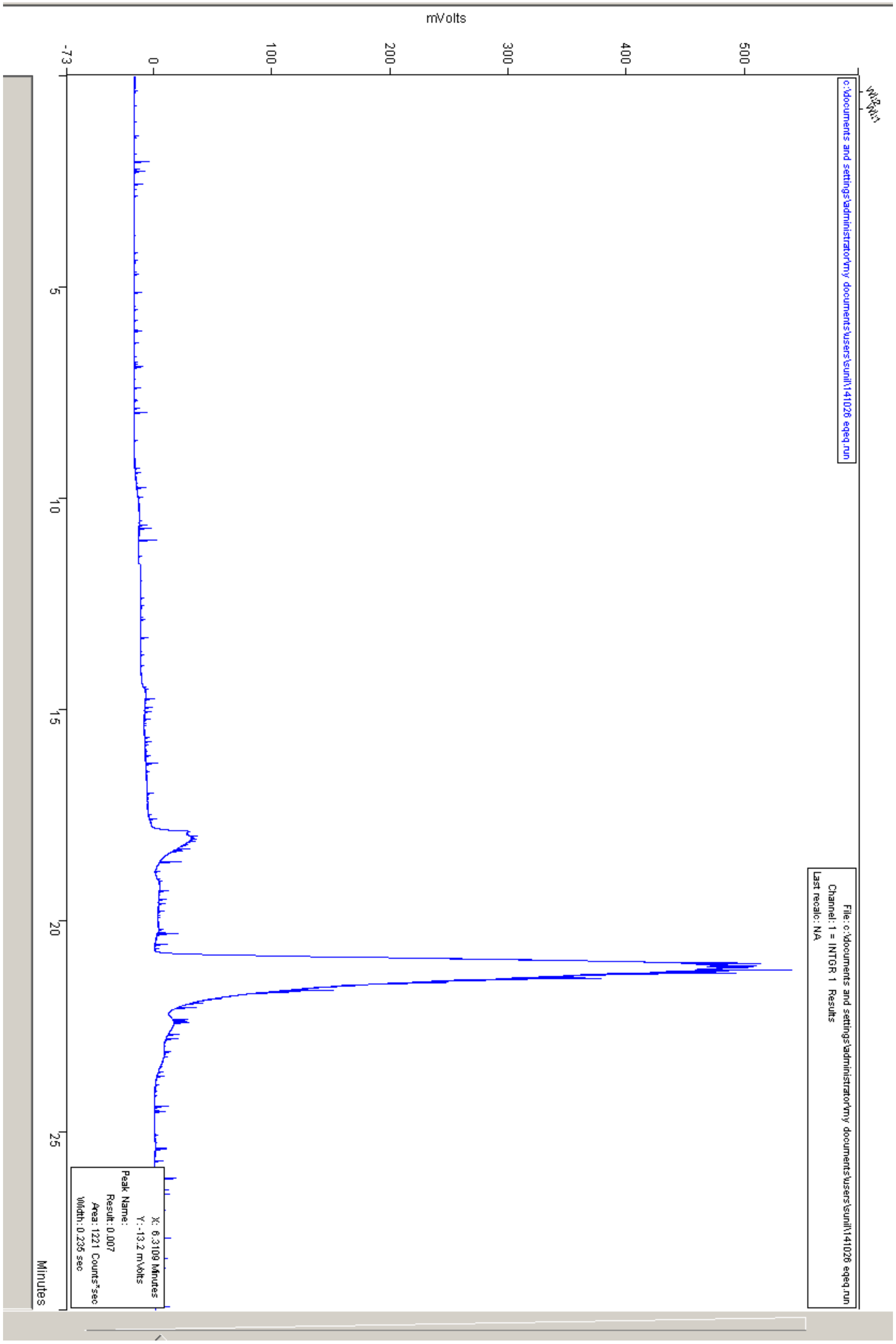

Supplementary Figure 36. RP-HPLC of ADM-116.

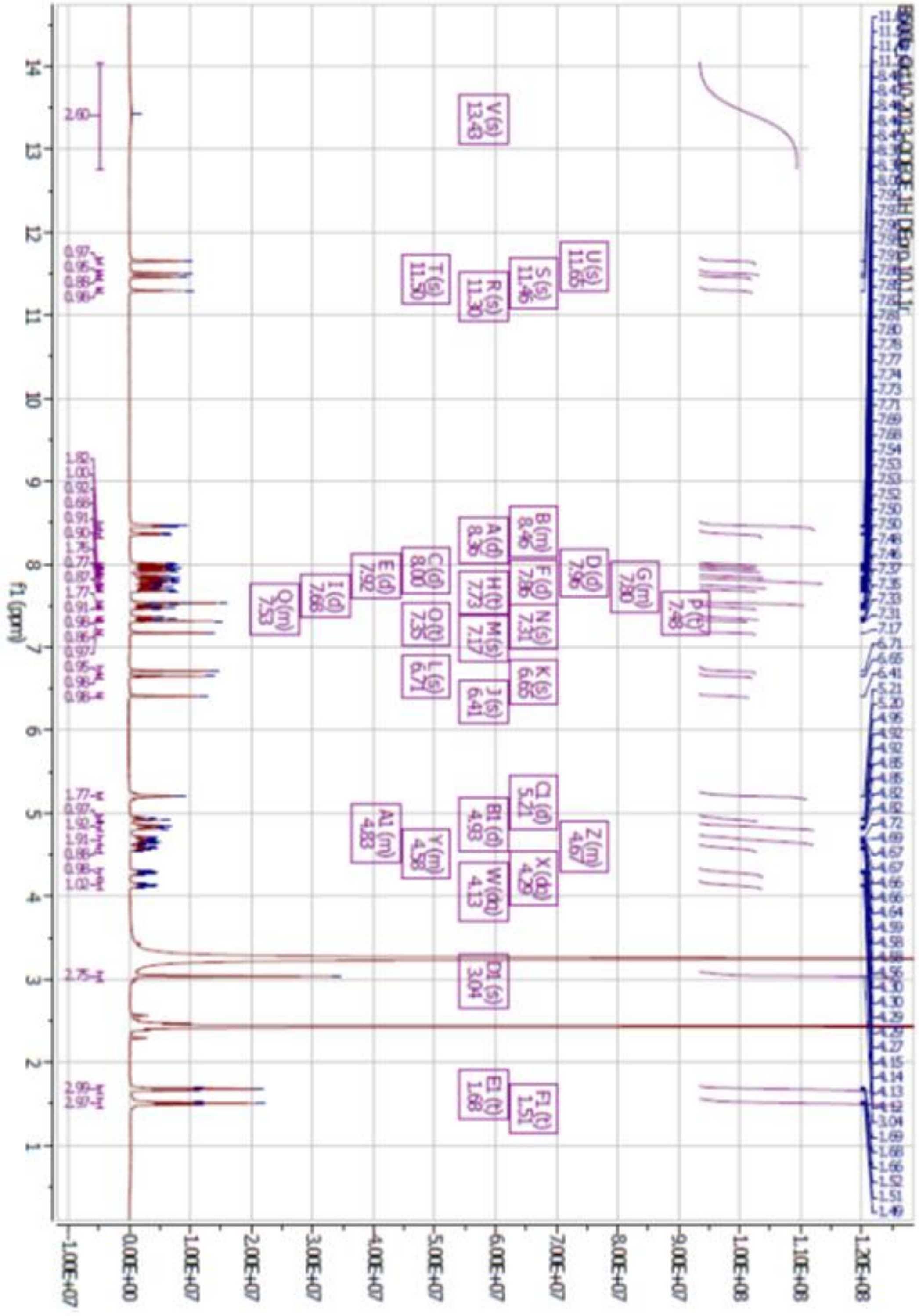

Supplementary Figure 37. ^{1}H-NMR of ADM-|116.

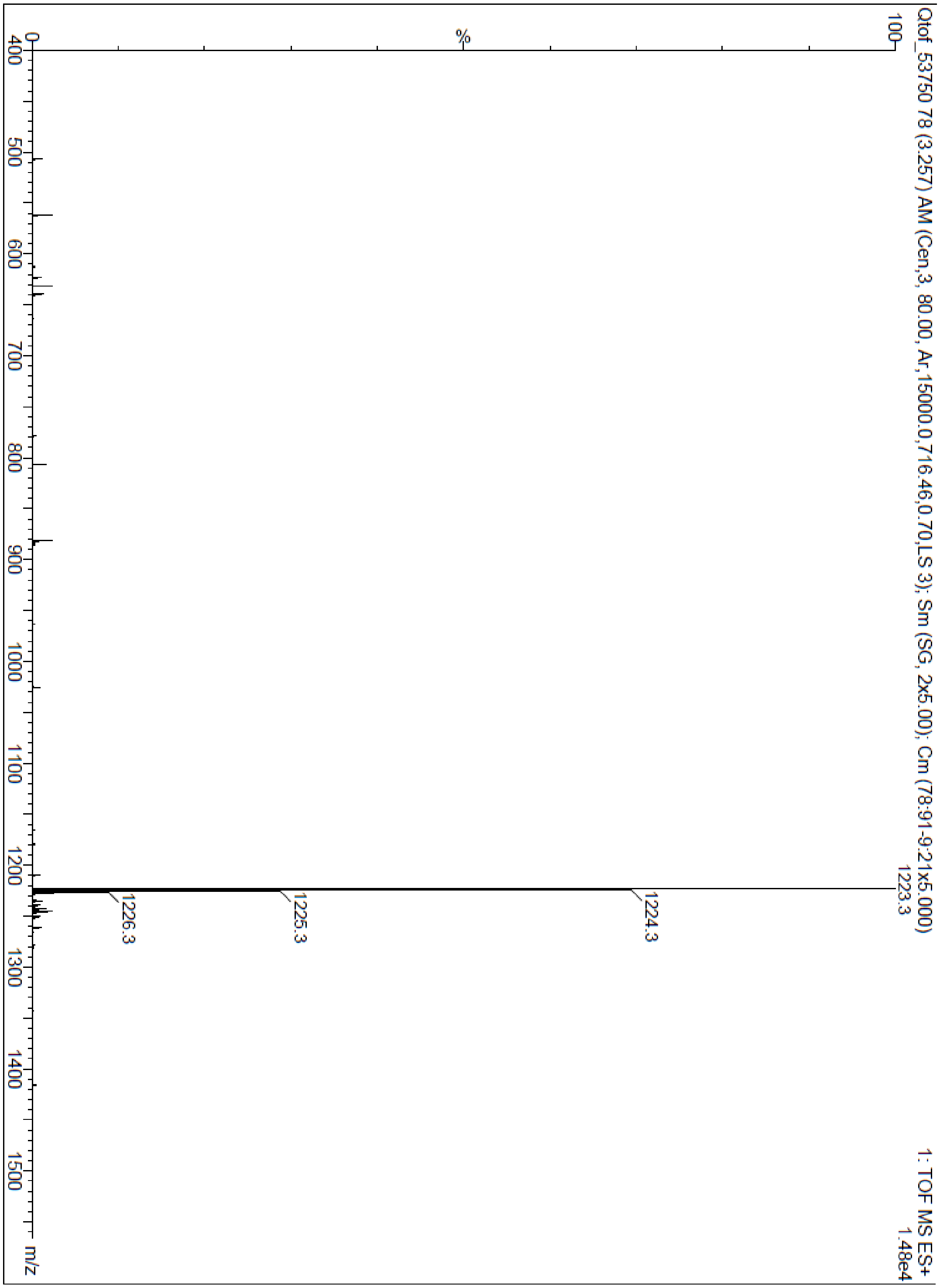

Supplementary Figure 38. ESI-MS of ADM-ǀ116.

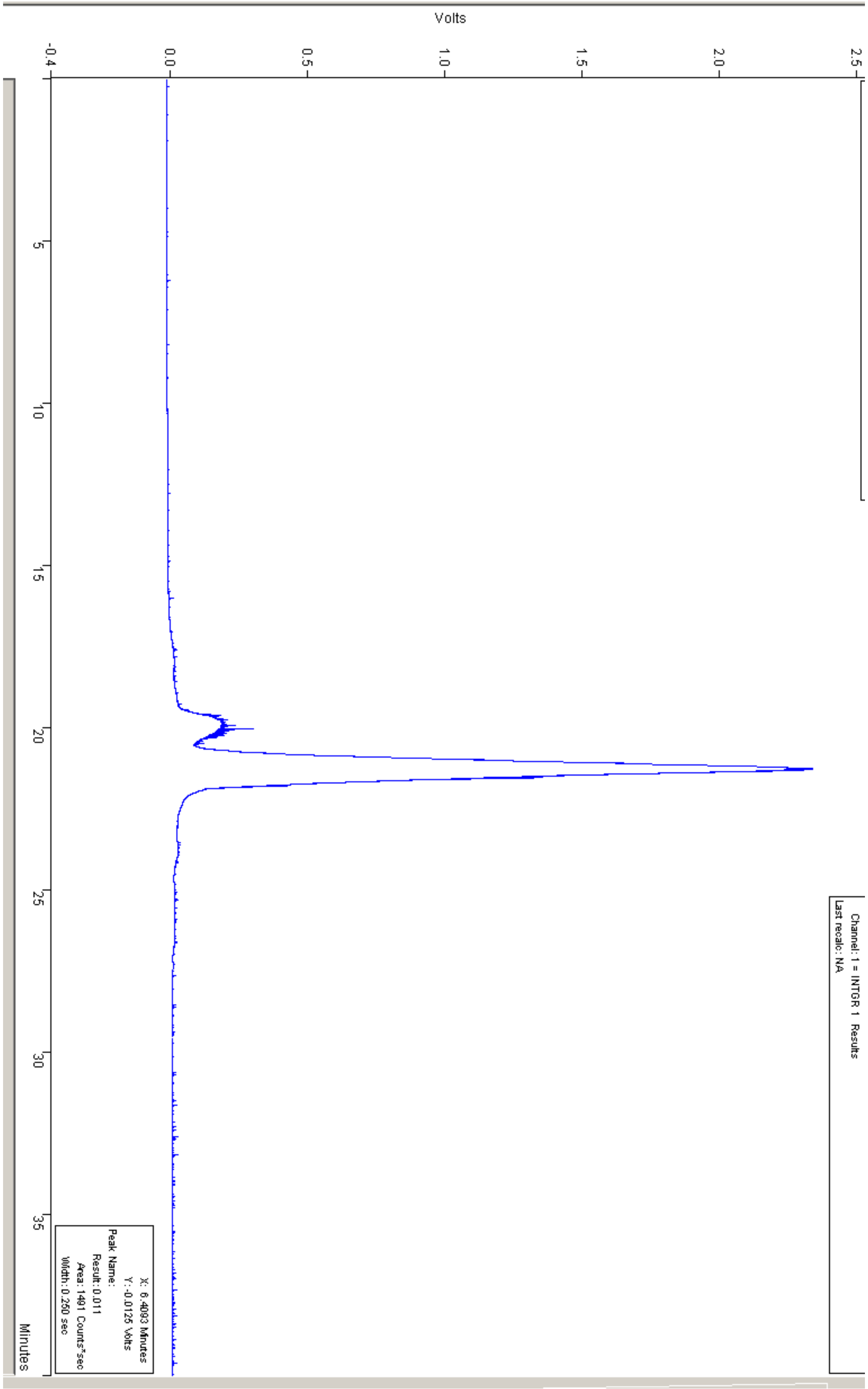

Supplementary Figure 39. RP-HPLC of ADM-I116.

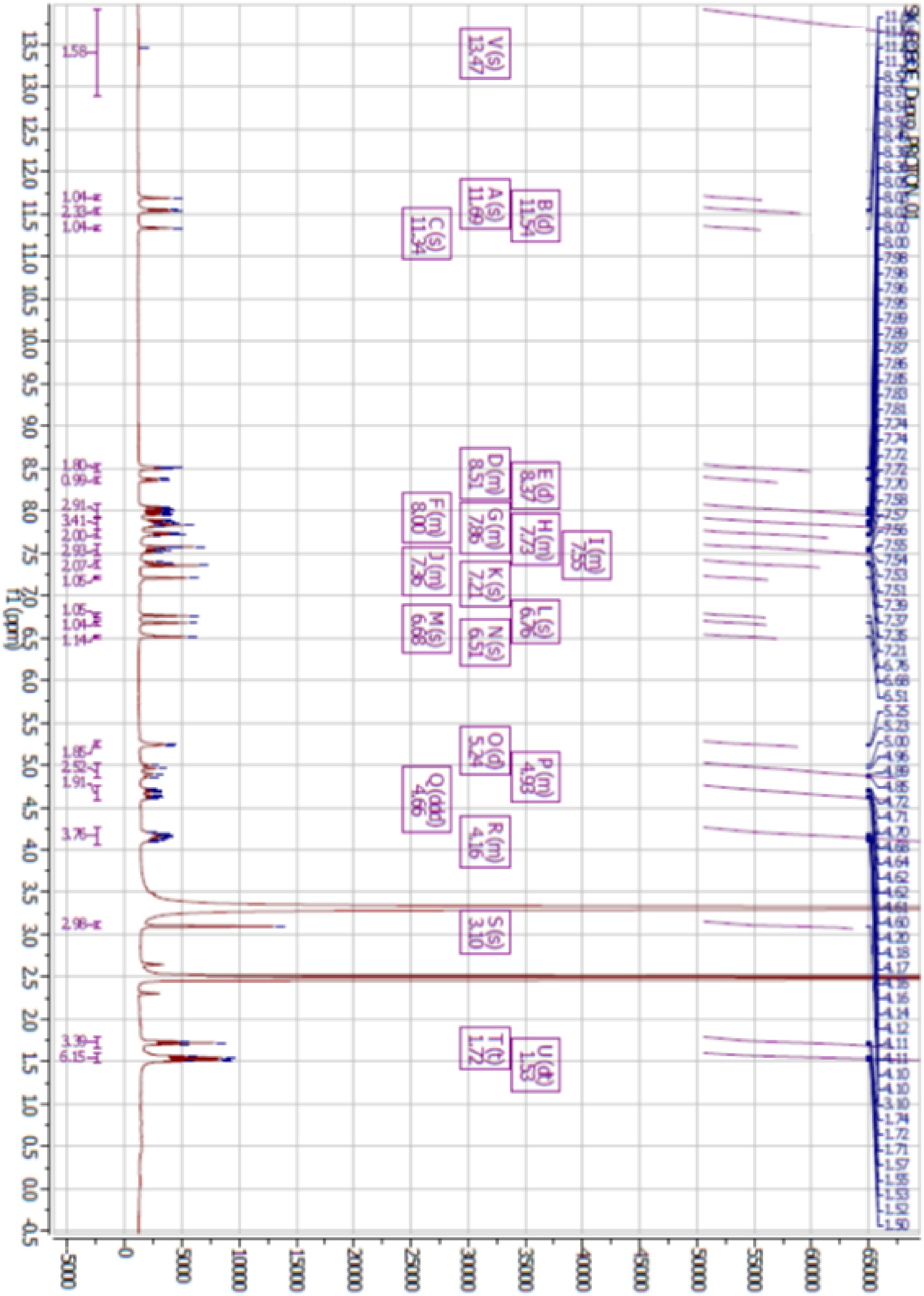

Supplementary Figure 40. ^{1}H-NMR of ADM-116l.

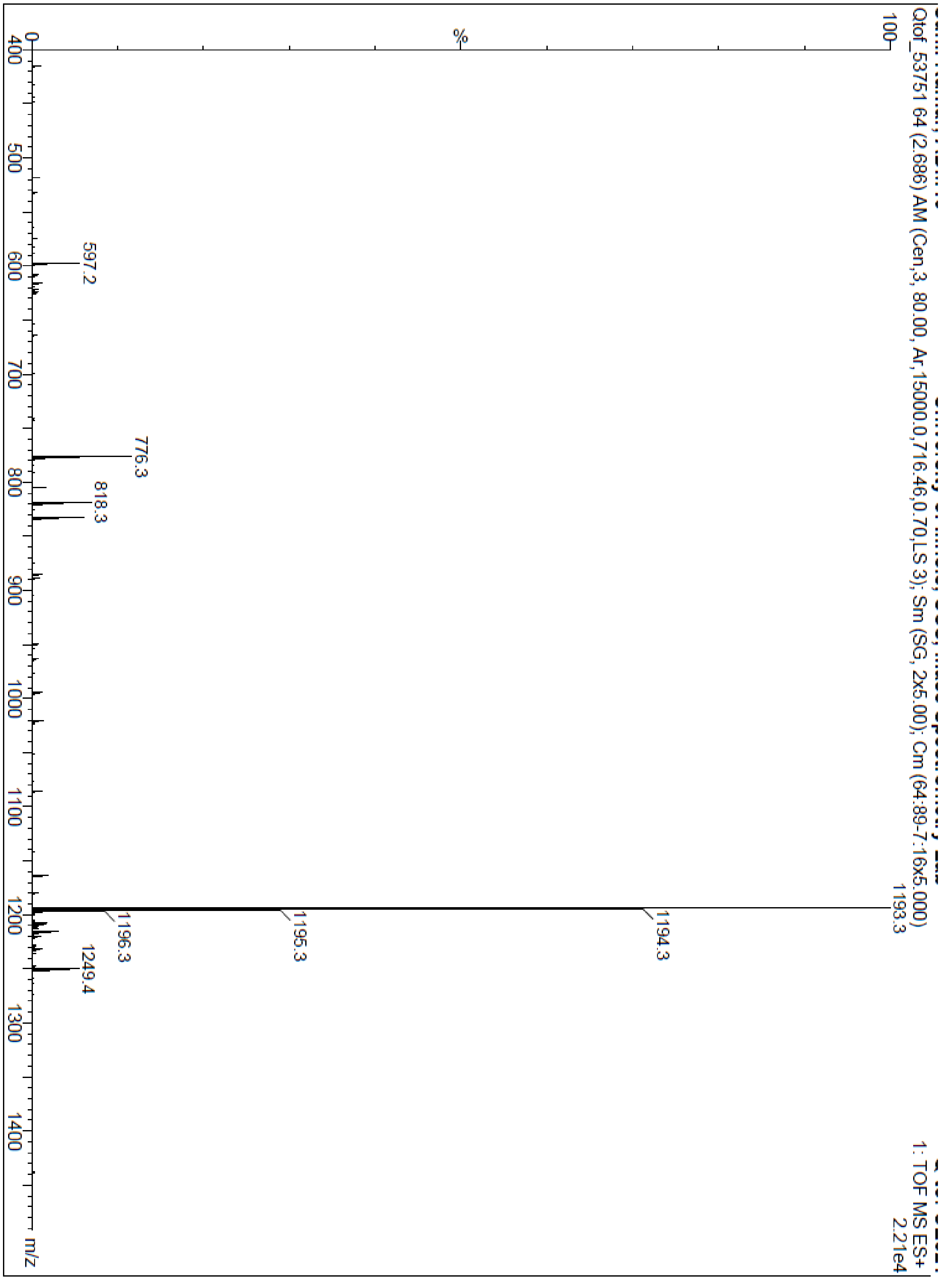

Supplementary Figure 41. ESI-MS of ADM-116l.

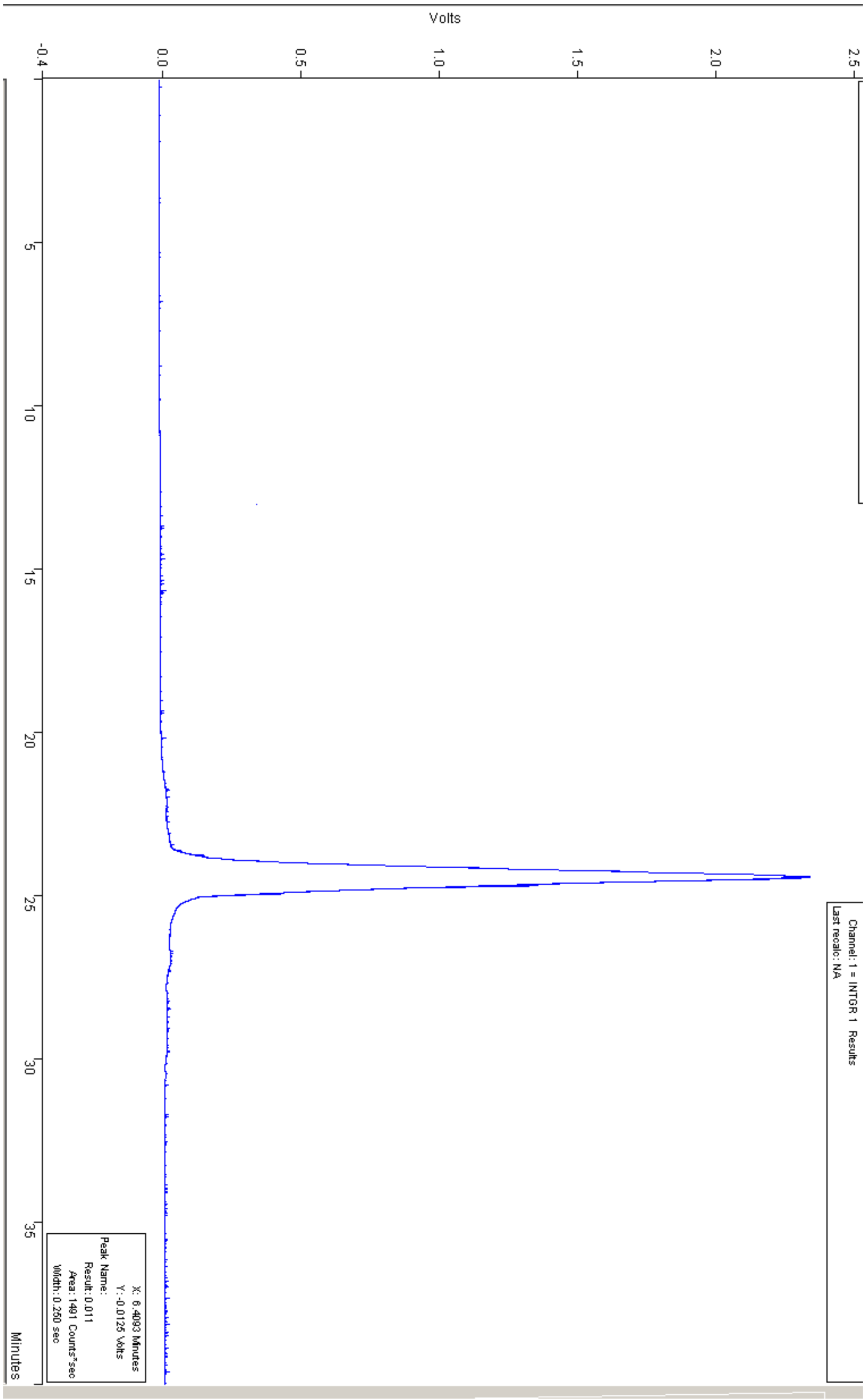

Supplementary Figure 42. RP-HPLC of ADM-116l.

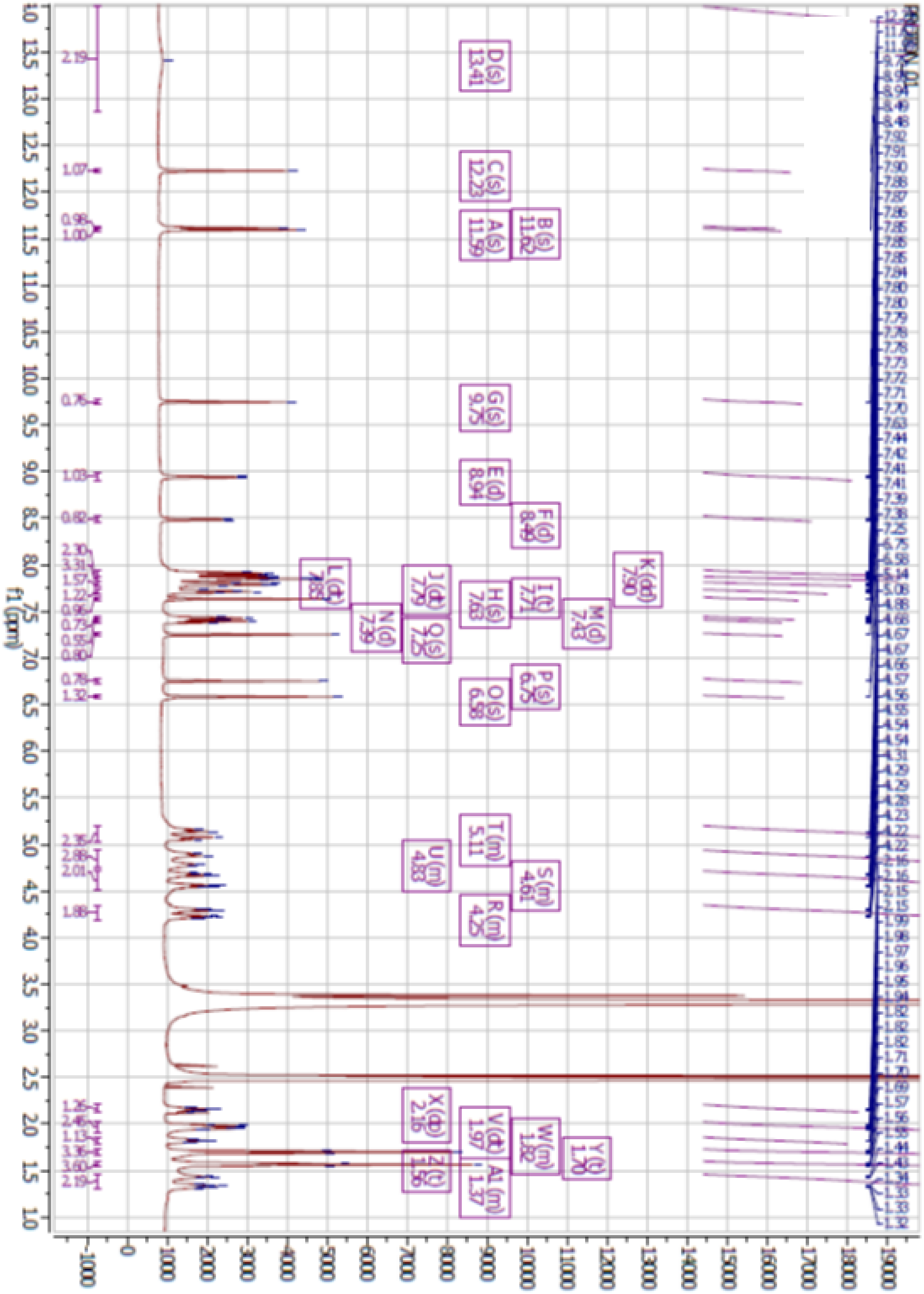

Supplementary Figure 43. ^{1}H-NMR of ADM-116$_{P}$.

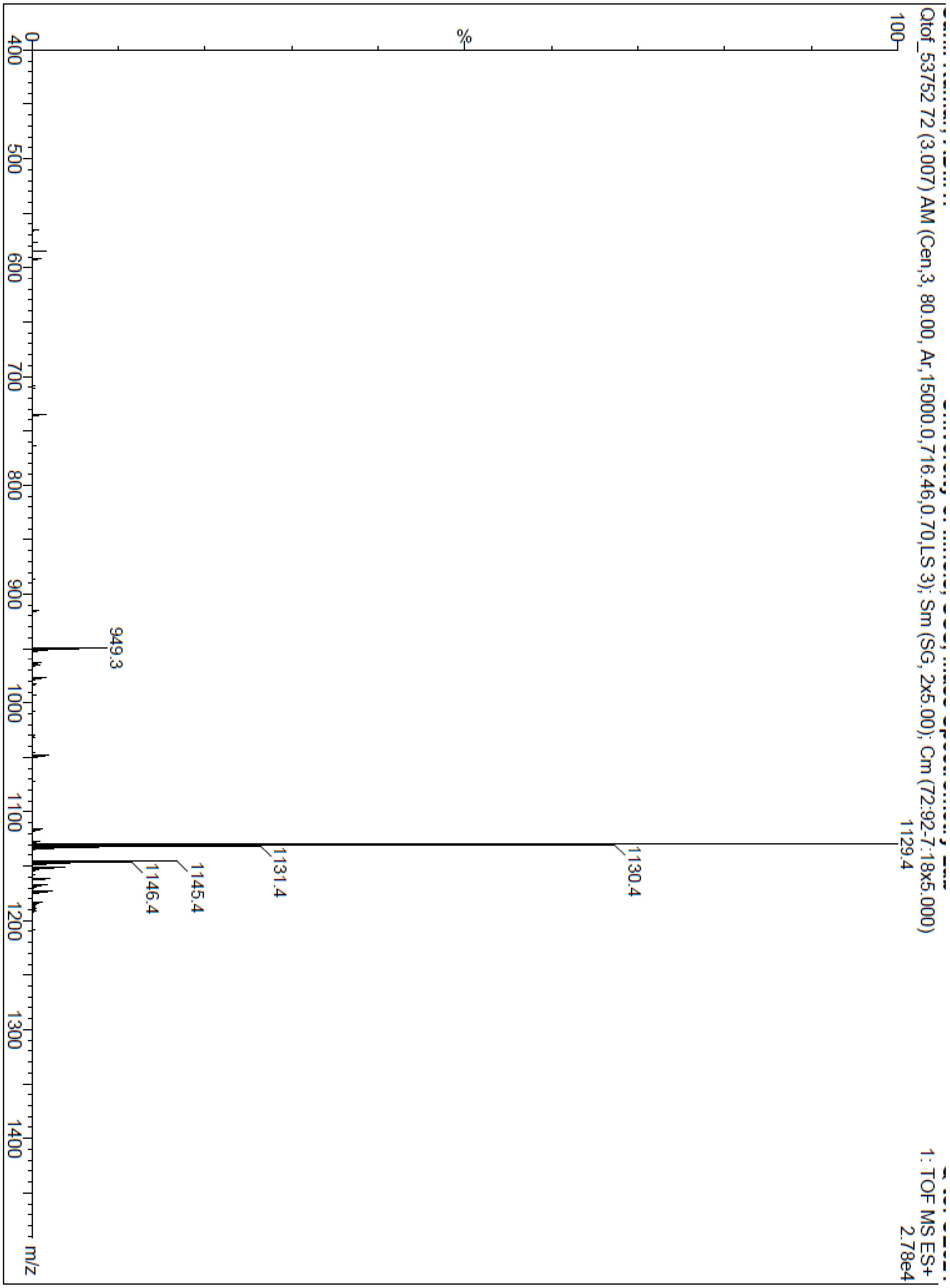

Supplementary Figure 44. ESI-MS of ADM-116$_P$.

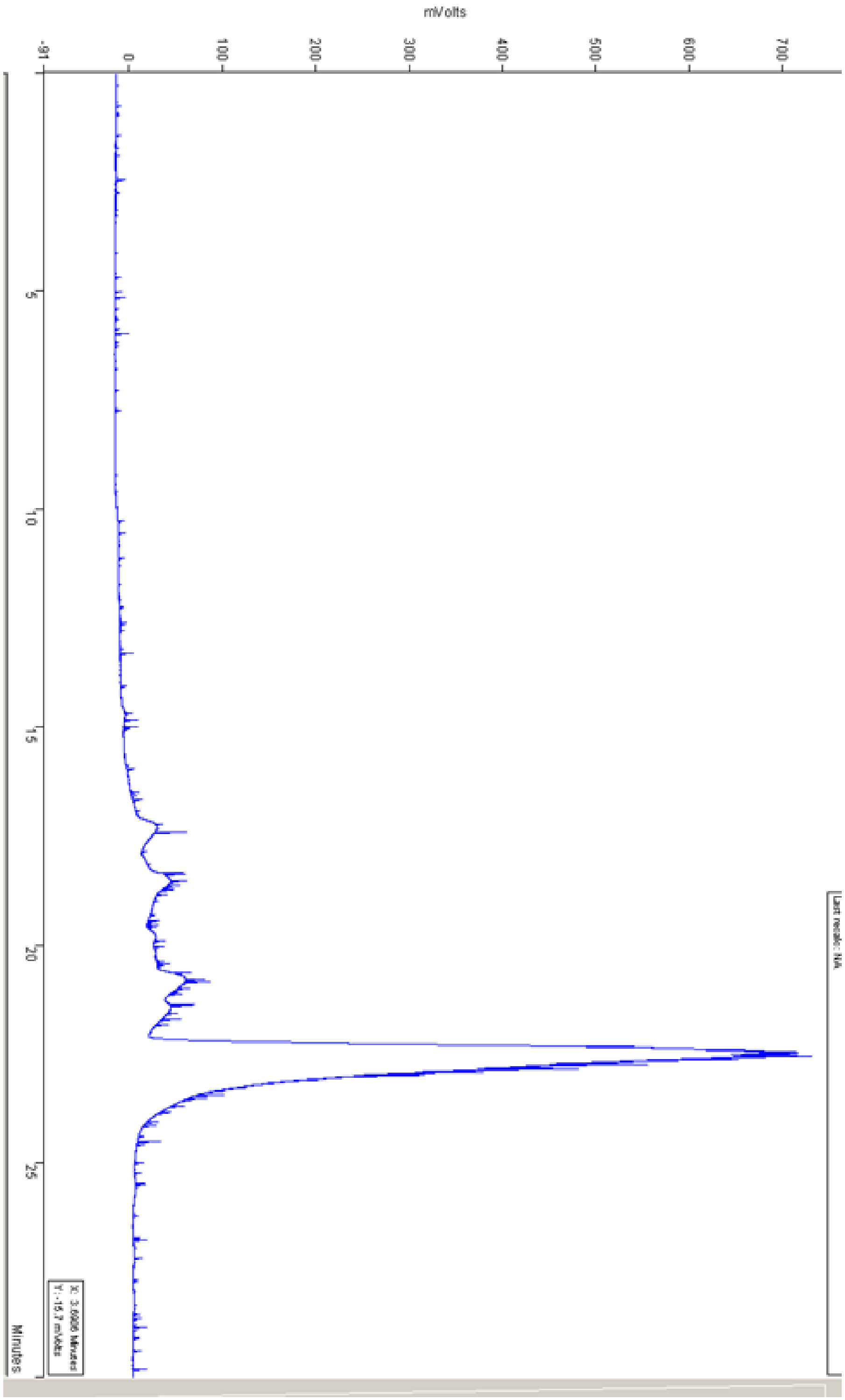

Supplementary Figure 45. RP-HPLC of ADM-116*P*.

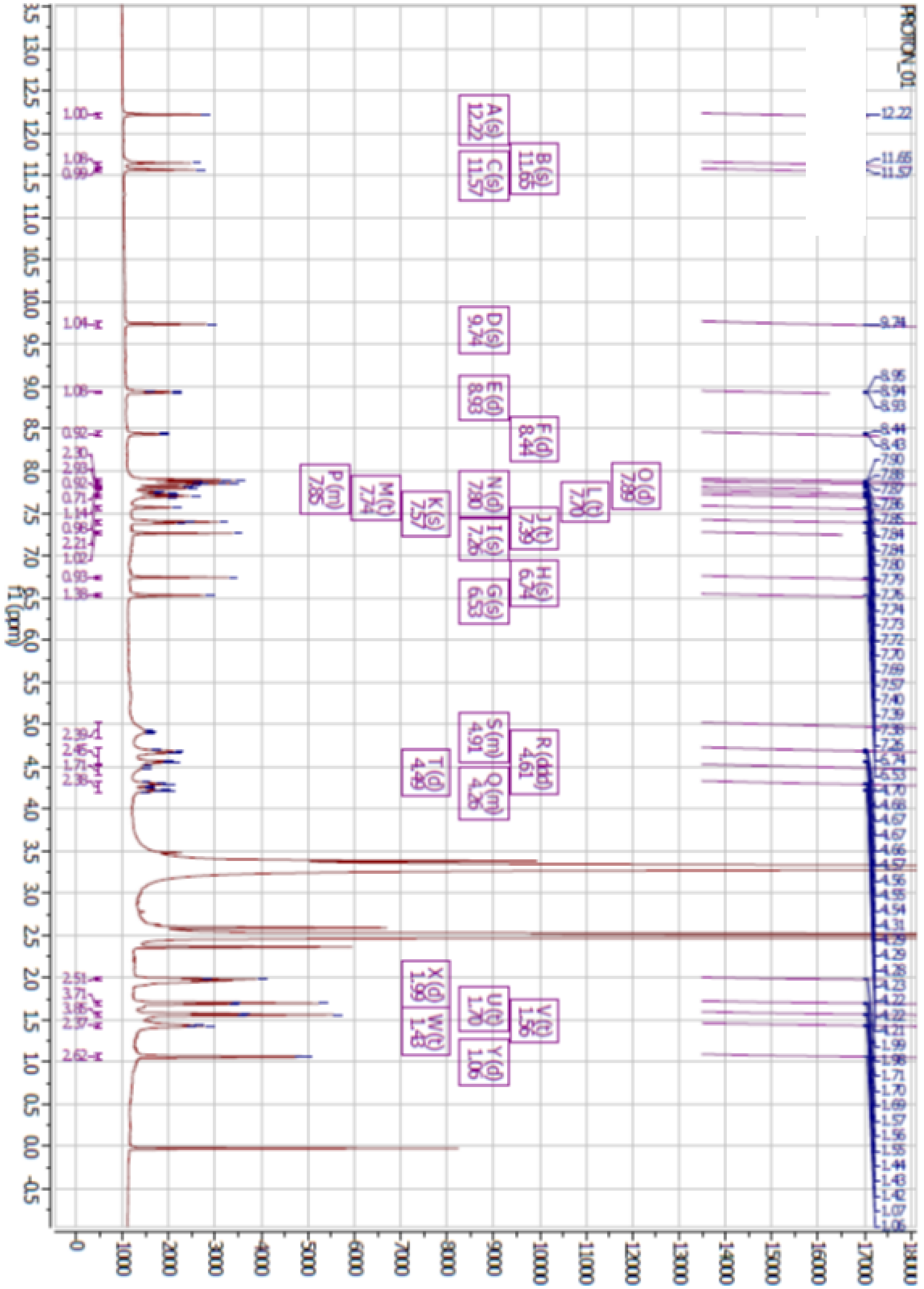

Supplementary Figure 46. ^{1}H-NMR of ADM-116$_M$.

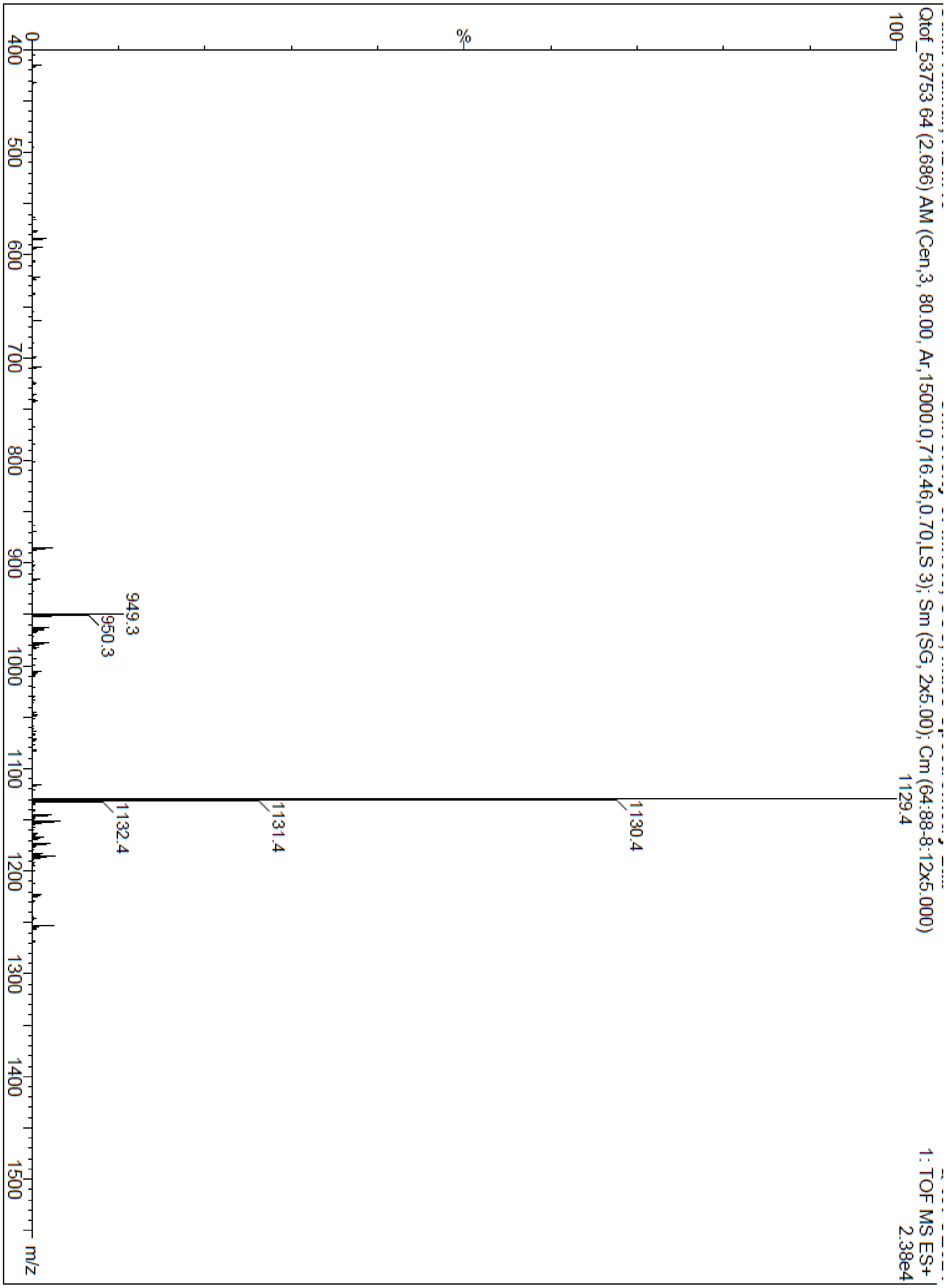

Supplementary Figure 47. ESI-MS of ADM-116$_M$.

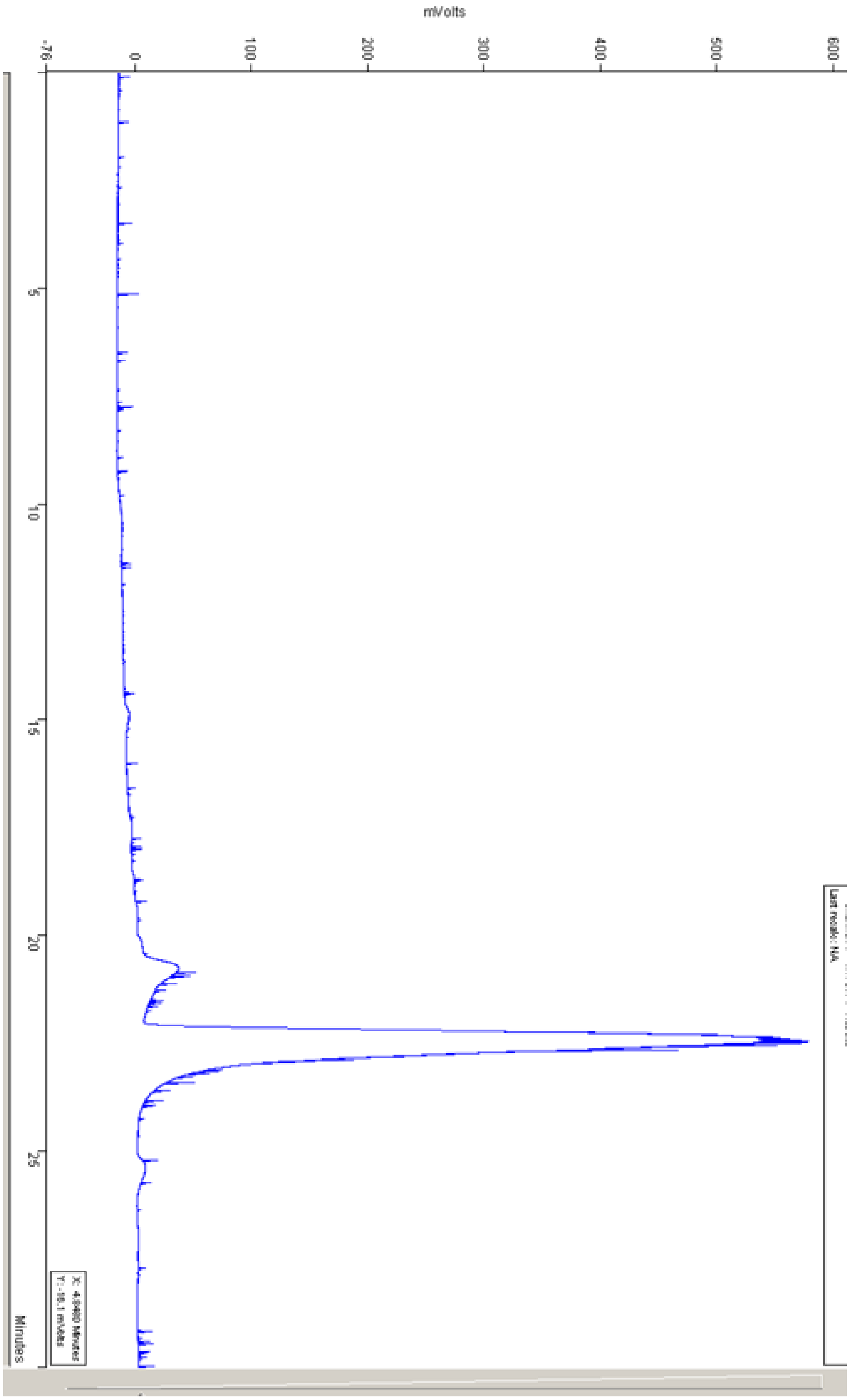

Supplementary Figure 48. RP-HPLC of ADM-116$_M$.

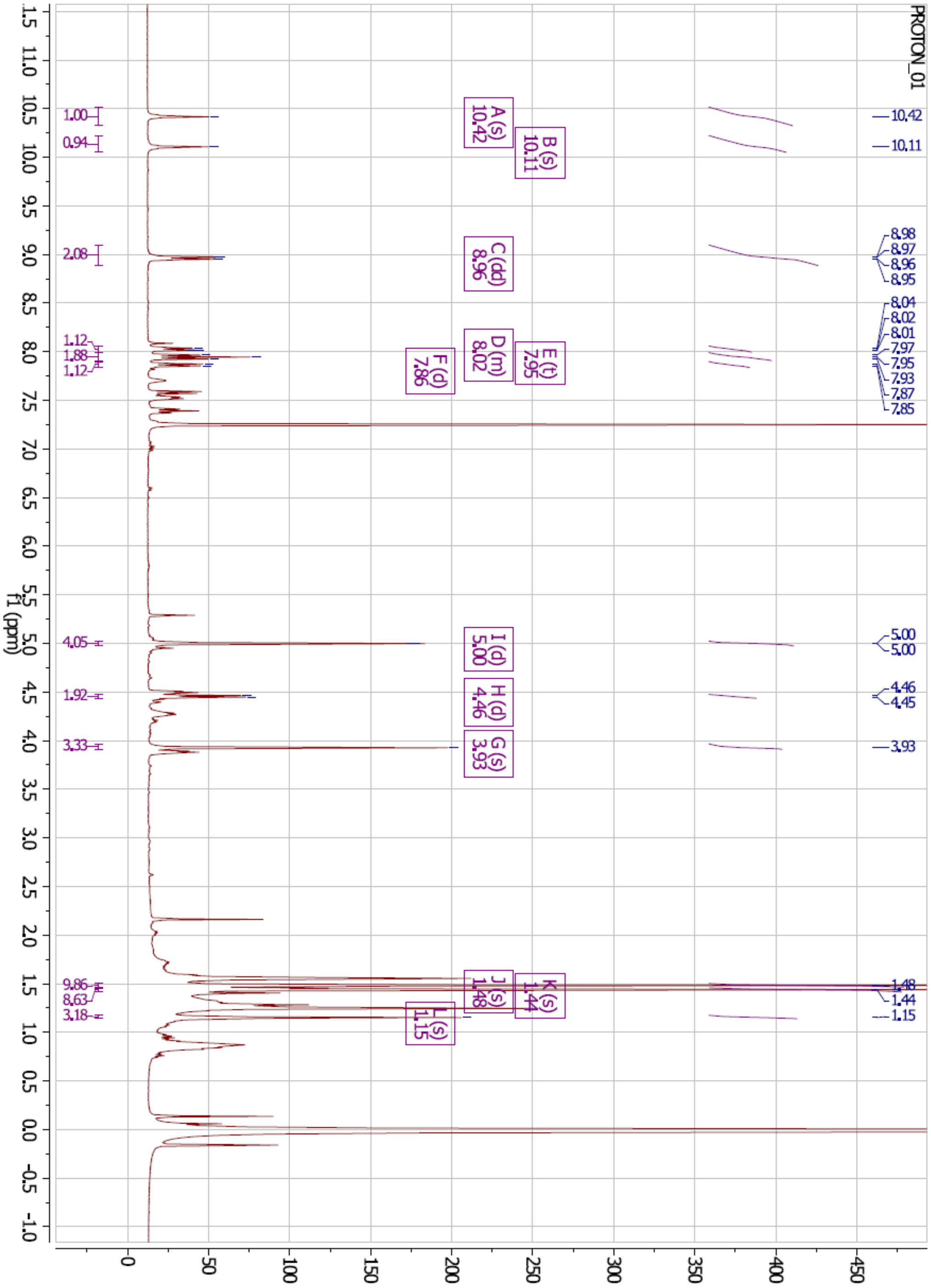

Supplementary Figure 49. ^{1}H-NMR of *tert*-butyl ADM-3-NCS.

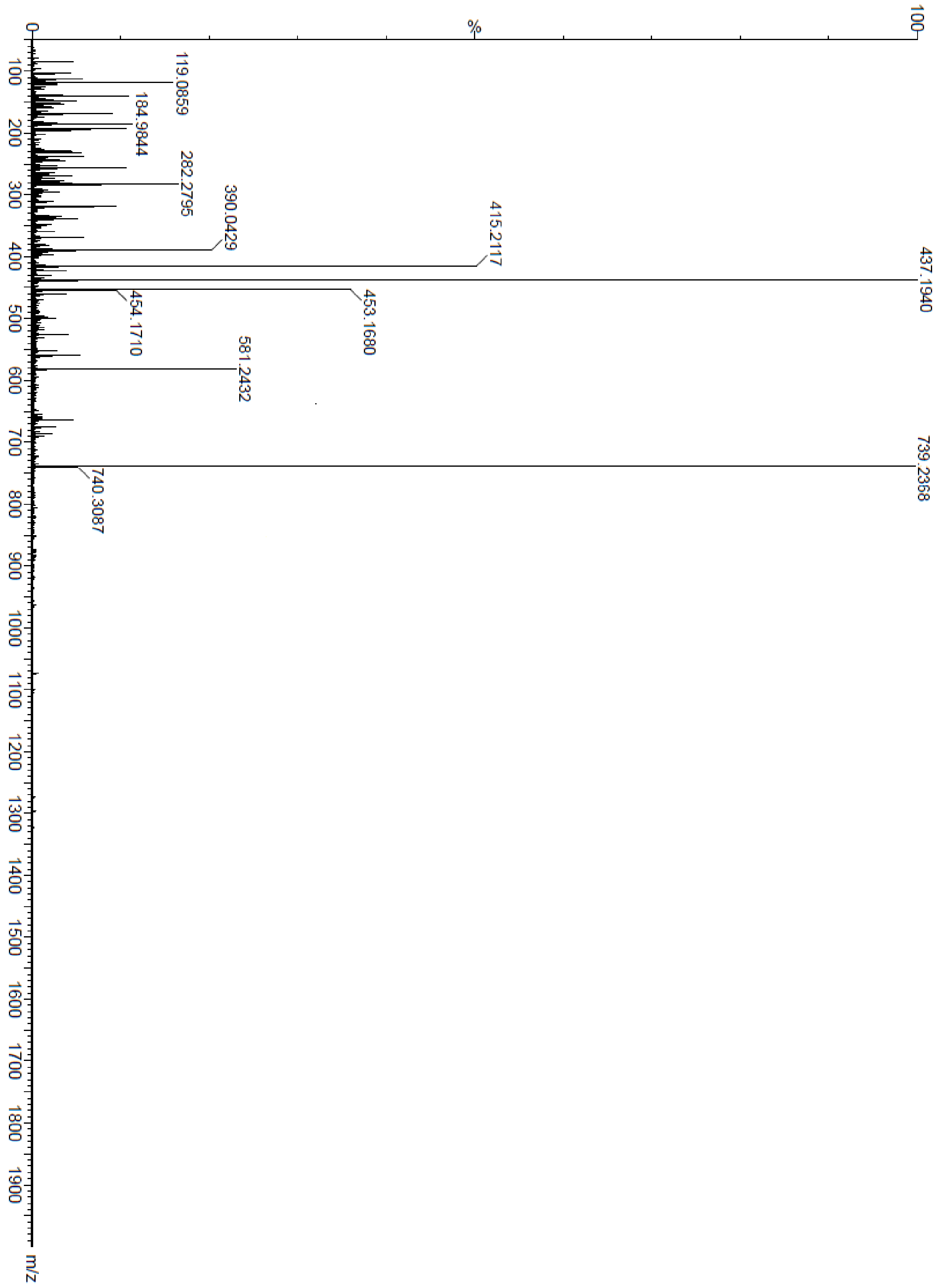

Supplementary Figure 50. ESI-MS of *tert*-butyl ADM-3-NCS.

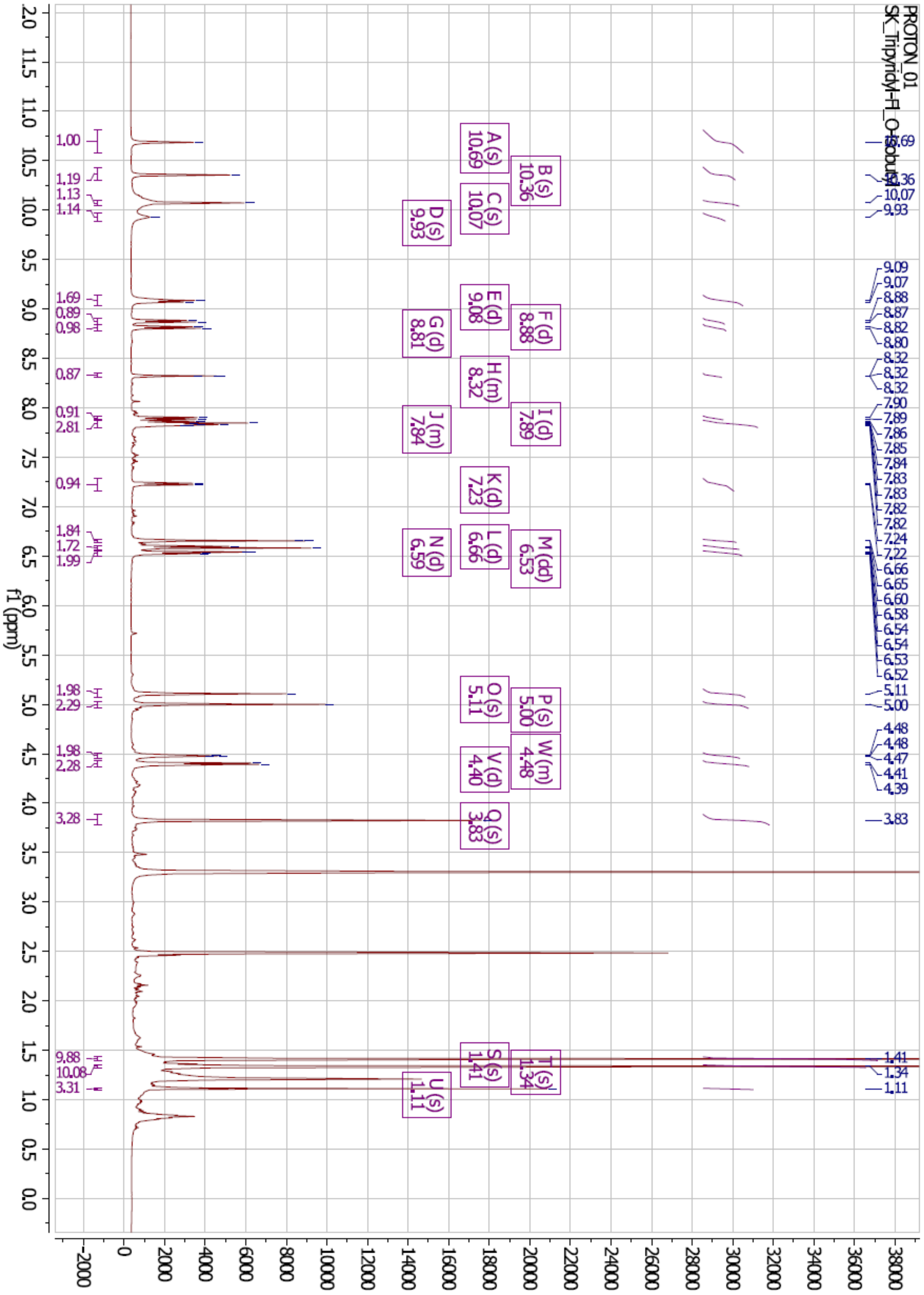

Supplementary Figure 51. ^{1}H-NMR of *tert*-butyl ADM-3_F.

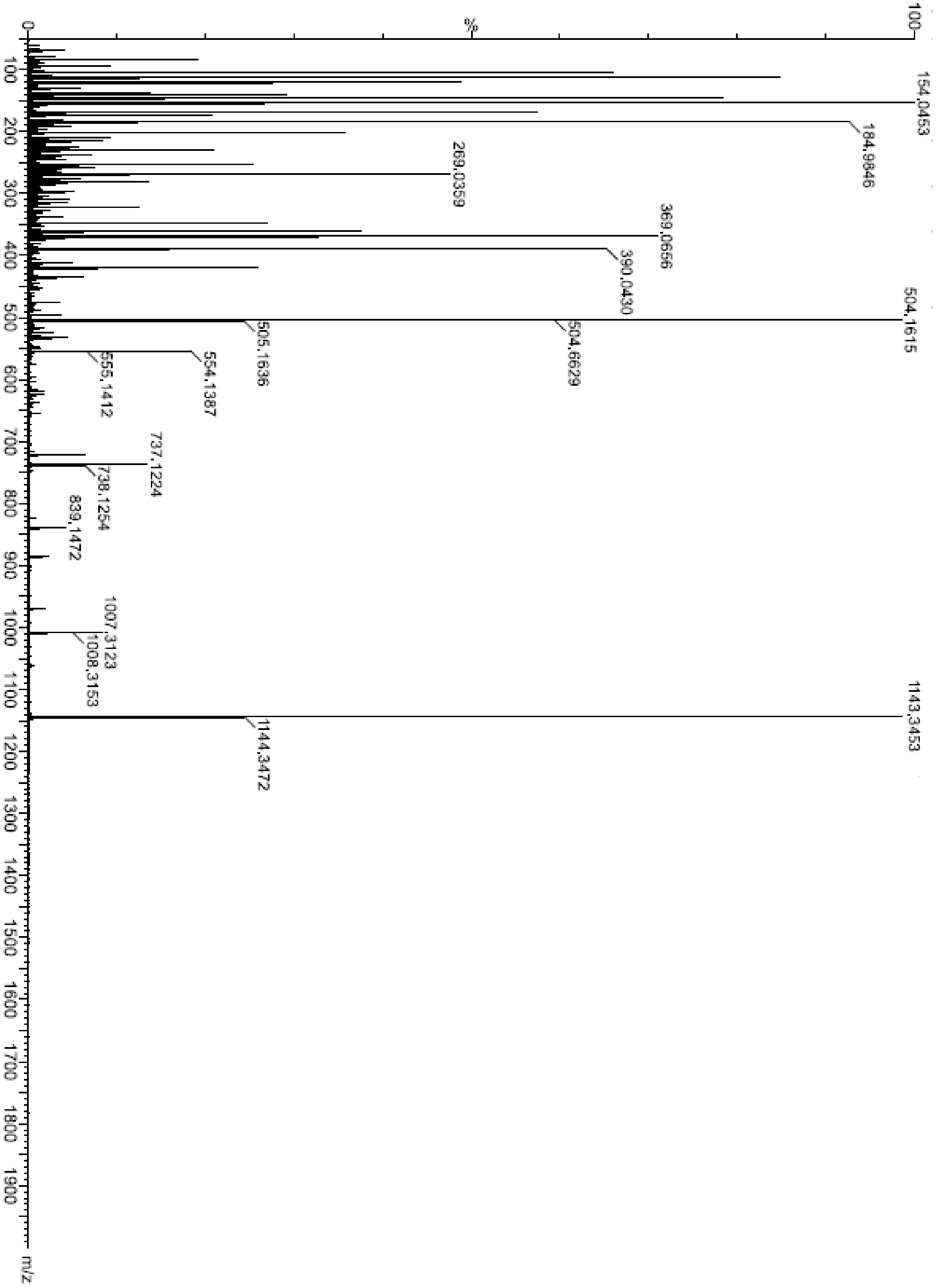

Supplementary Figure 52. ESI-MS of *tert*-butyl ADM-3_F.

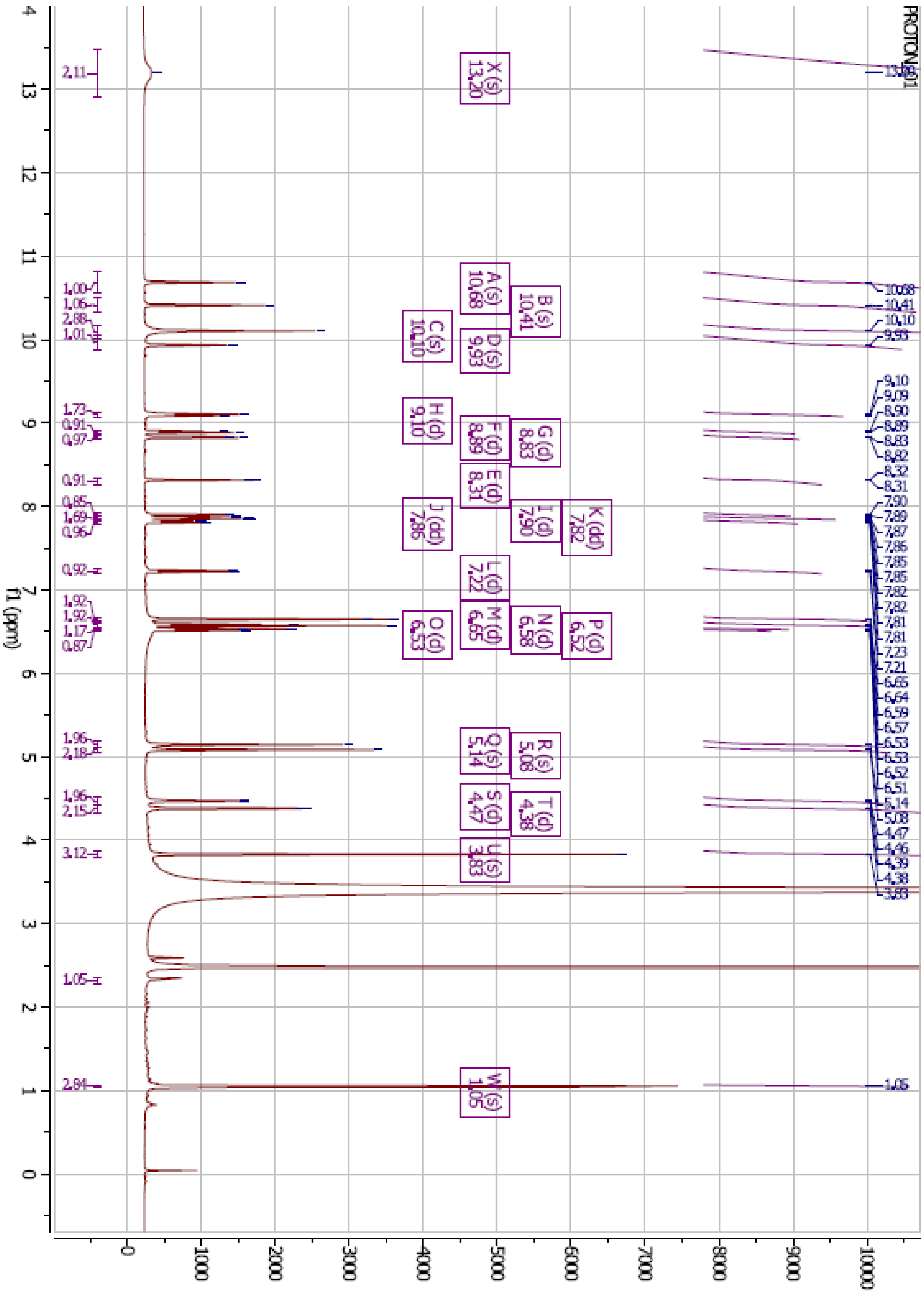

Supplementary Figure 53. ^{1}H-NMR of ADM-3_{F}.

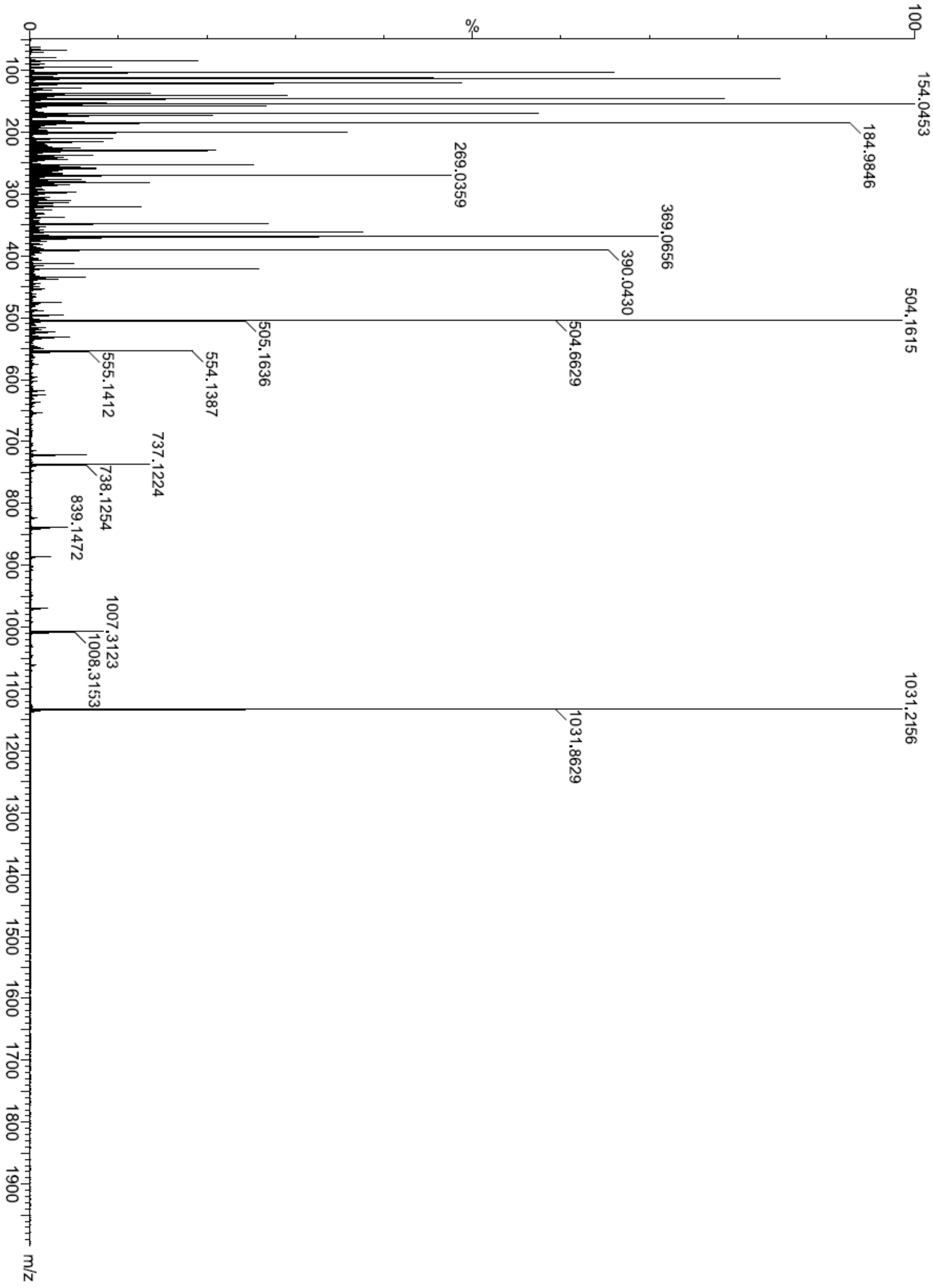

Supplementary Figure 54. ESI-MS of ADM-3_F.

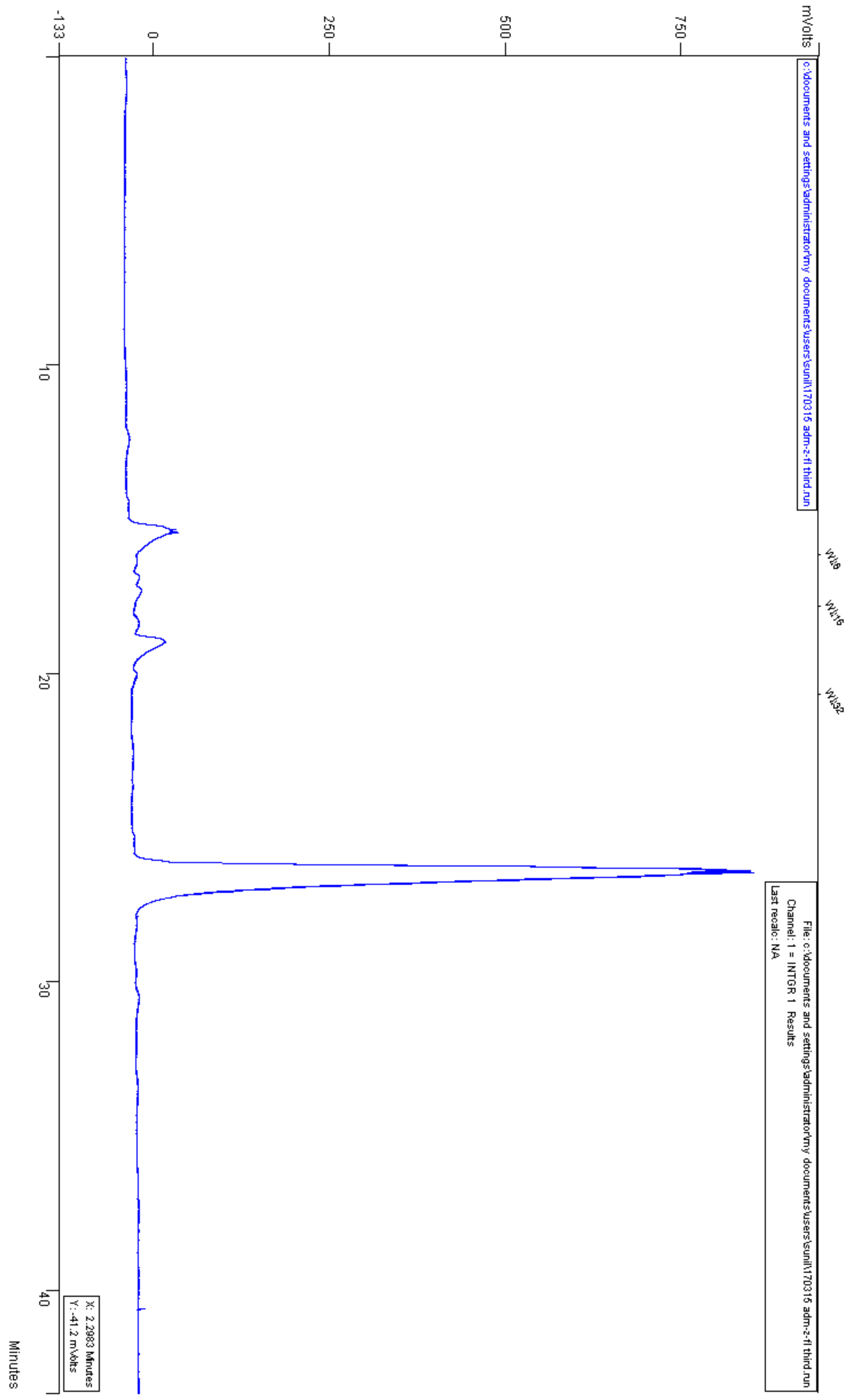

Supplementary Figure 55. RP-HPLC trace of ADM-3_F.

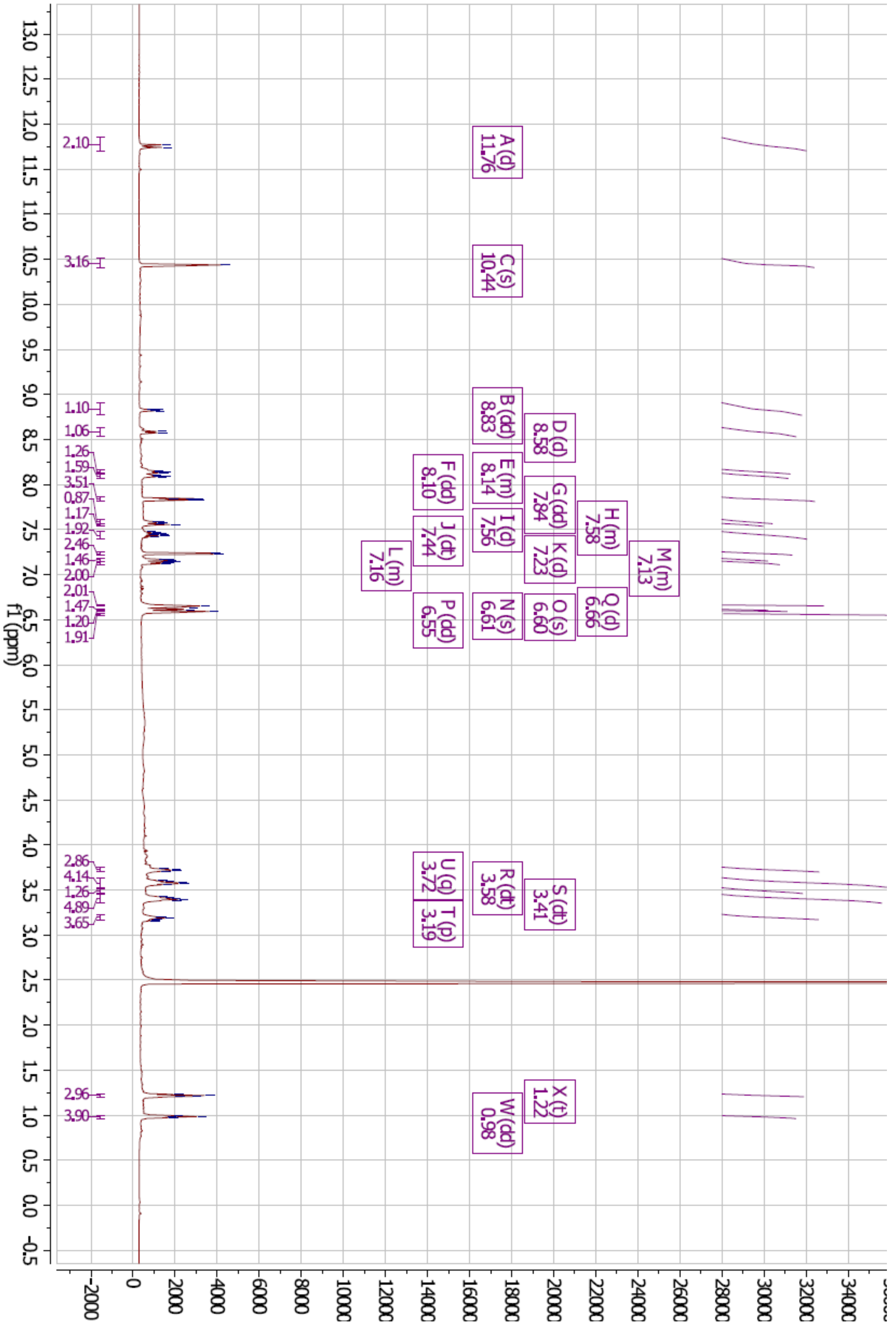

Supplementary Figure 56. ^{1}H-NMR of ADM-116$_{F}$.

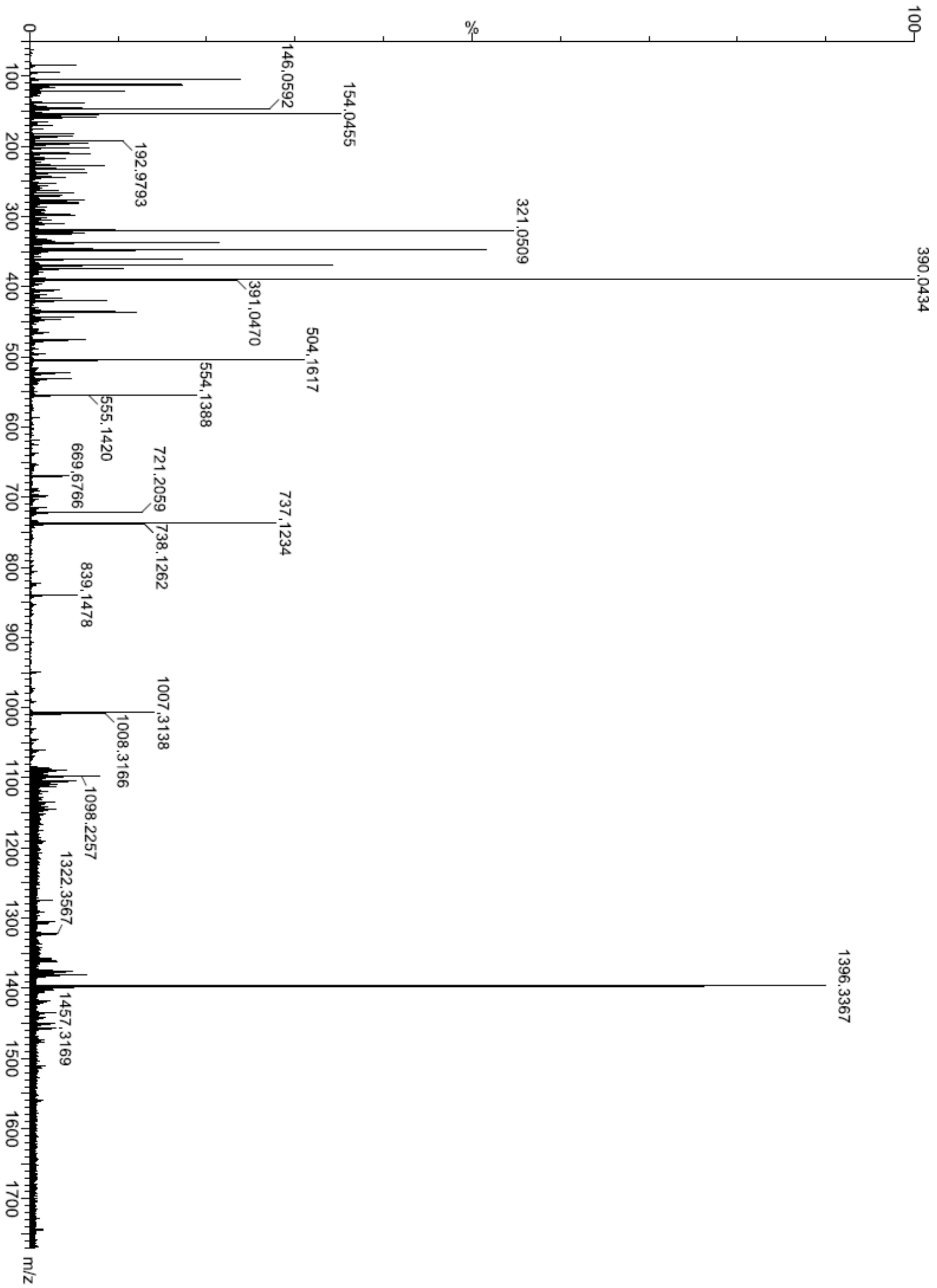

Supplementary Figure 57. ESI-MS of ADM-116$_F$.

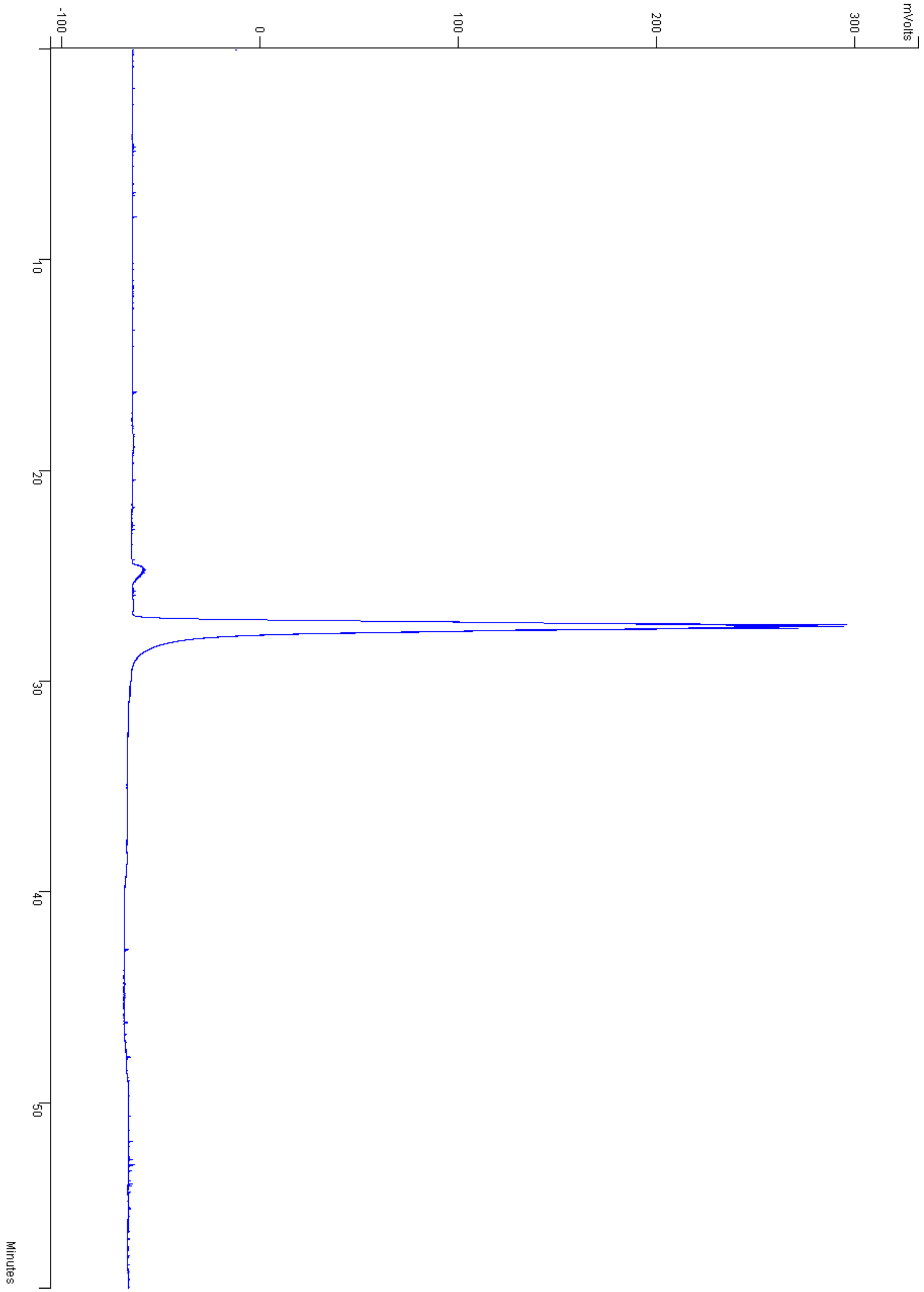

Supplementary Figure 58. RP-HPLC trace of ADM-116$_F$.

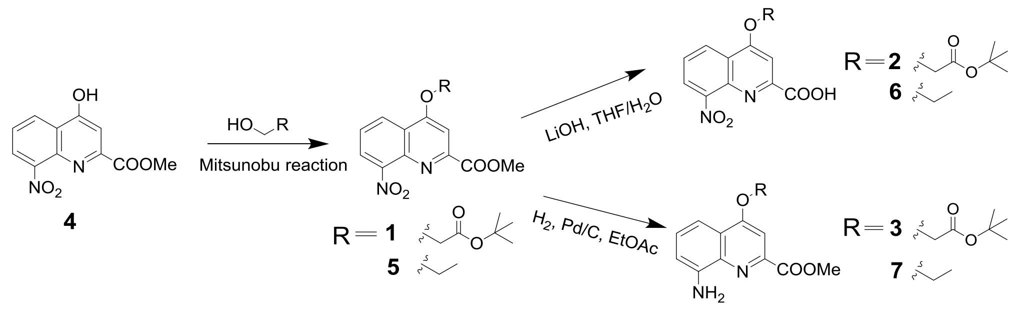

Supplementary Figure 59. Synthesis of monomer quinoline precursors.

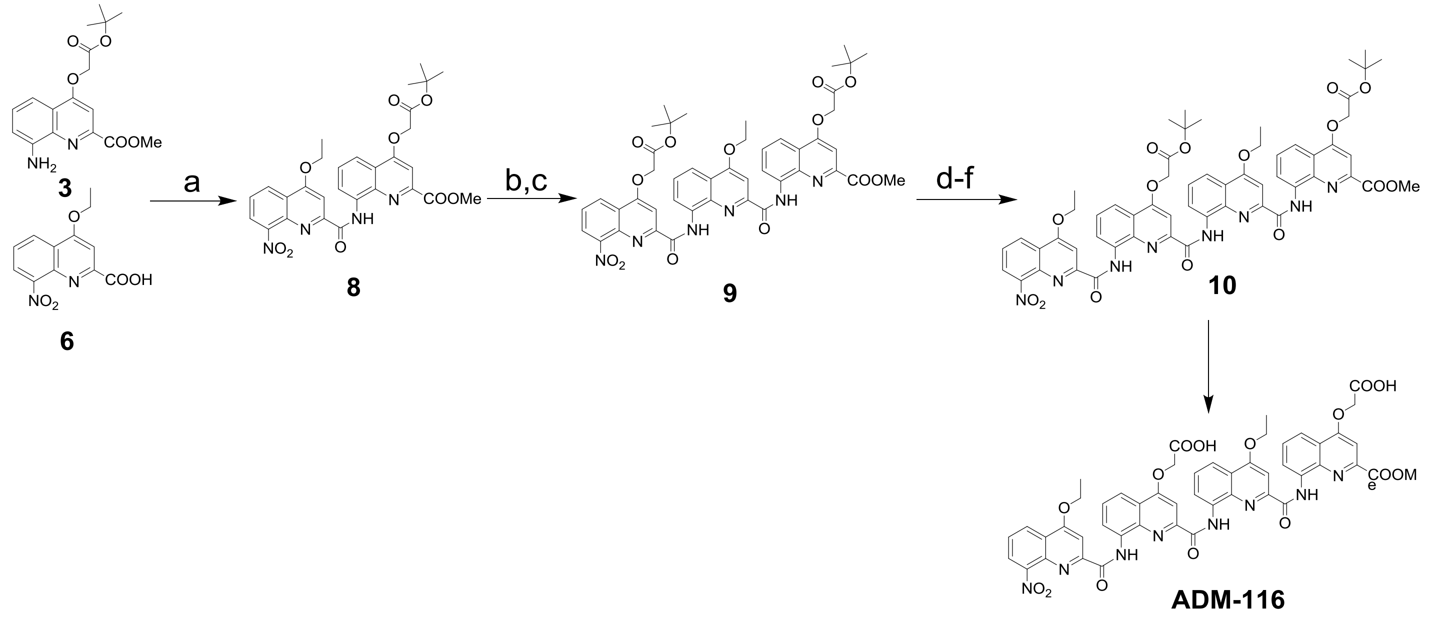

Supplementary Figure 60. Generic representation for the synthesis of oligoquinolines. (a) 2-chloro-1-methylpyridinium iodide, dichloromethane (anhydrous), trimethylamine (anhydrous), 12 h, 50 °C. (b) H_2 (g), Pd/C, ethylacetate, 12 h, r.t. (c) **2**, 2-chloro-1-methylpyridinium iodide, dichloromethane, trimethylamine, 12 h, 50 °C. (d) H_2 (g), Pd/C, ethylacetate, 12 h, r.t. (e) **6**, 2-chloro-1-methylpyridinium iodide, dichloromethane (anhydrous), trimethylamine (anhydrous), 12 h, 50 °C. (f) Triflouroacetic acid, dichloromethane, triethylsilane, 4 h, r.t.

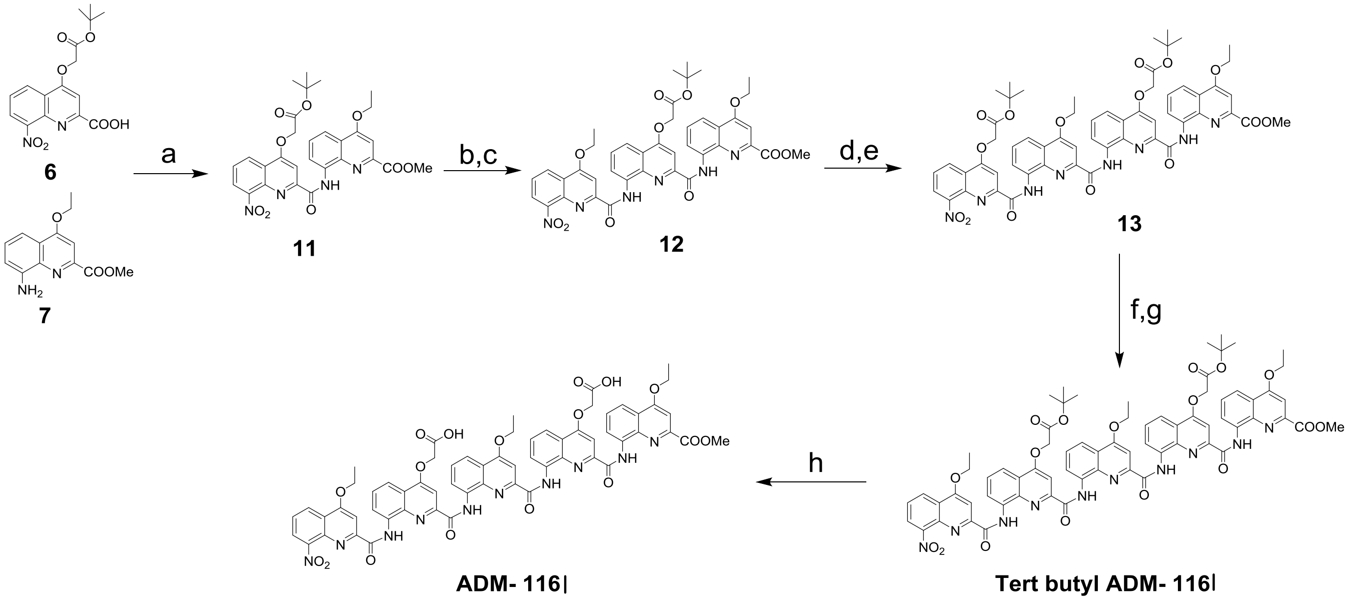

Supplementary Figure 61. Synthesis of ADM-116|. (a) 2-chloro-1-methylpyridinium iodide, dichloromethane (anhydrous), trimethylamine (anhydrous), 12 h, 50 °C. (b) H_2 (g), Pd/C, ethylacetate, 12 h, r.t. (c) **6**, 2-chloro-1-methylpyridinium iodide, dichloromethane, trimethylamine, 12 h, 50 °C. (d) H_2 (g), Pd/C, ethylacetate, 12 h, r.t. (e) **2**, 2-chloro-1-methylpyridinium iodide, dichloromethane (anhydrous), trimethylamine (anhydrous), 12 h, 50 °C. (f) H_2 (g), Pd/C, ethylacetate, 12 h, r.t. (g) **6**, 2-chloro-1-methylpyridinium iodide, dichloromethane (anhydrous), trimethylamine (anhydrous), 12 h, 50 °C. (h) Triflouroacetic acid, dichloromethane, triethylsilane, 4 h, r.t.

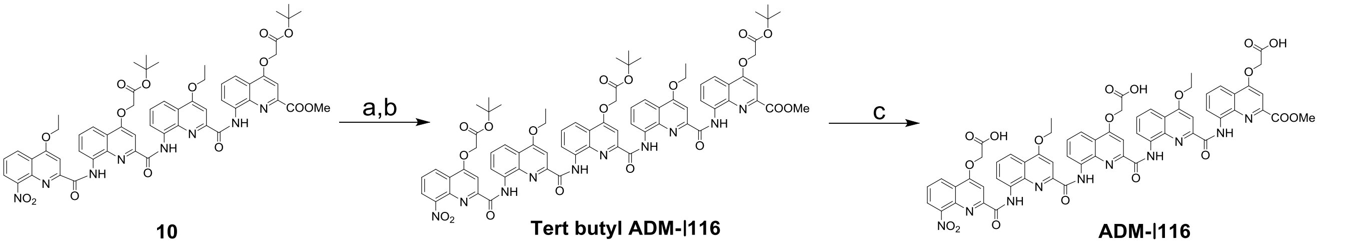

Supplementary Figure 62. Synthesis of ADM-|116. (a) H_2 (g), Pd/C, ethylacetate, 12 h, r.t. (b) **2**, 2-chloro-1-methylpyridinium iodide, dichloromethane (anhydrous), trimethylamine (anhydrous), 12 h, 50 °C. (c) Triflouroacetic acid, dichloromethane, triethylsilane, 4 h, r.t.

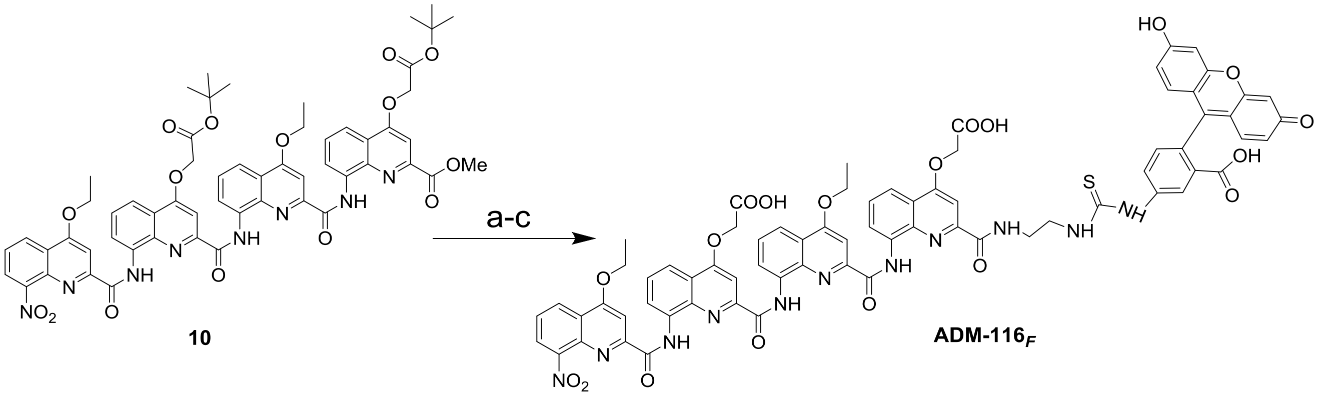

Supplementary Figure 63. Synthesis of ADM-116$_F$. (a) Ethylene diamine, 4-dimethylaminopyridine, 50 °C, 20 h. (b) Triflouroacetic acid, dichloromethane, 4 h, r.t. (c) Fluorescein-5-isothiocyanate, pyridine (anhydrous), N,N-diisopropylethylamine (anhydrous), 20 h, r.t.

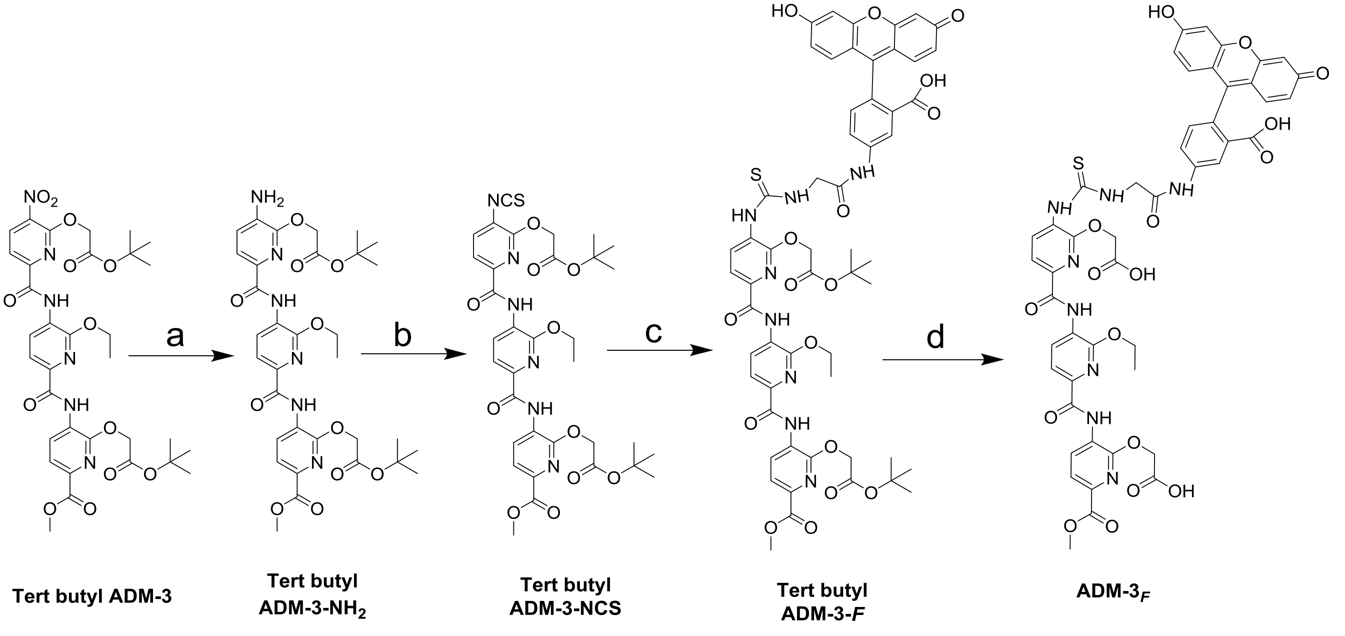

Supplementary Figure 64. Synthesis of ADM-3$_F$. (a) H_2 (g), Pd/C, tetrahydrofuran, 4 h, r.t. (b) 1,1'-thiocarbonyldi-2,2'-pyridone, dichloromethane (anhydrous), 12 h, r.t. (c) 5-(Aminoacetamido)Fluorescein, dichloromethane (anhydrous), N,N-diisopropylethylamine (anhydrous), 20 h, r.t. (d) Triflouroacetic acid, dichloromethane (anhydrous), triethylsilane, 4 h, r.t.

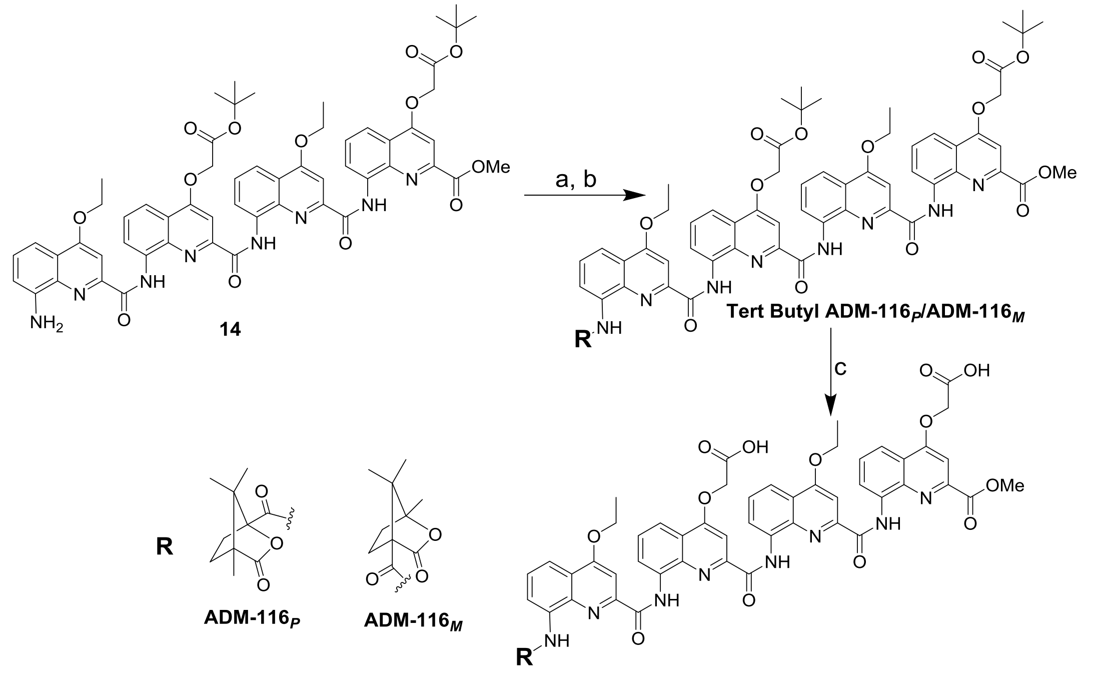

Supplementary Figure 65. Synthesis of ADM-116*P* and ADM-116*M*. (a) H_2 (g), Pd/C, ethylacetate, 12 h, r.t. (b) (1*S*)-(−)-Camphanic chloride or (1*R*)-(+)-Camphanic chloride, trimethylamine (anhydrous), chloroform (anhydrous), 15 min. at 0° C and then 24 h at r.t. (c) Triflouroacetic acid, dichloromethane (anhydrous), triethylsilane, 4 h, r.t.

Supplementary Table 1

**Compound**	**K_a x 10^6 (M^{-1})**	**n**	**ΔH (kJ mol^{-1})**	**ΔS (kJ mol^{-1} K^{-1})**	**TΔS (kJ mol^{-1})**	**ΔG (kJ mol^{-1})**
ADM-116	2.8 ± 0.3	1.0 ± 0.1	-510 ± 120	-1.7 ± 0.3	-470 ± 90	-37 ± 4
ADM-**I**116	0.9 ± 0.2	0.8 ± 0.1	-82 ± 8	-0.17 ± 0.03	-49 ± 9	-33 ± 7
ADM-116*P*	1.1 ± 0.1	0.9 ± 0.1	-1100 ± 70	-3.7 ± 0.2	-1070 ± 70	-34 ± 3

Binding affinities. ITC-derived thermodynamic parameters for the binding of the indicated ligands with IAPP. Presented errors are the standard deviation from experiments performed at least three times.

Supplementary Table 2

Compound	% yield
1	75
2	73
3	92
5	82
6	93
7	96
8	86
9	78
10	71
11	78
12	83
13	71
ADM-116	74
Tert-butyl ADM-ǀ116	69
ADM-ǀ116	72
Tert-butyl ADM-116ǀ	72
ADM-116ǀ	66
Tert-butyl ADM-116$_P$	62
ADM-116$_P$	67
Tert-butyl ADM-116$_M$	64
ADM-116$_M$	68

ADM-116$_F$	25% (overall yield for three steps)
Tert-butyl ADM-3-NH_2	98%
Tert-butyl ADM-3-NCS	91%
Tert-butyl ADM-3$_F$	76%
ADM-3$_F$	72%

% yield. A combined % yield for the monomeric precursors and the oligomers of oligoquinolines used in this study.

Supplementary Methods

Synthesis of compounds 1, 2, 3, and 4 is reported elsewhere[1].

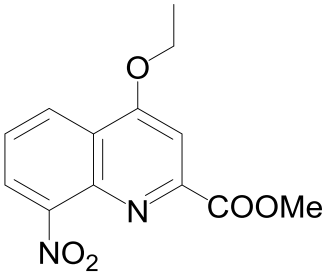

To a solution of 4 (5 g, 20 mmol) in acetone/ dimethylformamide (160 mL, 15:1 v/v), sodium carbonate (4.1 g, 30 mmol) was added followed by ethyl iodide (3.2 mL, 40 mmol) under an inert atmosphere. The solution was stirred overnight at 70 °C under an inert atmosphere. The volatiles were removed on rotovap and the reaction mixture was dissolved in ethylacetate (300 mL) and water (300 mL). The water layer was washed with ethylacetate (2 × 100 mL). The organic solutions were collected and successively washed (2 × each) with water (20 mL), 0.5 M solution of HCl (20 mL), and with brine (20 mL), dried over sodium sulfate and evaporated under vacuum to afford 5 as a pale yellow solid (3.3 g, 60%) which was recrystallised in $CHCl_3$/ MeOH (120, 1:4 v/v). TLC (Hexane:Ethylacetate, 70:30 v/v): R_F=0.7; ^{1}H NMR (400 MHz, Chloroform-*d*) δ 8.53 – 8.40 (dd, *J* = 8.5, 1.4 Hz, 1H), 8.19 – 8.04 (dd, *J* = 7.5, 1.4 Hz, 1H), 7.68 – 7.66 (s, 1H), 7.65 – 7.61 (m, 1H), 4.45 – 4.35 (q, *J* = 7.0 Hz, 2H), 4.06 – 4.01 (s, 3H), 1.65 – 1.59 (t, *J* = 7.0 Hz, 4H). MS (MALDI-TOF) calcd for $C_{13}H_{12}N_2O_5$ (M+H+), 276.25, obsd: 276.58.

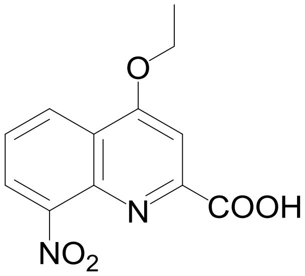

To a solution of **5** (2.0 g, 7.2 mmol) in tetrahydrofuran (100 mL), lithium hydroxide (0.2 N) in water (55 mL) was adeed and the solution was stirred for 45 min. Acetic acid (conc.) was then added with constant stirring to bring down the pH to ~4. To this reaction mixture, dichloromethane (200 mL) was added and the organic layer was successively washed once with water (20 mL) and brine (20 mL), dried over sodium sulfate, filtered, and evaporated to afford compound 6 as a yellow solid (Table 1 for % yield).

^{1}H NMR (400 MHz, Chloroform-*d*) δ 8.59 – 8.51 (dd, *J* = 8.5, 1.4 Hz, 1H), 8.26 – 8.20 (dd, *J* = 7.5, 1.4 Hz, 1H), 7.76 – 7.69 (m, 2H), 4.50 – 4.41 (q, *J* = 7.0 Hz, 2H), 1.68 – 1.62 (t, *J* = 7.0 Hz, 3H). MS (MALDI-TOF) (m/z): calculated for $C_{12}H_{10}N_2O_5$ [(M+H)+]: 262.22, found 262.70.

Standard protocol for reduction of nitro-oligoquinolines

To a solution of nitroquinoline (0.1-0.5 mmol) in ethylacetate (10 mL), Pd/C (10% wt.) was added and the reaction started with constant stirring at r. t. in the atmosphere of H_2 (g). The progress of the reaction was monitored by TLC. Generally, the reduction reactions were completed in 12 h. The reaction mixture was filtered and dried which result in a yellow solid with quantitative yield. The product was used in the next step without further characterisation.

Standard protocol for amide coupling

To a solution of COOH nitroquinoline (0.12 mmol) in anhydrous dichloromethane (10 mL), triethylamine (0.4 mmol, anhydrous) and 2-chloromethyl-1-methyl pyridinium iodide (0.12 mmol) were added and the reaction was refluxed for 20 min. at 50 °C under inert atmosphere. To this solution, amino-oligoquinoline (0.1 mmol) was added and reaction started with constant stirring at 50 °C under inert atmosphere. The reaction mixture was stirred for 12 h after which the volatiles were removed on rotovap. Flash chromatography (0 to 35% ethylacetate in hexane, v/v) yielded the desired product as a yellow to brown solid.

Standard protocol for deprotection of oligoquinolines

To a solution of oligoquinoline (0.04 mmol), in dichloromethane (2 mL), triethylsilane (0.1 mL, excess) and trifluoroacetic acid (0.5 mL, excess) were added and the reaction solution was stirred at r.t. for 4 h. The reaction mixture was dried and washed with cold diethyl ether (4 × 3 mL) which resulted in a yellow to brown solid.

***Tert*-butyl ADM-3**

Synthsis and characterisation of *tert*-butyl ADM-3 was reported elsewhere[2].

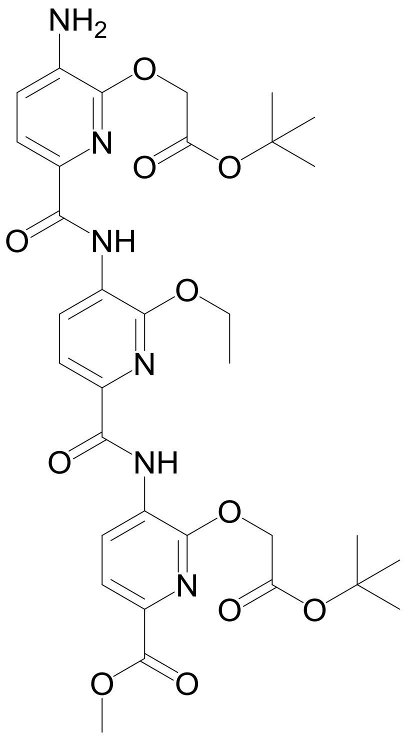

To a solution of *tert*-butyl ADM-3 (30 mg, 0.04 mmol) in tetrahydrofuran (5 mL), Pd/C (5 mg) was added and the reaction started with constant stirring in the atmosphere of H_2 (g) at r. t. The progress of the reaction was monitored using TLC. The disappearance of the starting material confirms the completion of the reaction (after 6 h). The reaction mixture was filtered and the filterate was dried over rotovap to afford the desired product as a yellow solid (26.5 mg, 95%). TLC (Hexane:Ethylacetate, 60:40 v/v): R_F=0.55.

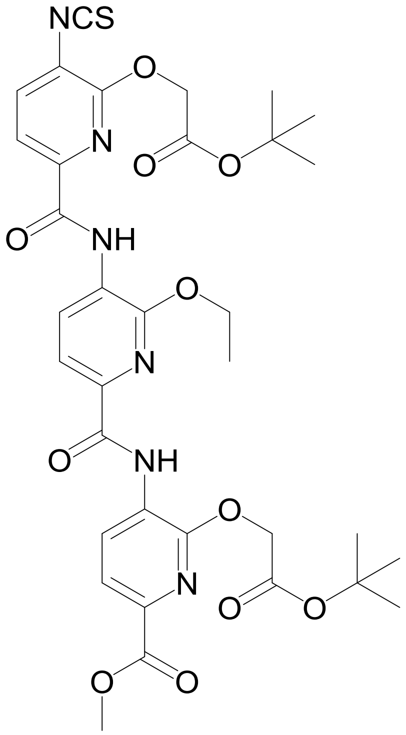

To a solution of *tert*-butyl ADM-3-NH_2 (25 mg, 0.036 mmol) in dichloromethane (5 mL, anhydrous), 1,1' thiocarbonyldi-2,2'-pyridone (10 mg, 0.043 mmol, 1.2 eq.,) was added and the reaction was started with continuous stirring under inert atmosphere. The reaction solution was stirred overnight and then the product was purified using column charomatography (24 mg, 91%). TLC (Hexane:Ethylacetate, 70:30 v/v): R_F=0.76; ^{1}H NMR (400 MHz, Chloroform-*d*) δ 10.52 – 10.33 (s, 1H), 10.22 – 10.05 (s, 1H), 9.10 – 8.89 (dd, *J* = 8.2, 2.7 Hz, 2H), 8.06 – 8.00 (m, 1H), 7.99 – 7.91 (t, *J* = 8.0 Hz, 2H), 7.90 – 7.84 (d, *J* = 8.1 Hz, 1H), 5.02 – 4.97 (d, *J* = 2.0 Hz, 4H), 4.48 – 4.43 (d, *J* = 6.8 Hz, 2H), 3.97 – 3.91 (s, 3H), 1.50 – 1.46 (s, 10H), 1.46 – 1.41 (s, 9H), 1.17 – 1.14 (s, 3H). MS (MALDI-TOF) calcd for $C_{34}H_{39}N_6O_{11}S$ ($M+H^+$), 739.2398, obsd: 739.2368.

TLC (dichloromethane:methanol:triethylamine, 83:17:0.5, v/v/v): R_F=0.6; ^{1}H NMR (500 MHz, DMSO-d_6) δ 10.81 – 10.58 (s, 1H), 10.43 – 10.31 (s, 1H), 10.10 – 10.04 (s, 1H), 9.97 – 9.89 (s, 1H), 9.14 – 9.03 (d, *J* = 8.3 Hz, 2H), 8.90 – 8.85 (d, *J* = 8.0 Hz, 1H), 8.84 – 8.78 (d, *J* = 8.1 Hz, 1H), 8.35 – 8.31 (m, 1H), 7.92 – 7.88 (d, *J* = 8.0 Hz, 1H), 7.87 – 7.80 (m, 3H), 7.28 – 7.16 (d, *J* = 8.3 Hz, 1H), 6.67 – 6.64 (d, *J* = 2.3 Hz, 2H), 6.61 – 6.57 (d, *J* = 8.7 Hz, 2H), 6.55 – 6.50 (dd, *J* = 8.7, 2.4 Hz, 2H), 5.15 – 5.07 (s, 2H), 5.03 – 4.96 (s, 2H), 4.51 – 4.45 (m, 2H), 4.43 – 4.37 (d, *J* = 6.8 Hz, 2H), 3.89 – 3.78 (s, 3H), 1.43 – 1.39 (s, 9H), 1.35 – 1.32 (s, 9H), 1.12 – 1.10 (s, 3H). MS (MALDI-TOF) calcd for $C_{56}H_{55}N_8O_{17}S$ (M+H$^+$), 1143.3406, obsd: 1143.3453.

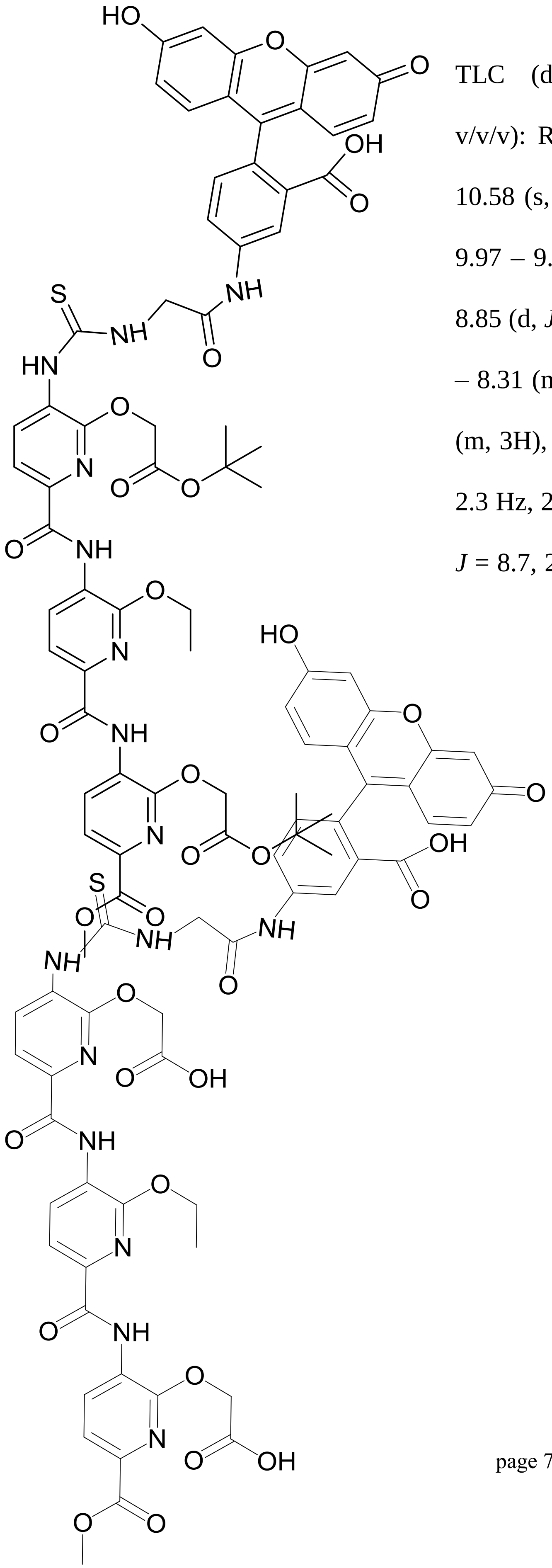

ADM-3*F*

^{1}H NMR (600 MHz, DMSO-d_6) δ 13.47 – 12.91 (s, 2H), 10.81 – 10.56 (s, 1H), 10.50 – 10.32 (s, 1H), 10.17 – 10.01 (s, 3H), 10.04 – 9.88 (s, 1H), 9.13 – 9.07 (d, J = 8.4 Hz, 2H), 8.92 – 8.87 (d, J = 7.9 Hz, 1H), 8.85 – 8.80 (d, J = 8.0 Hz, 1H), 8.34 – 8.26 (d, J = 1.9 Hz, 1H), 7.92 – 7.88 (d, J = 8.0 Hz, 1H), 7.88 – 7.84 (dd, J = 8.1, 4.0 Hz, 2H), 7.83 – 7.79 (dd, J = 8.4, 2.0 Hz, 1H), 7.26 – 7.19 (d, J = 8.3 Hz, 1H), 6.67 – 6.62 (d, J = 2.3 Hz, 2H), 6.61 – 6.56 (d, J = 8.6 Hz, 2H), 6.54 – 6.52 (d, J = 2.3 Hz, 1H), 6.52 – 6.50 (d, J = 2.4 Hz, 1H), 5.19 – 5.11 (s, 2H), 5.12 – 5.05 (s, 2H), 4.51 – 4.43 (d, J = 4.8 Hz, 2H), 4.42 – 4.32 (d, J = 6.9 Hz, 2H), 3.87 – 3.78 (s, 3H), 1.06 – 1.04 (s, 3H). MS (MALDI-TOF) calcd for $C_{48}H_{39}N_8O_{17}S$ (M+H$^+$), 1031.2154, obsd: 1031.2156.

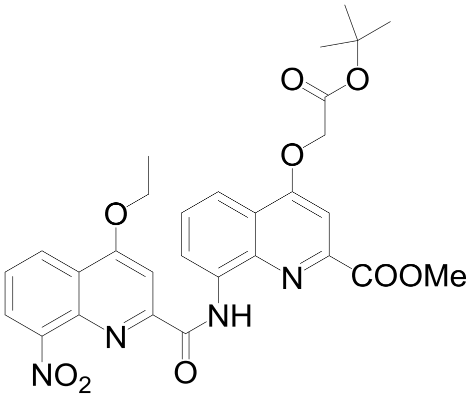

TLC (Hexane:Ethylacetate, 60:40 v/v): R_F=0.62; ^{1}H NMR (400 MHz, Chloroform-d) δ 11.87 – 11.79 (s, 1H), 9.13 – 9.04 (dd, J = 7.7, 1.2 Hz, 1H), 8.51 – 8.45 (dd, J = 8.3, 1.3 Hz, 1H), 8.19 – 8.13 (dd, J = 7.5, 1.4 Hz, 1H), 8.11 – 8.03 (dd, J = 8.4, 1.3 Hz, 1H), 7.70 – 7.58 (dt, J = 14.4, 8.0 Hz, 2H), 7.55 – 7.51 (s, 1H), 7.42 – 7.34 (t, J = 7.8 Hz, 1H), 4.88 – 4.78 (s, 2H), 4.48 – 4.36 (q, J = 7.3 Hz, 2H), 4.25 – 4.16 (s, 3H), 1.64 – 1.58 (t, J = 7.0 Hz, 3H), 1.53 – 1.48 (s, 9H). MS (MALDI-TOF) calcd for $C_{29}H_{28}N_4O_9$ (M+H$^+$), 576.18, obsd: 576.03.

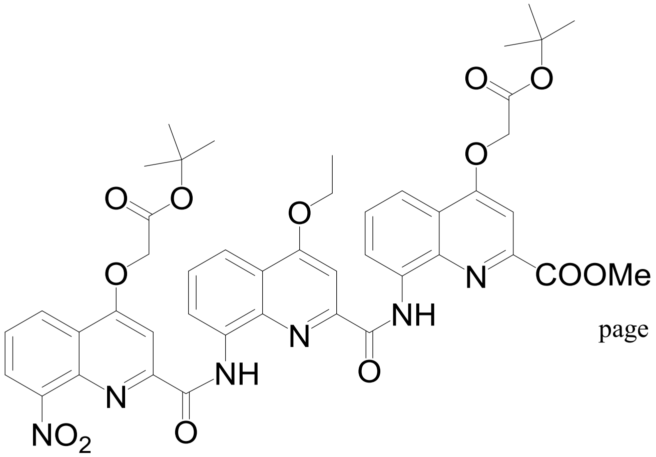

TLC (Hexane:Ethylacetate, 60:40 v/v): R_F=0.55; ^{1}H NMR (400 MHz, Chloroform-d) δ 12.26 – 12.21 (s, 1H), 12.18 – 12.13 (s, 1H), 9.09 – 8.98 (ddd, J = 13.8, 7.7, 1.3 Hz, 2H), 8.49 – 8.41 (dd, J

= 8.4, 1.5 Hz, 1H), 8.18 – 8.11 (dd, *J* = 8.4, 1.3 Hz, 1H), 8.06 – 7.97 (dd, *J* = 8.4, 1.3 Hz, 1H), 7.95 – 7.90 (s, 1H), 7.82 – 7.67 (m, 3H), 7.64 – 7.56 (dd, *J* = 7.5, 1.5 Hz, 1H), 7.48 – 7.35 (m, 1H), 6.72 – 6.67 (s, 1H), 4.96 – 4.89 (s, 2H), 4.65 – 4.60 (s, 2H), 4.57 – 4.47 (q, *J* = 6.9 Hz, 2H), 3.47 – 3.41 (s, 3H), 1.74 – 1.64 (t, *J* = 7.0 Hz, 3H), 1.68 – 1.53 (m, 18H). MS (MALDI-TOF) calcd for $C_{45}H_{44}N_6O_{13}$ ($M+H^+$), 876.88, obsd: 875.26.

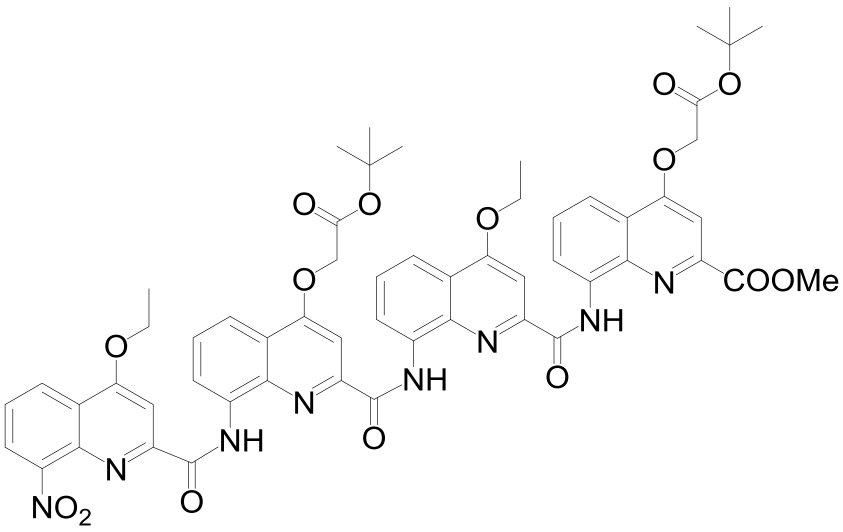

TLC (Hexane:Ethylacetate, 60:40 v/v): R_F=0.42; ^{1}H NMR (400 MHz, Chloroform-*d*) δ 12.34 – 12.26 (s, 1H), 11.91 – 11.84 (s, 1H), 11.69 – 11.61 (s, 1H), 9.15 – 9.08 (dd, *J* = 7.6, 1.2 Hz, 1H), 8.61 – 8.52 (dd, *J* = 8.4, 1.4 Hz, 1H), 8.44 – 8.35 (dd, *J* = 7.7, 1.2 Hz, 1H), 8.23 – 8.15 (dd, *J* = 7.7, 1.2 Hz, 1H), 8.12 – 8.06 (dd, *J* = 8.4, 1.2 Hz, 1H), 8.04 – 7.95 (ddd, *J* = 8.6, 6.2, 1.2 Hz, 2H), 7.77 – 7.76 (s, 1H), 7.75 – 7.70 (t, *J* = 8.1 Hz, 1H), 7.65 – 7.57 (m, 2H), 7.48 – 7.44 (s, 1H), 7.42 – 7.32 (m, 2H), 6.89 – 6.85 (s, 1H), 6.64 – 6.59 (s, 1H), 4.99 – 4.87 (s, 2H), 4.73 – 4.58 (m, 4H), 4.26 – 4.14 (d, *J* = 7.5 Hz, 2H), 3.49 – 3.37 (s, 3H), 1.80 – 1.73 (t, *J* = 7.0 Hz, 3H), 1.61 – 1.58 (s, 9H), 1.60-1.55 (m, 3H), 1.53 – 1.50 (s, 9H). MS (MALDI-TOF) calcd for $C_{57}H_{54}N_8O_{15}$ ($M+H^+$), 1191.10, obsd: 1190.91.

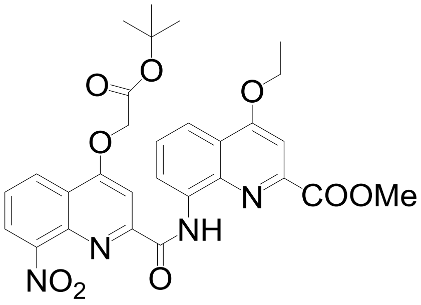

TLC (Hexane:Ethylacetate, 70:30 v/v): R_F=0.60; ^{1}H NMR (400 MHz, Chloroform-*d*) δ 11.93 – 11.80 (s, 1H), 9.10 – 9.03 (dd, *J* = 7.8, 1.3 Hz, 1H), 8.67 – 8.58 (dd, *J* = 8.4, 1.4 Hz, 1H), 8.27 – 8.17 (dd, *J* = 7.5, 1.4 Hz, 1H), 8.04 – 7.98 (dd, *J* = 8.4, 1.3 Hz, 1H), 7.89 – 7.86 (s, 1H), 7.73 – 7.66 (dd, *J* = 8.5, 7.5 Hz, 1H), 7.66 – 7.60 (m, 2H), 4.98 – 4.89 (s, 2H), 4.44 – 4.33 (q, *J* = 7.0 Hz, 2H), 4.24 – 4.18 (s, 3H), 1.64 – 1.57 (t, *J* = 7.0 Hz, 3H), 1.54 – 1.53 (s, 9H). MS (MALDI-TOF) calcd for $C_{29}H_{28}N_4O_9$ (M+H$^+$), 576.18, obsd: 576.12.

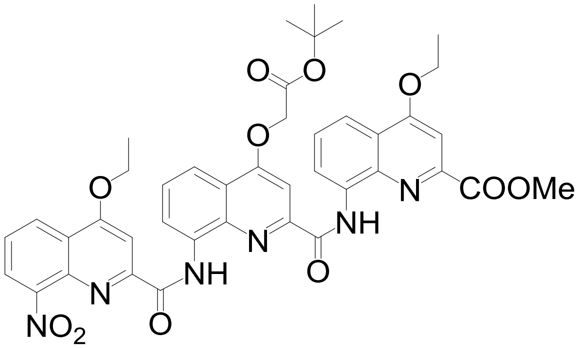

TLC (Hexane:Ethylacetate, 70:30 v/v): R_F=0.51; ^{1}H NMR (400 MHz, Chloroform-*d*) δ 12.22 – 12.20 (s, 1H), 12.19 – 12.16 (s, 1H), 9.04 – 8.99 (m, 1H), 8.98 – 8.91 (m, 1H), 8.51 – 8.43 (dd, *J* = 8.2, 1.4 Hz, 1H), 8.17 – 8.07 (m, 1H), 7.93 – 7.88 (m, 2H), 7.77 – 7.72 (s, 1H), 7.72 – 7.66 (td, *J* = 8.1, 3.8 Hz, 2H), 7.56 – 7.52 (dd, *J* = 7.6, 1.7 Hz, 1H), 7.38 – 7.32 (t, *J* = 7.9 Hz, 1H), 6.80 – 6.65 (s, 1H), 4.95 – 4.89 (s, 2H), 4.57 – 4.42 (q, *J* = 7.0 Hz, 2H), 4.18 – 4.02 (q, *J* = 7.0 Hz, 2H), 3.46 – 3.41 (s, 3H), 1.72-1.51 (m, 15H). MS (MALDI-TOF) calcd for $C_{41}H_{38}N_6O_{11}$ (M+H$^+$), 790.26, obsd: 789.93.

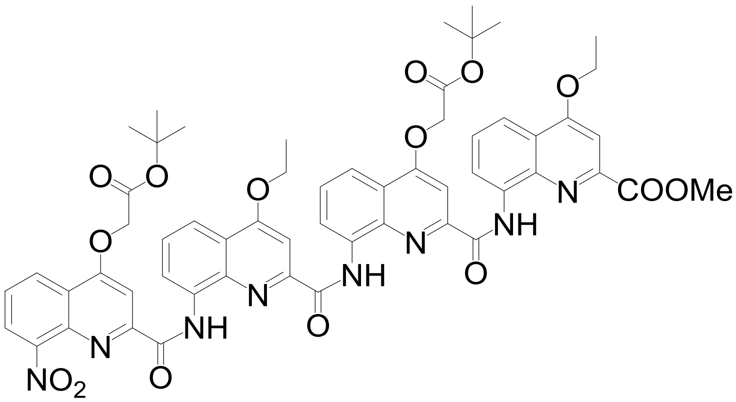

TLC (Hexane:Ethylacetate, 70:30 v/v): R_F=0.43; ^{1}H NMR (500 MHz, Chloroform-*d*) δ 12.33 – 12.27 (s, 1H), 11.92 – 11.87 (s, 1H), 11.71 – 11.62 (s, 1H), 9.22 – 9.12 (d, *J* = 7.6 Hz, 1H), 8.68 – 8.63 (dd, *J* = 8.3, 1.5

Hz, 1H), 8.39 – 8.33 (d, J = 7.5 Hz, 1H), 8.11 – 8.07 (dd, J = 8.5, 1.3 Hz, 1H), 8.02 – 7.97 (m, 3H), 7.95 – 7.90 (dd, J = 8.4, 1.3 Hz, 1H), 7.88 – 7.86 (s, 1H), 7.83 – 7.75 (t, J = 8.0 Hz, 1H), 7.64 – 7.60 (t, J = 8.2 Hz, 1H), 7.59 – 7.54 (m, 2H), 7.51 – 7.46 (t, J = 7.9 Hz, 1H), 6.84 – 6.78 (s, 1H), 6.75 – 6.66 (s, 1H), 5.05 – 5.00 (s, 2H), 4.73 – 4.67 (s, 2H), 4.22 – 4.13 (q, J = 6.9 Hz, 2H), 3.86 – 3.79 (q, J = 7.2 Hz, 3H), 3.46 – 3.46 (s, 3H), 1.75 – 1.68 (t, J = 7.0 Hz, 4H), 1.63 – 1.62 (s, 9H), 1.56 – 1.55 (s, 9H), 1.40 – 1.34 (t, J = 7.2 Hz, 3H). MS (MALDI-TOF) calcd for $C_{57}H_{54}N_8O_{15}$ (M+H$^+$), 1191.10, obsd: 1190.81.

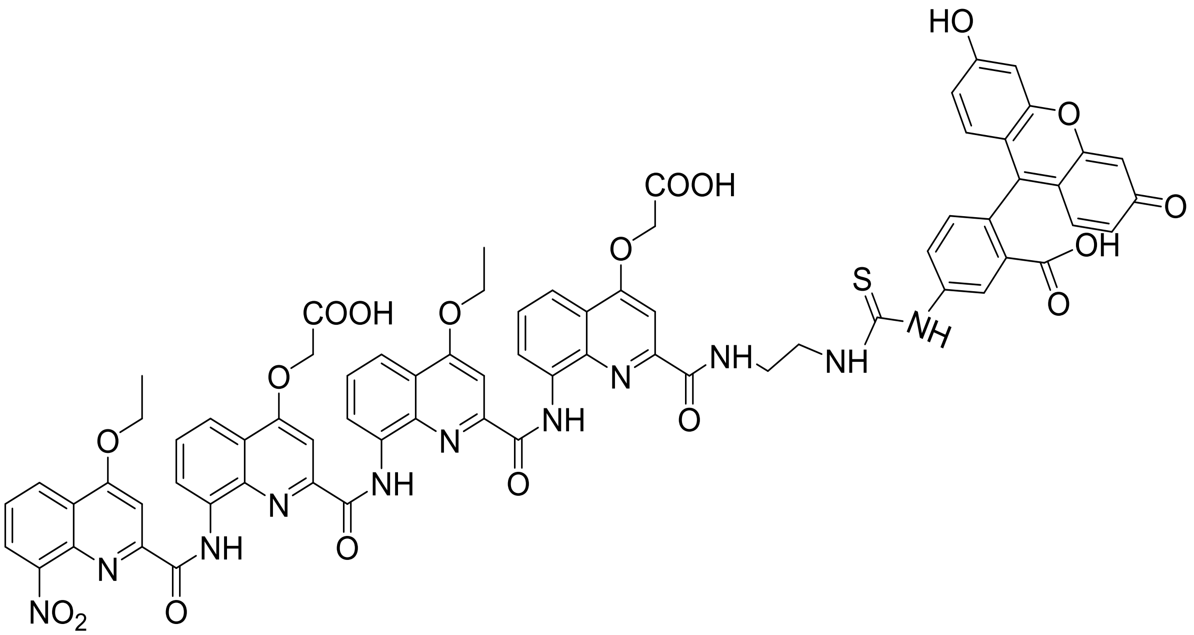

^{1}H NMR (600 MHz, DMSO-d_6) δ 11.77 (s, 1H), 11.74 (s, 1H), 10.51 – 10.41 (s, 3H), 8.91 – 8.77 (dd, J = 8.1, 2.6 Hz, 1H), 8.63 – 8.53 (d, J = 8.6 Hz, 1H), 8.17 – 8.12 (m, 1H), 8.12 – 8.07 (dd, J = 9.0, 4.4 Hz, 2H), 7.86 – 7.81 (dd, J = 8.3, 2.2 Hz, 4H), 7.61 – 7.57 (m, 1H), 7.57 – 7.54 (d, J = 4.1 Hz, 1H), 7.48 – 7.39 (dt, J = 15.6, 7.8 Hz, 2H), 7.25 – 7.22 (d, J = 8.2 Hz, 2H), 7.18 – 7.15 (m, 1H), 7.15 – 7.11 (m, 2H), 6.66 – 6.65 (d, J = 2.5 Hz, 2H), 6.61 – 6.60 (s, 1H), 6.60 – 6.59 (s, 1H), 6.57 – 6.54 (dd, J = 8.5, 2.1 Hz, 2H), 3.75 – 3.70 (q, J = 6.2 Hz, 2H), 3.64 – 3.52 (dt, J = 14.6, 7.2 Hz, 4H), 3.45 – 3.35 (dt, J = 15.2, 5.6 Hz, 4H), 3.23 – 3.17 (p, J = 7.0 Hz, 2H), 1.23 – 1.20 (t, J = 7.1 Hz, 3H), 0.99 – 0.96 (t, J = 7.5 Hz, 3H). MS (MALDI-TOF) calcd for $C_{71}H_{53}N_{11}O_{19}S$ (M+H$^+$), 1396.3318, obsd: 1396.3367.

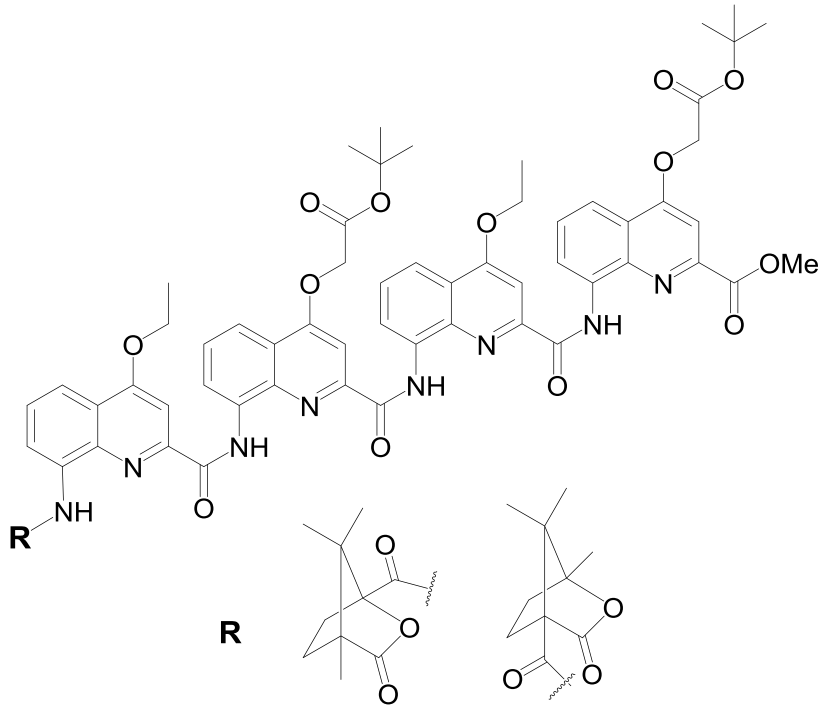

To a solution of 14 (26.5 mg, 25 μM) in chloroform (5 mL, anhydrous), trimethylamine (7 μL, anhydrous) was added and the reaction was stirred at 0 °C for 15 min. under inert atmosphere. To this reaction mixture, a slution of (1S)-(-) camphanyl chloride or (1R)-(+) camphanyl chloride (5.5 mg, 25 μM) in $CHCl_3$ (3 mL, anhydrous) was added dropwise at 0 °C under inert atmosphere. The reaction mixture was stirred for 15 min. at 0 °C and then for 24 h at r.t. The solvents were removed on rotovap and the desired product was purified using column chromatography (0 to 50% ethylacetate in hexane, v/v) as a pale yellow solid (see Table 1 for % yield). TLC (Hexane:Ethylacetate, 50:50 v/v): R_F=0.45; ^{1}H NMR (400 MHz, Chloroform-*d*) δ 12.49 – 12.43 (s, 1H), 11.89 – 11.83 (s, 1H), 11.80 – 11.71 (s, 1H), 9.94 – 9.84 (s, 1H), 9.06 – 8.93 (m, 1H), 8.60 – 8.51 (m, 1H), 8.09 – 8.04 (dd, *J* = 8.3, 1.3 Hz, 1H), 8.02 – 7.94 (m, 3H), 7.93 – 7.91 (s, 1H), 7.71 – 7.66 (t, *J* = 8.0 Hz, 2H), 7.65 – 7.59 (t, *J* = 8.0 Hz, 1H), 7.59 – 7.56 (s, 1H), 7.54 – 7.49 (m, 1H), 7.41 – 7.39 (s, 1H), 7.34 – 7.28 (m, 1H), 6.80 – 6.76 (s, 1H), 6.62 – 6.60 (s, 1H), 4.94 – 4.84 (d, *J* = 2.9 Hz, 2H), 4.72 – 4.58 (m, 3H), 4.58 – 4.45 (m, 2H), 4.27 – 4.11 (dq, *J* = 9.7, 3.3 Hz, 2H), 3.53 – 3.45 (s, 3H), 2.35 – 2.22 (m, 2H), 2.08 – 1.95 (m, 1H),

1.83 – 1.72 (t, J = 7.0 Hz, 4H), 1.62 – 1.56 (s, 11H), 1.57 – 1.49 (s, 11H), 1.43 – 1.36 (s, 4H), 0.80 – 0.76 (s, 3H), 0.75 – 0.71 (s, 3H). MS (MALDI-TOF) calcd for $C_{67}H_{68}N_8O_{16}$ (M+H$^+$), 1240.47, obsd: 1240.17.

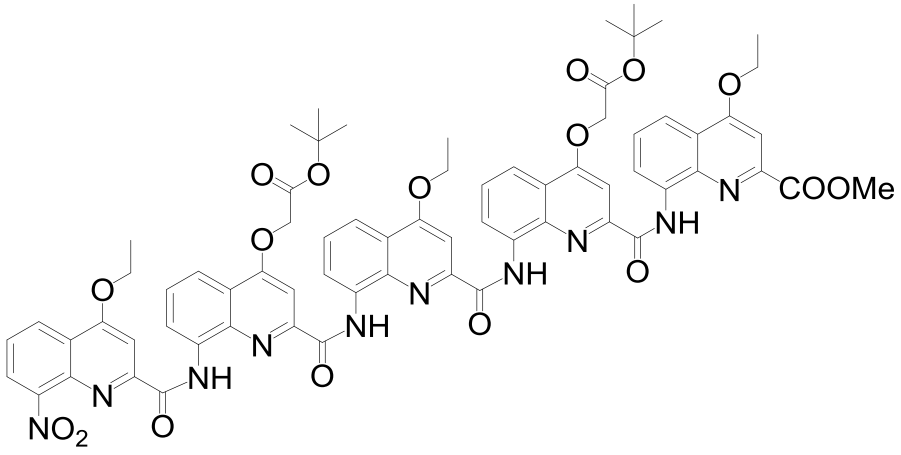

TLC (Hexane:Ethylacetate, 60:40 v/v): R_F=0.38; ^{1}H NMR (500 MHz, Chloroform-*d*) δ 11.98 – 11.95 (s, 1H), 11.77 – 11.74 (s, 1H), 11.74 – 11.72 (s, 1H), 11.57 – 11.52 (s, 1H), 8.66 – 8.60 (dd, J = 8.3, 1.4 Hz, 1H), 8.52 – 8.47 (ddd, J = 7.5, 4.8, 1.3 Hz, 2H), 8.23 – 8.16 (ddd, J = 11.9, 7.6, 1.2 Hz, 2H), 8.13 – 8.08 (dd, J = 8.3, 1.2 Hz, 1H), 8.08 – 8.06 (dd, J = 2.2, 1.2 Hz, 1H), 8.06 – 8.01 (m, 2H), 7.96 – 7.92 (dd, J = 8.2, 1.3 Hz, 1H), 7.71 – 7.63 (dt, J = 16.0, 8.0 Hz, 2H), 7.59 – 7.51 (m, 2H), 7.51 – 7.50 (s, 1H), 7.47 – 7.42 (m, 2H), 7.37 – 7.31 (t, J = 8.0 Hz, 1H), 6.86 – 6.82 (s, 1H), 6.75 – 6.71 (s, 1H), 6.54 – 6.51 (s, 1H), 5.07 – 5.01 (d, J = 5.1 Hz, 2H), 5.02 – 4.96 (dq, J = 17.2, 1.7 Hz, 1H), 4.95 – 4.91 (ddt, J = 10.0, 2.3, 1.3 Hz, 1H), 4.71 – 4.68 (d, J = 5.4 Hz, 1H), 4.67 – 4.64 (d, J = 2.9 Hz, 2H), 4.60 – 4.53 (dd, J = 15.6, 8.9 Hz, 2H), 4.52 – 4.48 (ddd, J = 6.0, 3.5, 1.2 Hz, 3H), 1.83 – 1.74 (m, 6H), 1.69 – 1.63 (s, 9H), 1.65 – 1.61 (s, 9H), 1.55 – 1.50 (s, 9H). MS (MALDI-TOF) calcd for $C_{73}H_{70}N_{10}O_{19}$ (M+H$^+$), 1391.41, obsd: 1391.50.

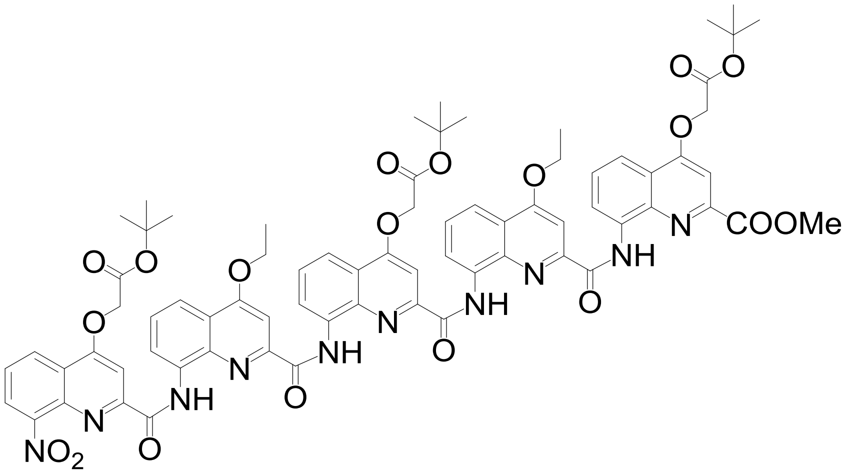

TLC (Hexane:Ethylacetate, 50:50 v/v): R_F=0.42; ^{1}H NMR (400 MHz, Chloroform-*d*) δ 11.98 – 11.95 (s, 1H), 11.78 – 11.72 (d, *J* = 3.1 Hz, 2H), 11.56 – 11.51 (s, 1H), 8.59 – 8.54 (dd, *J* = 8.3, 1.5 Hz, 1H), 8.54 – 8.44 (m, 2H), 8.24 – 8.14 (ddd, *J* = 15.6, 8.1, 1.2 Hz, 2H), 8.14 – 8.07 (ddd, *J* = 8.4, 3.5, 1.2 Hz, 2H), 8.05 – 7.95 (ddd, *J* = 17.3, 8.4, 1.2 Hz, 2H), 7.85 – 7.80 (dd, *J* = 8.4, 1.2 Hz, 1H), 7.70 – 7.61 (m, 2H), 7.50 – 7.49 (d, *J* = 1.6 Hz, 1H), 7.42 – 7.38 (m, 2H), 7.38 – 7.34 (m, 2H), 6.80 – 6.77 (s, 1H), 6.76 – 6.73 (s, 1H), 6.59 – 6.56 (s, 1H), 5.07 – 5.01 (d, *J* = 2.6 Hz, 2H), 4.76 – 4.69 (d, *J* = 8.6 Hz, 2H), 4.52 – 4.44 (m, 2H), 4.14 – 4.07 (m, 2H), 3.92 – 3.82 (m, 2H), 3.20 – 3.17 (s, 3H), 1.85 – 1.73 (t, *J* = 7.0 Hz, 3H), 1.67 – 1.64 (d, *J* = 7.0 Hz, 3H), 1.62 – 1.61 (s, 10H), 1.60 – 1.57 (d, *J* = 6.7 Hz, 3H), 1.53 – 1.51 (s, 9H). MS (MALDI-TOF) calcd for $C_{69}H_{64}N_{10}O_{17}$ (M+H$^+$), 1305.32, obsd: 1305.72.

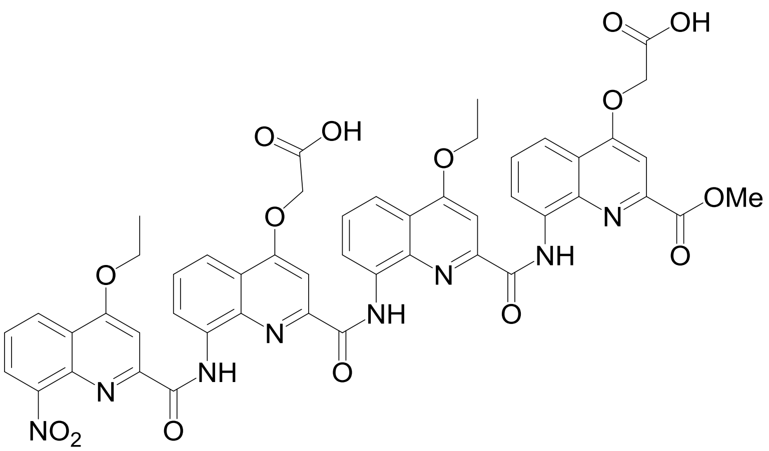

^{1}H NMR (600 MHz, DMSO-d_6) δ 13.69 – 13.15 (s, 2H), 12.12 – 12.03 (s, 1H), 11.69 – 11.59 (s, 1H), 11.53 – 11.43 (s, 1H), 9.02 – 8.99 (dd, *J* = 7.5, 1.3 Hz, 1H), 8.54 – 8.51 (dd, *J* = 8.2, 1.4 Hz, 1H), 8.41 – 8.38 (dd, *J* = 7.5, 1.3 Hz, 1H), 8.01 – 7.97 (dd, *J* = 7.6, 1.3 Hz, 1H), 7.95 – 7.92 (dd, *J* = 8.4, 1.3 Hz, 1H), 7.92 – 7.90 (dd, *J* = 8.4, 1.3 Hz, 1H), 7.89 – 7.86 (dd, *J* = 8.4, 1.3 Hz, 1H), 7.80 –

7.79 (d, J = 2.7 Hz, 1H), 7.79 – 7.75 (m, 2H), 7.72 – 7.68 (dd, J = 7.5, 1.4 Hz, 1H), 7.63 – 7.59 (dd, J = 8.3, 7.5 Hz, 1H), 7.50 – 7.46 (m, 1H), 7.35 – 7.30 (s, 1H), 6.85 – 6.80 (s, 1H), 6.61 – 6.57 (s, 1H), 5.39 – 5.07 (m, 2H), 5.03 – 4.76 (m, 2H), 4.76 – 4.50 (m, 2H), 4.39 – 4.08 (dd, J = 68.0, 11.0 Hz, 2H), 3.35 – 3.32 (s, 3H), 1.73 – 1.68 (t, J = 7.0 Hz, 3H), 1.54 – 1.49 (t, J = 7.0 Hz, 3H). HRMS-ESI (m/z): calculated for $C_{49}H_{39}N_8O_{15}^+$ [(M+H)+]: 979.2529, found 979.2530.

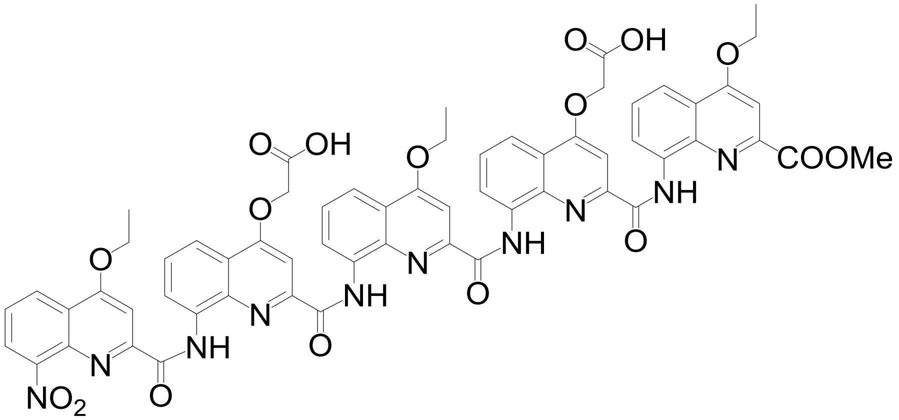

^{1}H NMR (500 MHz, DMSO-d_6) δ 14.04 – 12.77 (s, 3H), 11.69 – 11.61 (s, 1H), 11.54 – 11.48 (s, 1H), 11.48 – 11.43 (s, 1H), 11.34 – 11.27 (s, 1H), 8.51 – 8.41 (m, 2H), 8.40 – 8.32 (d, J = 7.5 Hz, 1H), 8.02 – 7.98 (d, J = 7.6 Hz, 1H), 7.98 – 7.95 (d, J = 8.4 Hz, 1H), 7.94 – 7.90 (d, J = 8.3 Hz, 1H), 7.87 – 7.84 (d, J = 8.3 Hz, 1H), 7.83 – 7.76 (m, 2H), 7.75 – 7.71 (t, J = 8.0 Hz, 1H), 7.70 – 7.66 (d, J = 8.2 Hz, 1H), 7.55 – 7.50 (m, 2H), 7.50 – 7.45 (t, J = 8.0 Hz, 1H), 7.37 – 7.33 (t, J = 8.0 Hz, 1H), 7.32 – 7.30 (s, 1H), 7.21 – 7.15 (s, 1H), 6.76 – 6.68 (s, 1H), 6.68 – 6.62 (s, 1H), 6.44 – 6.39 (s, 1H), 5.25 – 5.17 (d, J = 4.0 Hz, 2H), 4.98 – 4.90 (d, J = 16.1 Hz, 1H), 4.88 – 4.79 (m, 2H), 4.74 – 4.61 (m, 2H), 4.62 – 4.53 (m, 1H), 4.33 – 4.22 (dq, J = 9.6, 7.0 Hz, 1H), 4.18 – 4.09 (dq, J = 9.5, 6.8 Hz, 1H), 3.09 – 2.98 (s, 3H), 1.72 – 1.63 (t, J = 6.9 Hz, 3H), 1.56 – 1.46 (t, J = 6.9 Hz, 3H). HRMS-ESI (m/z): calculated for $C_{61}H_{47}N_{10}O_{19}^+$ [(M+H)+]: 1223.3031, found 1223.3031.

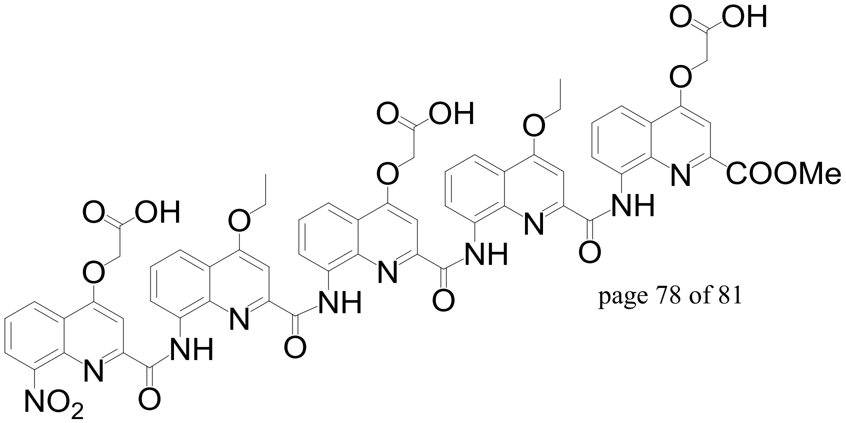

^{1}H NMR (400 MHz, DMSO-d_6) δ 13.91 – 12.89 (br, s, 2H), 11.71 – 11.66 (s, 1H), 11.58 – 11.51 (d, J =

9.4 Hz, 2H), 11.36 – 11.31 (s, 1H), 8.55 – 8.46 (m, 2H), 8.40 – 8.33 (d, *J* = 7.5 Hz, 1H), 8.07 – 7.93 (m, 3H), 7.91 – 7.78 (m, 3H), 7.77 – 7.68 (m, 2H), 7.60 – 7.48 (m, 3H), 7.42 – 7.33 (m, 2H), 7.23 – 7.18 (s, 1H), 6.79 – 6.74 (s, 1H), 6.70 – 6.65 (s, 1H), 6.54 – 6.49 (s, 1H), 5.28 – 5.21 (d, *J* = 4.8 Hz, 2H), 5.02 – 4.84 (m, 3H), 4.76 – 4.59 (ddd, *J* = 32.6, 9.6, 7.0 Hz, 2H), 4.27 – 4.06 (m, 4H), 3.15 – 3.07 (s, 3H), 1.79 – 1.66 (t, *J* = 6.9 Hz, 3H), 1.60 – 1.48 (dt, *J* = 13.6, 6.9 Hz, 6H). HRMS-ESI (m/z): calculated for $C_{61}H_{49}N_{10}O_{17}^{+}$ [(M+H)+]: 1193.3272, found 1193.3274.

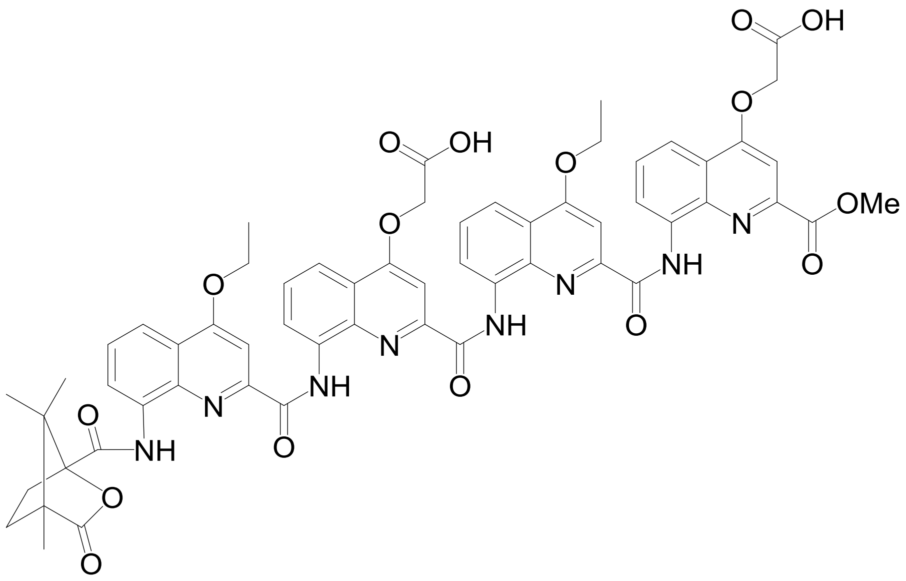

^{1}H NMR (600 MHz, DMSO-d_6) δ 12.25 – 12.20 (s, 1H), 11.65 – 11.61 (s, 1H), 11.61 – 11.56 (s, 1H), 9.80 – 9.69 (s, 1H), 8.98 – 8.88 (d, *J* = 7.4 Hz, 1H), 8.50 – 8.44 (d, *J* = 7.6 Hz, 1H), 7.93 – 7.87 (m, 2H), 7.87 – 7.82 (m, 3H), 7.82 – 7.75 (m, 2H), 7.73 – 7.68 (t, *J* = 8.0 Hz, 1H), 7.63 – 7.59 (s, 1H), 7.44 – 7.36 (dt, *J* = 13.7, 7.9 Hz, 2H), 7.27 – 7.22 (s, 1H), 6.77 – 6.72 (s, 1H), 6.58 – 6.55 (s, 1H), 5.23 – 4.92 (d, *J* = 35.7 Hz, 4H), 4.72 – 4.51 (m, 3H), 4.34 – 4.17 (m, 2H), 2.03 – 1.89 (ddd, *J* = 18.8, 9.7, 5.3 Hz, 3H), 1.74 – 1.66 (t, *J* = 7.0 Hz, 3H), 1.61 – 1.51 (t, *J* = 6.9 Hz, 4H), 1.49 – 1.30 (m, 2H), 0.86 – 0.78 (t, *J* = 6.9 Hz, 2H), 0.72 – 0.67 (s, 3H), 0.67 – 0.63 (s, 3H). HRMS-ESI (m/z): calculated for $C_{59}H_{53}N_8O_{16}^{+}$ [(M+H)+]: 1129.3574, found 1129.3575.

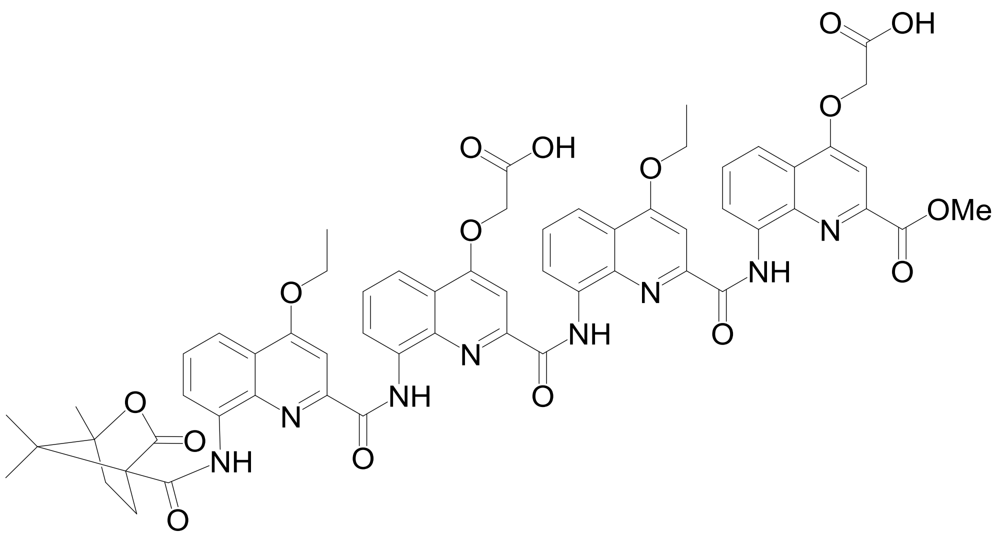

^{1}H NMR (600 MHz, DMSO-d_6) δ 12.24 – 12.19 (s, 1H), 11.66 – 11.61 (s, 1H), 11.58 – 11.54 (s, 1H), 9.77 – 9.71 (s, 1H), 8.95 – 8.92 (d, *J* = 7.5 Hz, 1H), 8.46 – 8.41 (d, *J* = 7.5 Hz, 1H), 7.91 – 7.87 (d, *J* = 8.4 Hz, 2H), 7.87 – 7.82 (m, 3H), 7.81 – 7.78 (d, *J* = 7.6 Hz, 1H), 7.76 – 7.72 (t, *J* = 7.9 Hz, 1H), 7.72 – 7.68 (t, *J* = 7.9 Hz, 1H), 7.59 – 7.54 (s, 1H), 7.42 – 7.36 (t, *J* = 8.0 Hz, 2H), 7.28 – 7.24 (s, 1H), 6.76 – 6.71 (s, 1H), 6.55 – 6.51 (s, 1H), 5.02 – 4.84 (m, 2H), 4.73 – 4.52 (ddd, *J* = 68.5, 9.3, 7.0 Hz, 2H), 4.53 – 4.40 (d, *J* = 23.7 Hz, 2H), 4.33 – 4.19 (m, 2H), 3.40 – 3.37 (s, 3H), 2.01 – 1.98 (d, *J* = 7.5 Hz, 3H), 1.73 – 1.67 (t, *J* = 6.9 Hz, 4H), 1.60 – 1.54 (t, *J* = 7.0 Hz, 4H), 1.46 – 1.40 (t, *J* = 7.2 Hz, 2H), 1.09 – 1.04 (d, *J* = 6.6 Hz, 3H). HRMS-ESI (m/z): calculated for $C_{59}H_{53}N_8O_{16}^+$ [(M+H)+]: 1129.3574, found 1129.3578.

Supplementary References

1. Kumar, S., Brown, M., Nath, A. & Miranker, A. Folded Small Molecule Manipulation of Islet Amyloid Polypeptide. *Chem. Biol.* **21**, 775-781 (2014).
2. Kumar, S. *et al*. Islet Amyloid-Induced Cell Death and Bilayer Integrity Loss Share a Molecular Origin Targetable with Oligopyridylamide-Based α-Helical Mimetics. *Chem. Biol.* **22**, 369-378 (2015).
